# Characterization of the gut microbiome in a porcine model of thoracic spinal cord injury

**DOI:** 10.1186/s12864-021-07979-3

**Published:** 2021-10-30

**Authors:** Adam Doelman, Seth Tigchelaar, Brian McConeghy, Sunita Sinha, Martin S. Keung, Neda Manouchehri, Megan Webster, Shera Fisk, Charlotte Morrison, Femke Streijger, Corey Nislow, Brian K. Kwon

**Affiliations:** 1 International Collaboration on Repair Discoveries, University of British Columbia, Vancouver, BC Canada; 2 Sequencing and Bioinformatics Consortium, University of British Columbia, Vancouver, BC Canada; 3 Department of Orthopedics, Vancouver Spine Surgery Institute, University of British Columbia, Vancouver, BC Canada

**Keywords:** Spinal Cord Injury, Microbiome, Pig, Antibiotic, Diet, Gut-brain Axis

## Abstract

**Background:**

The gut microbiome is a diverse network of bacteria which inhabit our digestive tract and is crucial for efficient cellular metabolism, nutrient absorption, and immune system development. Spinal cord injury (SCI) disrupts autonomic function below the level of injury and can alter the composition of the gut microbiome. Studies in rodent models have shown that SCI-induced bacterial imbalances in the gut can exacerbate the spinal cord damage and impair recovery. In this study we, for the first time, characterized the composition of the gut microbiome in a Yucatan minipig SCI model. We compared the relative abundance of the most dominant bacterial phyla in control samples to those collected from animals who underwent a contusion-compression SCI at the 2nd or 10th Thoracic level.

**Results:**

We identify specific bacterial fluctuations that are unique to SCI animals, which were not found in uninjured animals given the same dietary regimen or antibiotic administration. Further, we identified a specific time-frame, “SCI-acute stage”, during which many of these bacterial fluctuations occur before returning to “baseline” levels.

**Conclusion:**

This work presents a dynamic view of the microbiome changes that accompany SCI, establishes a resource for future studies and to understand the changes that occur to gut microbiota after spinal cord injury and may point to a potential therapeutic target for future treatment.

**Supplementary Information:**

The online version contains supplementary material available at 10.1186/s12864-021-07979-3.

## Background

The gut microbiome is a diverse network of bacteria which inhabit our digestive tract. This collection of microbes consists of beneficial (probionts) and pathogenic (pathobionts) bacteria, whose concentrations must be carefully maintained to function symbiotically within the host. Today, we recognize that gut microbiota are critical for a number of key physiological processes such as the development and maintenance of cellular metabolism, nutrient absorption, and immune system development [1–3]. Further, there is increasing interest in the inter-dependent communication pathway which exists between the gut microbiome, the immune system, and the central nervous system (CNS), referred to commonly as the “gut-brain axis” or “gut-CNS axis”. The CNS can influence the composition of the gut microbiome via the autonomic nervous system by modulating gut motility, intestinal transit times, gut permeability and through the luminal secretion of various hormones [4]. Conversely, bacteria residing in the intestinal tract can “communicate” with the CNS directly via immune cells or nerve fibers as well as indirectly by secreting neuroactive metabolites (such as short chain fatty acids [SCFAs] and choline) produced by the fermentation of microbiome-accessible carbohydrates [5, 6]. These neuroactive metabolites can then cross the intestinal barrier, enter systemic circulation and potentially cross the blood-brain barrier to influence neural activity and inflammation [7–9].

Spinal cord injury (SCI) is a life-altering occurrence affecting approximately 250,000 people in the United States alone, with between 11,000 and 17,000 new incidents occurring each year [10]. In addition to causing obvious impairments in motor and sensory function, SCI disrupts autonomic function below the level of injury. For example, it has become increasingly recognized that amongst the myriad of effects of SCI, it can cause significant perturbations in the gut microbiome. Today, we are beginning to understand the role of the gut microbiome as a disease-modifying factor following traumatic SCI due to the impaired immune-response seen in SCI animals [11]. For instance, Kigerl et al. showed that SCI-induced gut dysbiosis is associated with a change in the proportion of immune cells found in mesenteric lymph nodes and that this imbalance can significantly affect recovery after injury [12]. To better understand how SCI induces cellular and molecular changes to lymphoid tissue and other immune responses in the gut after injury, we must first characterize how gut bacteria are affected by SCI.

Given the complications in establishing a suitably translatable SCI-gut microbiome model system, we sought to determine the effect of SCI on gut microbiota using an established porcine model of SCI. Swine has been deemed an excellent translational model in reference to digestive physiology, nutrition and dietary behavior due to stark similarities in terms of mesenteric vasculature, functional structural colon segments and relative length, dietary requirements, enzyme activity profiles as well as GI transit times of pharmaceuticals [13, 14]. Pigs are also omnivores and in our animal care facility, consume their food in meals at scheduled times as opposed to consuming small amounts all day, which makes this an ideal model for examining the effect of dietary manipulation on gut microbes. Acknowledging the translational potential of porcine species, miniature swine has emerged as an attractive model to assess the microbiome as their weight to digestive length is more equivalent to an average human, while maintaining the same digestive physiology and microbial composition [15, 16]. For instance, Ossabaw and Göttingen minipigs are now considered excellent models to assess the link between diet and various pathological outcomes including obesity, diabetes and metabolic syndrome [17, 18].

Here we used a porcine model (Yucatan) to investigate the effect of contusive/compressive SCI on the composition of the gut microbiome before and up to 7 weeks after injury. To the best of our knowledge, the gut microbiota of Yucatan minipigs has not been characterized previously. Our goal was therefore to determine a baseline composition of the gut microbiome in our established Yucatan pig model of SCI [19, 20] and examine the effects of severe thoracic SCI longitudinally. Further, we sought to characterize the disruption that may be induced by our standard “post-surgical diet” or antibiotic treatment on non-SCI animals in an attempt to isolate the effect of SCI from other factors known to induce gut bacterial dysbiosis.

## Results

**Fig. 1:**
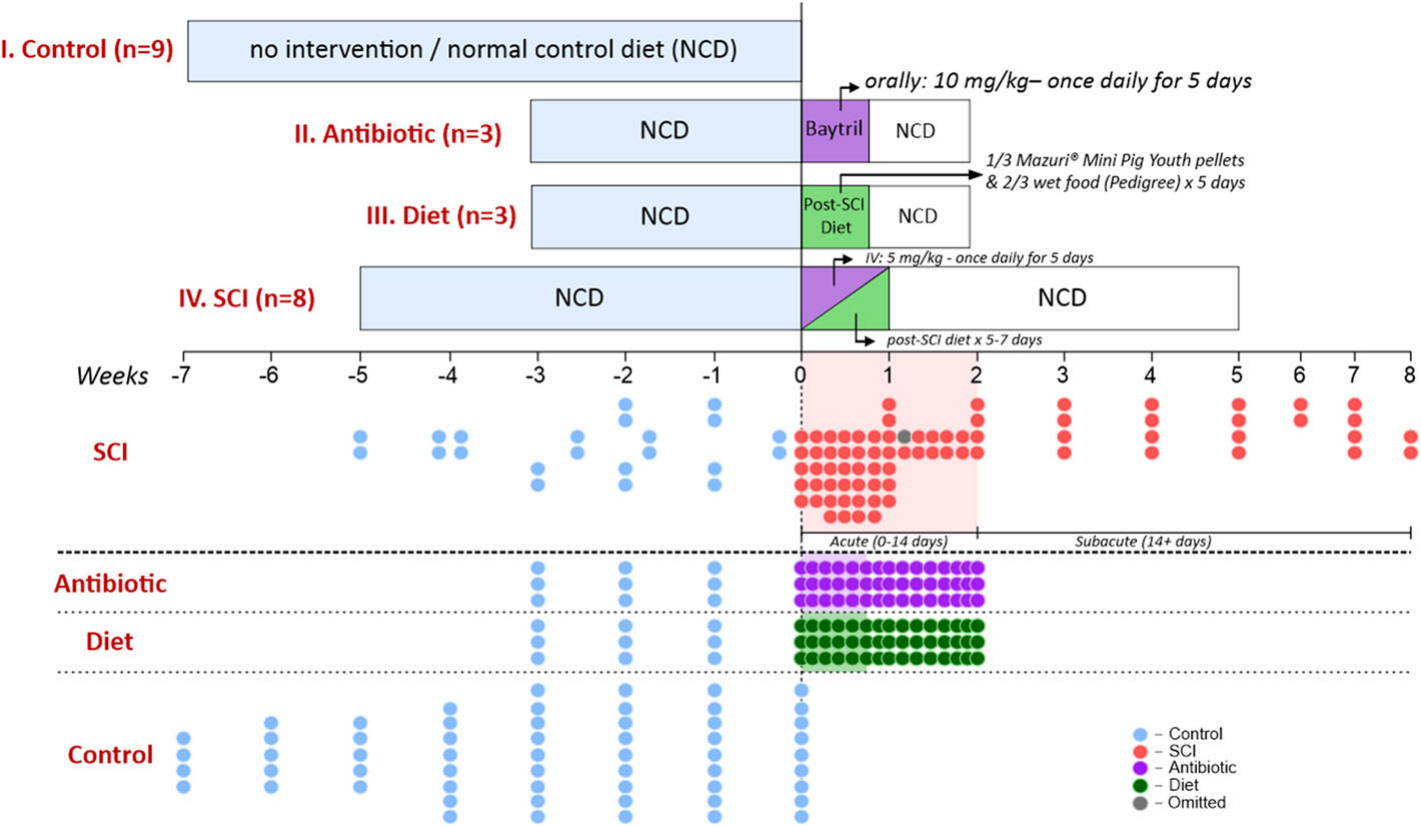
Longitudinal characterization of bacterial abundance at the phylum level in SCI animals (*n*=8). The individual median CLR values are plotted along with the associated loess curves (Local Polynomial Regression Fitting) with confidence intervals in gray. Blue values represent control samples, Red values represent SCI samples. Black dotted line indicates SCI surgery date

In this longitudinal study, microbiome composition determined pre-SCI was compared to microbiome composition up to 7–8 weeks thereafter. Twenty-three Yucatan pigs were divided into four groups: Control (*n* = 9), Diet (*n* = 3), Antibiotic (*n* = 3), and SCI (*n* = 8). gDNA samples (*n* = 192) were extracted from a total of 262 porcine fecal samples and the bacterial microbiome composition was determined with 16S rRNA sequencing. Samples from two SCI pigs > 49 days after injury were omitted because a second, non-SCI surgery was performed 52 days after the initial spinal insult. A single sample from one of the animals at 8 days post-SCI was omitted as an outlier due to a stark compositional dissimilarity (confirmed by Q test) between samples collected from this animal at 7- and 9-days post-SCI. This left 93 samples in the control setting, 45 samples in the diet and antibiotic groups, and 78 samples collected from SCI animals (see Fig. 1).

### Composition, stability and diversity of intestinal microbiome of uninjured Yucatan Minipigs

**Fig. 2:**
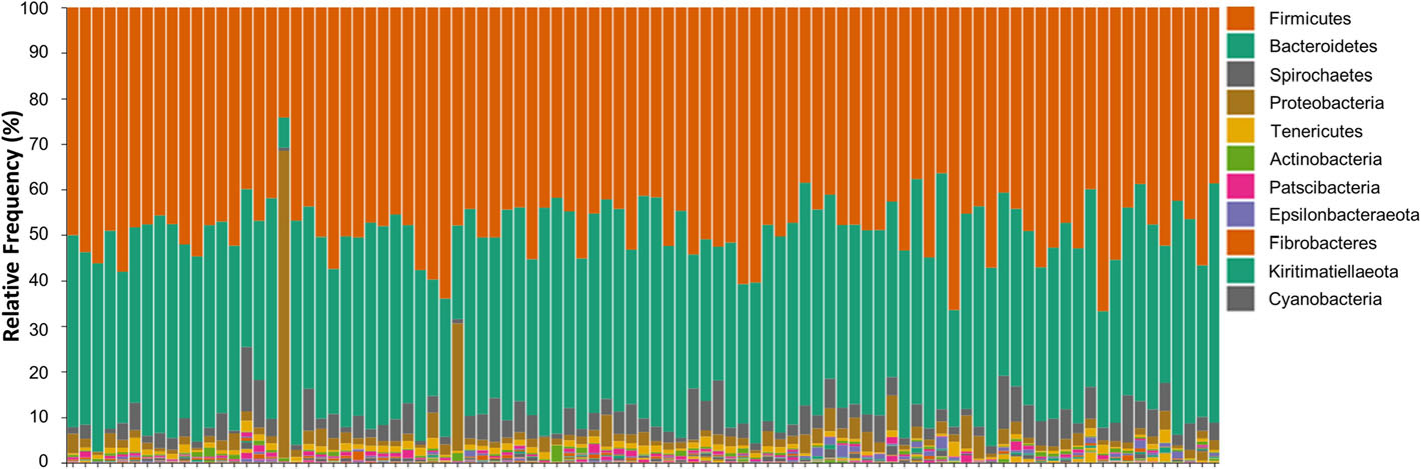
Longitudinal characterization of bacterial abundance at the phylum level in antibiotic-treated animals (*n*=3); The individual median CLR values are plotted along with the associated loess curves (Local Polynomial Regression Fitting) with confidence intervals in gray. Blue values represent control samples, Purple values represent samples collected from antibiotic-treated animals. Black dotted line indicates start of treatment

Phylum level taxonomy of the most dominant bacterial populations in the control setting are presented in Fig. 2. In Yucatan minipigs, we found that approximately 98% of the total bacterial abundance was classified into 6 phyla. We found the majority of bacterial species belong to the *Firmicutes* and *Bacteroidetes* phyla, comprising approximately 90% of all bacteria in porcine feces. A smaller fraction of bacteria belongs to the *Spirochaetes* (4.24%), *Proteobacteria* (2.23%), *Tenericutes* (1.01%) and *Actinobacteria* (0.47%) phyla.

For each phylum, we established a “baseline range” by assembling the centered log-ratio (CLR) transformed values of all fecal samples collected from the Control group as well as those collected before treatment from the four groups and plotted them longitudinally. This range represents the expected microbial composition of Yucatan pigs at the phylum level which can serve as a reference dataset for future microbial analyses as well as the expected stability of this phylum in untreated pigs. Herein, we utilize a baseline range to compare our expected CLR values to those obtained after treatment to infer whether the alterations can be considered significantly different.

### Spinal cord injury induces time-dependent fluctuations in the gut microbiome of Yucatan Minipigs

Due to considerable temporal differences in CLR values of the most dominant bacterial phyla noted following SCI, particularly within the first 2 weeks after injury, we decided to divide the SCI samples into two phases, acute (0–14 days post-SCI) and subacute (> 14 days post-SCI) and analyze them as separate treatment groups.

**Fig. 3:**
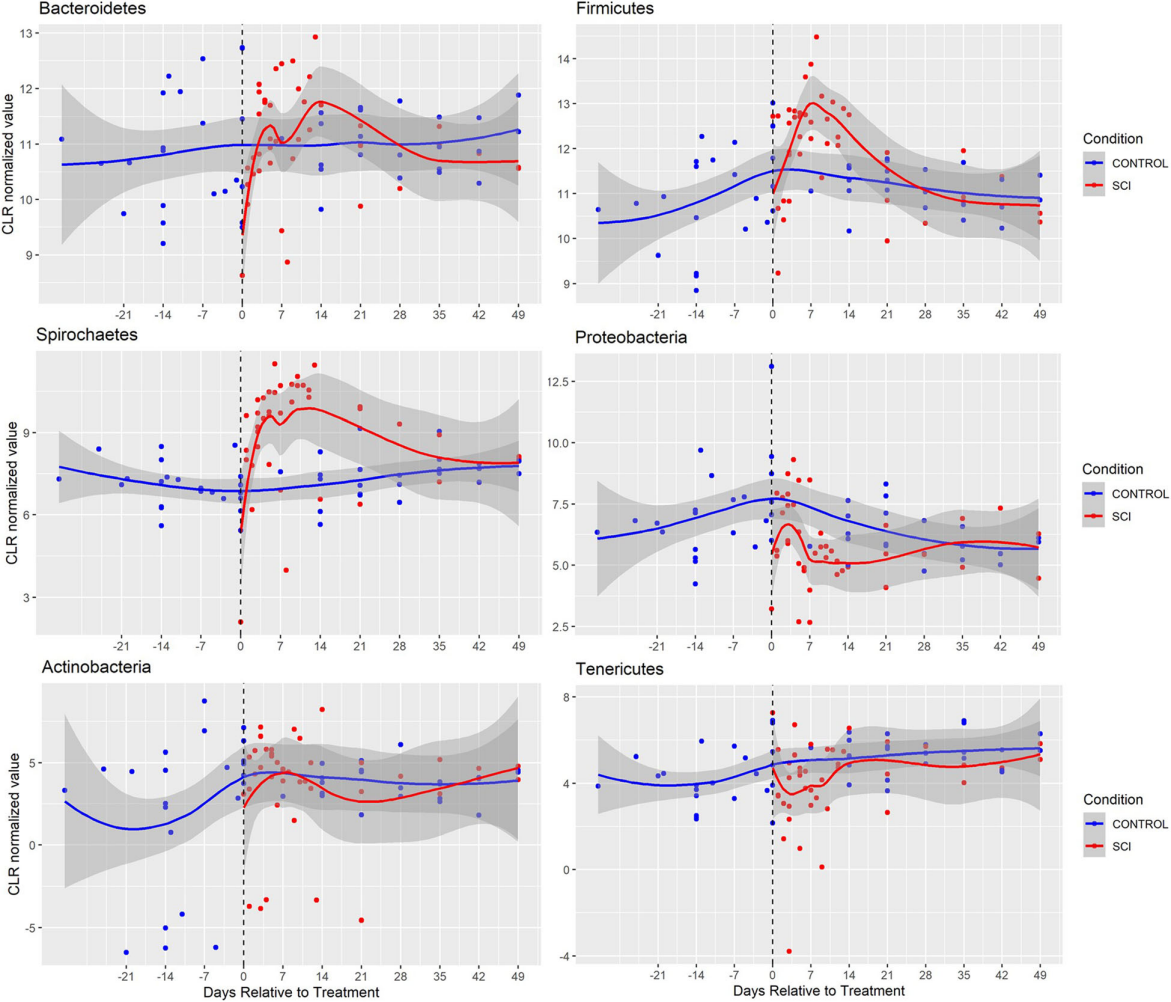
Longitudinal characterization of bacterial abundance at the phylum level in animals who underwent post-surgical diet regiment (*n*=3); The individual median CLR values are plotted along with the associated loess curves (Local Polynomial Regression Fitting) with confidence intervals in gray. Blue values represent control samples, Green values represent samples collected from animals fed the standard post-surgical diet. Black dotted line indicates start of treatment

**Fig. 4:**
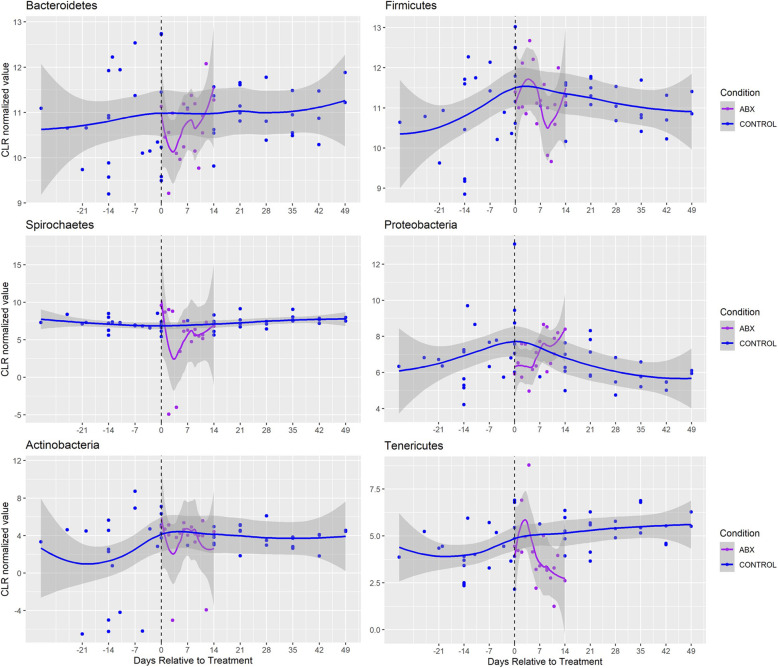
Functional inference analysis comparing SCI and control specimens using PICRUSt2.**A**) PCA Biplot. A biplot is a combination of a samples PCA plot and a loadings plot that showshow strongly each characteristic influences a principal component. Boxed names are theloadings. Ellipses represent the default 95-level assuming a multivariate t-distribution. **B**-**E**)Longitudinal characterization of bacterial abundance of the 4 pathways with the strongestnegative (B,C) and positive effect (D,E) size. The individual median CLR values are plotted alongwith the associated loess curves (Local Polynomial Regression Fitting) with confidence intervalsin gray. Red = SCI, Blue = Control

**Fig. 5:**
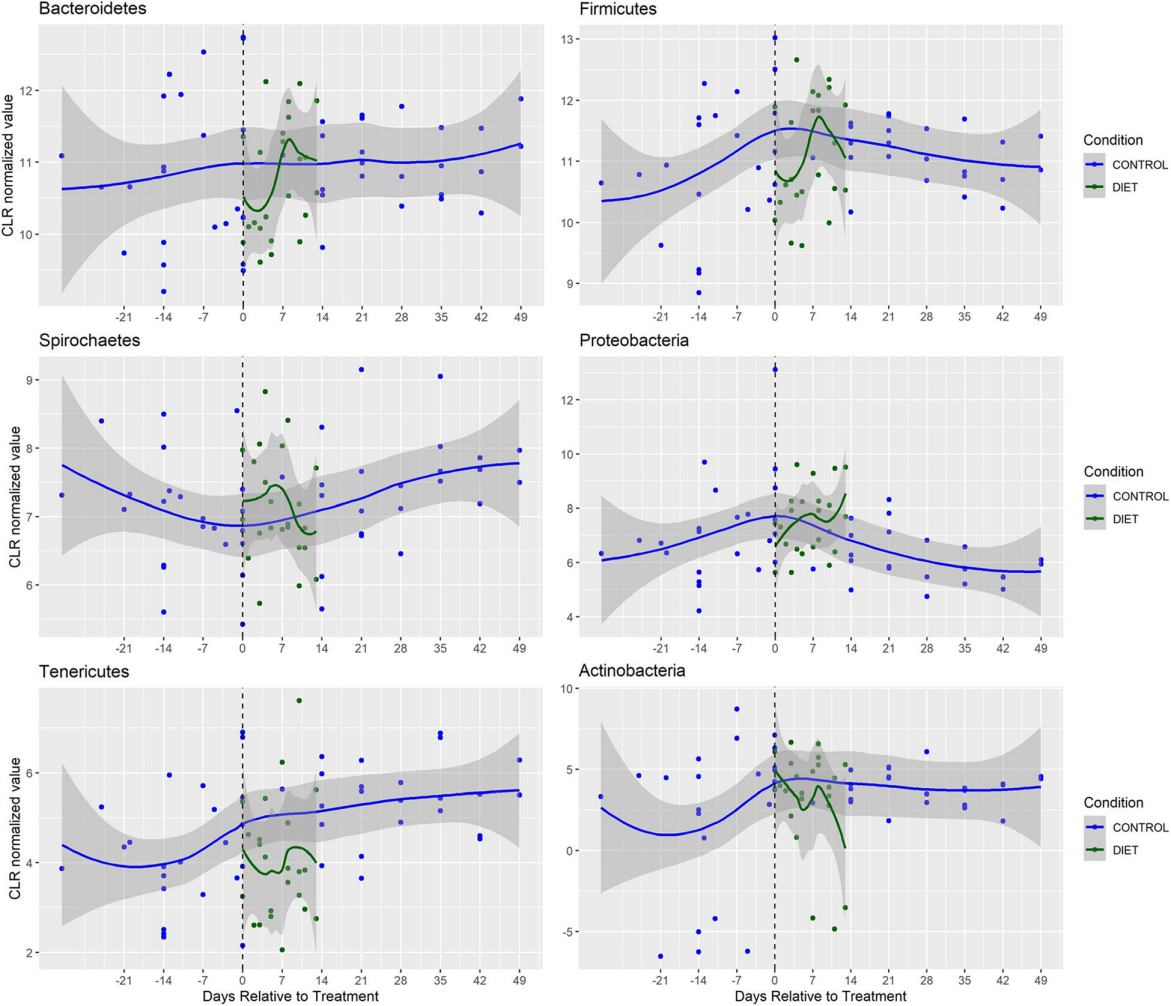
Alpha diversity box plots (species richness) of our 4 treatment groups (SCI divided into SCI acute and SCI Subacute), and control. Groups were compared using Kruskal-Wallis analysis. Increases in the number of bacterial species present in a given ecosystem indicates an increase in species richness. *p<0.05; **p<0.01; ***p<0.001

When comparing all of the treatment groups, we noted a statistically significant group effect in 8 of the 10 most abundant phyla as assessed using a one-factor ANOVA (Table 1; *P* < 0.05). CLR transformed longitudinal depictions of the six bacterial phyla analyzed in the present study in the SCI, antibiotic and diet cohorts can be found in Figs. 3, 4 and 5, respectively. While there were distinct trends among the phyla across time, some changes persisted through the subacute phase after injury. In the acute phase (<14d post-SCI), the *Proteobacteria*, *Tenericutes, Epsilonbacteraeota* and *Cyanobacteria* phyla decreased in abundance compared to controls while *Bacteroidetes, Firmicutes and Spirochaetes* species increased (2-tailed Student t-test; *P* < 0.05). In the sub-acute phase, *Spirochaetes*, *Cyanobacteria and Proteobacteria* remained statistically significantly different relative to controls and only *Proteobacteria* had a greater degree of dissimilarity at the sub-acute stage compared to acute stage, although the difference between post-SCI timepoints was not statistically significant (*P* = 0.576). When comparing the SCI-acute and SCI-subacute groups there was a significant difference noted in 4 of the 10 phyla, which included *Firmicutes, Spirochaetes, Tenericutes and Fibrobacteres*. In only the Spirochaetes phylum was there a significant difference between acute and sub-acute timepoints while both remaining statistically greater than control specimens.

**Table 1:**
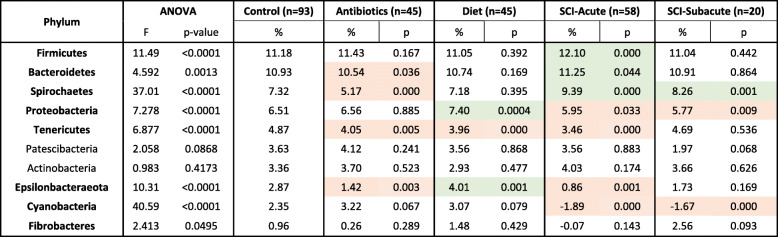
Comparing the relative frequency of the most abundant phyla in the porcine gut microbiome between treatment groups using centered log-ratio transformed data. Global group comparisons for each phylum were first assessed using a one-way ANOVA. Group comparisons were then assessed relative to the control group using an independent student t-test (two-tailed). Shades represent a significantly difference result relative to control group. Red = decrease, Green = increase

We then aimed to assess how the administration of Enrofloxacin (antibiotic group) or our post-surgical diet (diet group) compared to the bacterial fluctuations observed after SCI. We found there to be a number of bacterial phyla in the antibiotic-treated group which demonstrated similar patterns of fluctuation to SCI animals in the acute stage. For instance, relative to controls, the abundance of *Epsilonbacteraeota* and *Tenericute* bacteria decreased in both the antibiotic (2.87 vs 1.42, *P* = 0.003; 4.87 vs 4.05, *P* = 0.005, respectively) and SCI-acute (0.86, *P* = 0.001; 3.46, *P* = 0.0003, respectively) groups. In addition, *Tenericute* bacteria decreased in the diet (4.87 vs 3.96, *P* = 6.2 × 10^− 5^) group relative to controls.

Interestingly, we found several phyla including *Bacteroidetes*, S*pirochaetes*, *Proteobacteria*, *and Epsilonbacteraeota* exhibited different kinetic patterns in the antibiotic and diet cohorts compared to SCI animals. First, the abundance of *Bacteroidete* bacteria decreased to a level just below statistical significance in the antibiotic group (10.93 vs 10.54, *P* = 0.036) and increased in SCI acute samples (11.25, *P* = 0.044). A similar trend was observed in *Spriochaetes* (7.32 vs 5.17, *P* = 0.0002; 9.39, *P* = 5.2 × 10^− 13^). Inversely, in the post-surgical dietary cohort, we found *Proteobacteria* and *Epsilonbacteraeota* bacteria increased in abundance (6.51 vs 7.40, *P* = 0.0004; 2.87 vs 4.01, *P* = 0.001, respectively) whereas these phyla were significantly lower in SCI acute animals (5.95, *P* = 0.033; 0.86, *P* = 0.001, respectively) compared to controls.

These results suggest first that there are unique differences in the microbial composition of animals exposed to traumatic SCI that were not replicated in uninjured animals exposed to the same diet or antibiotic intervention. Second, we observed that SCI induces a time-dependent effect on intestinal microbiota, largely confined to the first 2 weeks post-SCI. It should be noted that two of the observed fluctuations in the SCI cohort may be partially explained by the administration of Enrofloxacin or the post-surgical diet (i.e. a consequence of the experimental procedure), while others may be exclusive to the SCI itself.

**Fig. 6:**
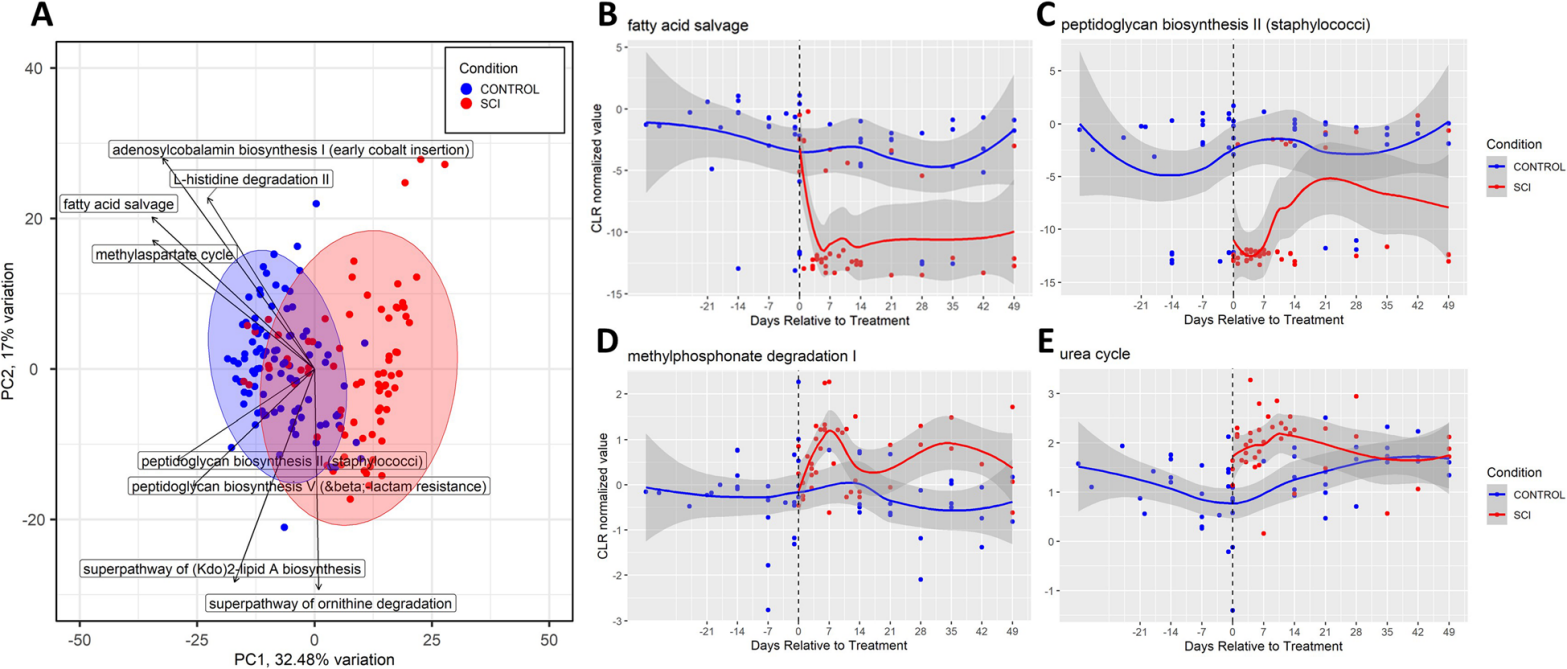
Alpha diversity (species evenness). Species evenness is calculated by dividing the entropy (Shannon index) by the logarithm of the number of ASVs. A value of 1 on the evenness index indicates a perfectly even (equal abundance) sample, whereas small values indicated a skewed distribution. Groups are compared using Kruskal-Wallis analysis. *p<0.05; **p<0.01; ***p<0.001

To further understand how the abundance of various gut microbes can affect the host from a functional perspective, we performed a functional inference analysis using PICRUSt2 (Fig. 6). First, the PICRUSt2 tool generated functional classifications of 362 different pathways and parameters. Differential abundance of microbes was calculated (ALDEx2) and Wilcoxon Rank Sum test statistics were computed using SCI and Control as groups of interest. We found a statistically significant difference (*P* < 0.05) in 133 of 362 parameters examined. In Fig. 6A, a principal component analysis (PCA) was performed to determine which characteristics influence the principal component and a biplot was used to add a loadings plot to examine how strongly those characteristic influence the principal component. Using this, along with the effect size estimation with PICRUSt we isolated the parameters and pathway which best explained the variance between gut microbes in control and SCI pigs. We found that the greatest negative effect was seen in bacteria involved in the methylaspartate cycle (*P* = 5.9 × 10^− 12^), fatty acid salvaging (Fig. 6B. *P* = 5.4 × 10^− 12^) and peptidoglycan biosynthesis (Fig. 6C. *P* = 8.5 × 10^− 10^) whereas the greatest positive effect was seen in bacteria responsible for methlyphosphonante degradation (Fig. 6D. *P* = 1.7 × 10^− 8^), the urea cycle (Fig. 6E. *P* = 1.1 × 10^− 7^) and NAD salvaging (*P* = 1.3 × 10^− 6^).

**Fig. 7:**
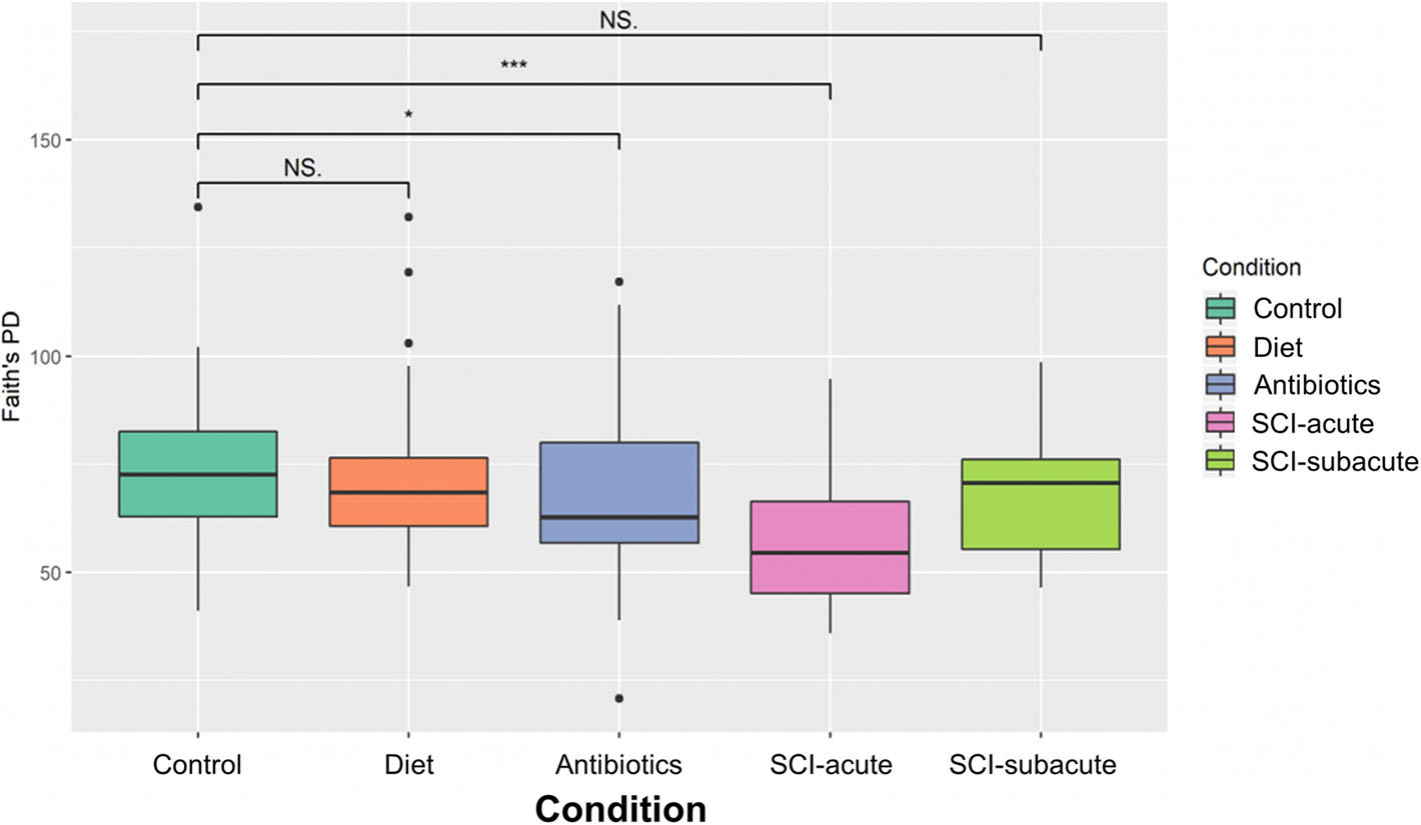
Volatility charts quantify the temporal stability or volatility of the microbiome between treatment groups. Herein we’ve selected the Shannon diversity index as the metric to assess volatility on the y-axis. Categorical sample metadata was grouped by treatment received and combined for averages at each timepoint. Blue=SCI; Orange=Antibiotics; Green=Diet; Red=Control

Another parameter used to describe the bacteria present in the microbiome and their relative differences between treatment groups are alpha diversity metrics. Alpha diversity is a local measure that refers to the average species diversity in an ecosystem or specific area such as the gut. We analyzed both the abundance of species (richness) and the distribution of these bacteria (evenness) in each of our samples. As bacteria are identified using amplicon sequence variants (ASVs), an increase in ASVs reflects an increase in the richness of bacteria within an ecosystem whereas evenness refers to how equally abundant species are in the environment. Globally, increases in bacterial species richness and evenness are markers of a healthy gut microbiome [21], although this is still contested today [22]. Comparing the richness of each sample across our different treatment groups (Fig. 7) revealed that, relative to control samples, there was a significant decrease in species richness in the antibiotic control group (*P* < 0.05) as well as the SCI acute group (*P* < 0.001). In contrast, we did not observe a significant decrease in species richness comparing our dietary group or SCI subacute group to control samples.

**Fig. 8:**
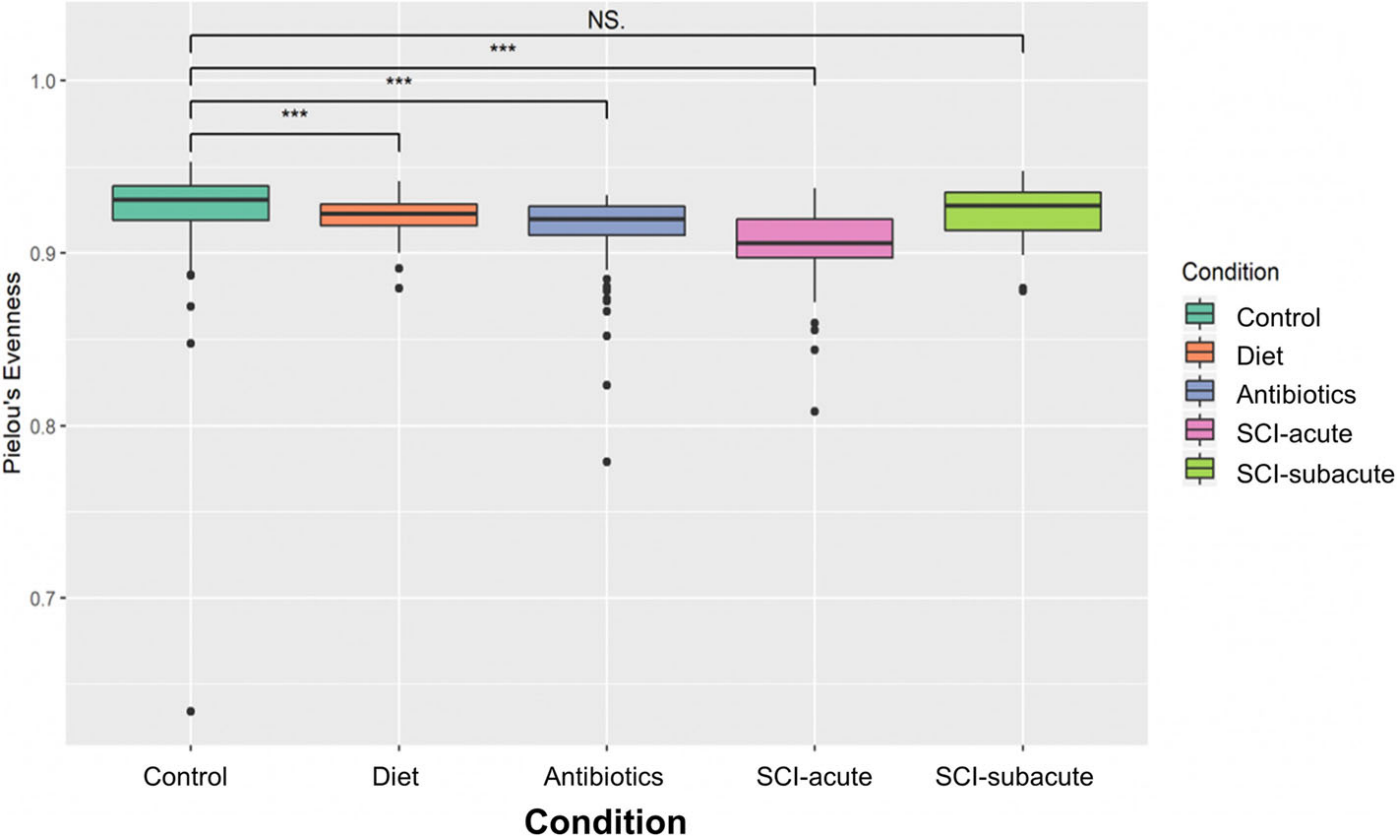
Schematic of experimental design and sampling overview. I) Non-SCI control group (*n*=9); these animals received no intervention over the course of 3-7weeks; II) Antibiotic group (*n*=3); these animals received oral Enrofloxacin treatment orally at 10mg/kg per day for 5 days; III) Diet group ((*n*=3); these animals consumed the standard post-surgical diet for 5 days which consisted of 150g Mazuri youth pellets and 250g of Pedigree wet food before returning to their normal control diet (NCD) after 5 days; IV) SCI group ((*n*=8) These animals underwent contusion/ compression SCI surgery along with IV Enrofloxacin (5mg/kg) and the standard postsurgical diet. Each point refers to a fecal sample collected from a given animal. Timeline represents weeks relative to treatment

We also analyzed species evenness and quantified how equal the community is in different sample groups (Fig. 8). By way of example, if an ecosystem contains 40 foxes and 1000 dogs, the community is considered not ‘even’. We found a significant decrease in species evenness in the antibiotic (*P* < 0.001), diet (*P* < 0.001) and SCI-acute (*P* < 0.001) groups. No statistically significant difference was noted in the SCI subacute group relative to control values.

**Fig. 9:**
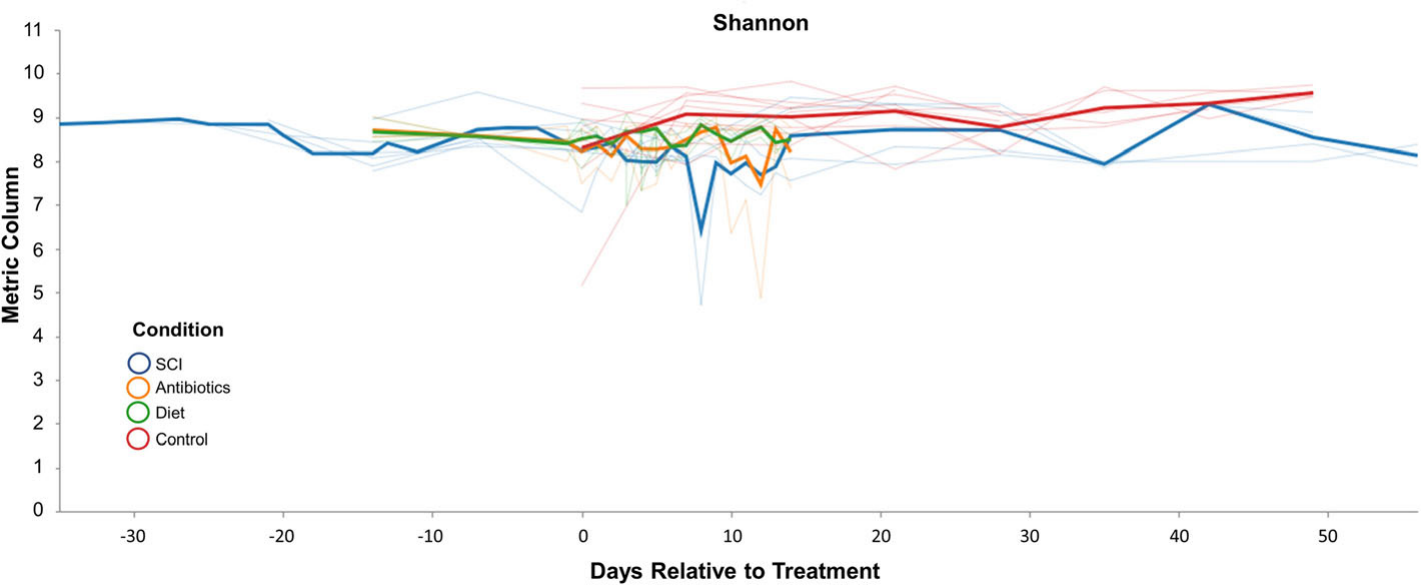
Phylum level taxonomy of fecal bacterial populations from Yucatan minipigs in the control group exclusively (*n*=93). Relative abundance of amplicon sequence variants at the phylum level. Each color indicates one phylum. Column height represents the relative abundance of reads (%) based on 16S rRNA sequencing

As we collected samples weekly, we were able to assess the stability of the microbiome over time both within and between subjects. Using volatility control charts in QIIME2, we plotted the stability of the microbiome longitudinally in our various treatment groups (Fig. 9). The temporal stability or volatility of a metric between individual subjects or groups of subjects can be an important measurement, indicating periods of disruption, disease, or abnormal events. Microbial volatility, the variance in microbial abundance, diversity, or other metrics over time, can be a marker of ecosystem disturbance, disease or abnormal events [23–25] and provides another important metric for comparison between experimental groups. Using the Shannon diversity index [26], a higher degree of variability or “volatility” between samples would result in a lower value on the index, whereas more stability between and within samples would result in a higher Shannon score. In the control group, we noted the greatest degree of volatility when the animals initially arrived at the treatment facility (Fig. 9). Over time, we found the microbiome became more stable in our control animals. When assessing the stability (or volatility) of the microbiome before and after SCI, we noted the gut ecosystem to be most volatile (least stable) within the first ~ 10 days after injury (Fig. 9) and to rebound to baseline levels shortly thereafter. This trend is similar to the observations noted in the relative abundance of the dominant bacterial phyla and was also noted in non-SCI animals receiving Enrofloxacin treatment. No significant change in volatility was found in the dietary cohort.

## Discussion

This study presents a longitudinal characterization of the Yucatan pig gut microflora before and after traumatic SCI. There were three main aims of this study. First, we sought to describe the intestinal microbiome of Yucatan pigs and determine its composition as well as its stability over time at the phylum level. Second, we examined how SCI changed this baseline microbiome composition in a time-dependent manner, from 1 day to 7 weeks post-injury. Third, we sought to distinguish which changes in microbiome composition could be attributable to the antibiotics or dietary alterations that are associated with the experimental SCI. To address these research objectives, we used our established porcine model of thoracic contusive SCI [19, 20] and examined the alterations to the bacterial ecosystem over time.

In summary, we determined that the microbiome consists largely of bacteria belonging to the *Bacteroidetes* and *Firmicutes* phyla (~ 90%), with a smaller fraction comprising *Spirochaetes* (~ 5%) *and Proteobacteria* (~ 4%). We found that the most significant alterations to the gut microbiome occur within the first 14 days post-SCI, which we have described as a “SCI-acute” window. Further, we have identified specific phyla, (eg. *Spirochaetes)*, which demonstrate a unique response to SCI surgery which was not observed in the non-SCI group treated with antibiotics or in the standard post-surgery diet.

Our relatively large dataset of 93 Control samples collected from the feces of 23 pigs gave us a solid foundation to examine and interpret both the concentration of various bacterial phyla at each timepoint, as well as how they behave longitudinally. We found that the most dominant phyla in the gut of Yucatan minipigs, making up almost 98% of all species detected, include *Bacteroidetes, Firmicutes, Spirochaetes, Tenericutes, Proteobacteria* and *Actinobacteria*. At this level of classification (phylum), the fraction of each bacterial phyla present in the gut largely resembles humans and other mammalian models [18, 27–33], as well as murine intestinal bacteria [12, 28]. However, although some of the gut microbes present in murine species are shared with the human and pig microbiome, Ley et al. (2005) demonstrated that almost 85% of the subgenera present in the mouse gut are not present in humans. We therefore aim to expand upon this present analysis in subsequent work to include more specific Families, Genus and Species when comparing the Yucatan gut to human beings in order to better assess its translational potential.

Longitudinally, we found that in the uninjured “normal” pigs the concentrations of the most dominant phyla remain relatively consistent over time with only minor fluctuations. Further, based on Volatility Control Analyses (Fig. 8), we observed that the microbial composition of Yucatan minipigs remained relatively stable over the course of 5–7 weeks. We also noted the volatility decreased slightly as the animals adapted to their new environments (as represented by an increase in the volatility index over time). This was exemplified in particular by two animals who, upon arrival at our animal care facility, had a very high proportion of Proteobacteria species (as high as 70% of all bacteria present in the gut) but these values returned to concentrations which better resembled values taken from other subject after a few days. The temporal stability noted in control animals was reassuring to then evaluate an intervention (such as a spinal cord injury) and determine the effect on the fairly stable microbial ecosystem. We acknowledge that “microbial stability” is a challenging metric to assess as there are often considerable fluctuations between and within individuals over time [34, 35], further microbiome volatility is a poorly understood topic today. Initially, the volatility of the gut microbiome was described as the degree of change between timepoints [36, 37] and aside from this, volatility has been scarcely discussed in the microbiome field. Recently, its been shown that increased volatility is linked to an increased stress response in two cohorts of mice and one cohort of humans [38]. The extent to which microbial volatility can influence the severity of neurological injury and recovery or visa versa has yet to be investigated but could shed light on this phenomenon.

*Bacteroidetes* phylum is an abundant group of aerobic and anaerobic, rod-shaped, Gram-negative bacteria which can be found throughout the intestinal tract. *Bacteroidetes* are known for their ability to digest carbohydrates such as complex oligoglycans found in mucin [39]. The degradation of these carbohydrates results in the production of short chain fatty acids (SCFAs) such as butyrate, propionate and acetate which are subsequently reabsorbed by the host for energy. In a study examining the gut microbiome of chronic quadriplegic SCI patients, Zhang et al. (2018) found that *Bacteroidetes* species was at a significantly lower concentration relative to uninjured, healthy male participants [40]. Similarly, Gungor et al. (2016) showed a decrease in *Bacteroidetes* species in the chronic phase of SCI patients with upper and lower motor neuron bowel disease [41]. In contrast, increases in *Bacteroidetes* species were noted in murine SCI studies in the acute and subacute stages [12, 42]. Here we found that the relative abundance of *Bacteroidetes* bacteria increased to a level below our significance threshold during the acute post-injury stage in Yucatan pigs, then returned to baseline values in the SCI-subacute stage. Inversely, decreases in Bacteroidetes species were noted in non-SCI animals given our standard post-surgical antibiotics and our diet cohort, although the decrease in Bacteroidetes in uninjured animals fed the standard post-surgical diet was not statistically significant relative to controls. These findings suggest that this bacterial shift may be unique to the SCI surgery and/or the agents that were administered in the acute setting.

*Firmicutes* are generally classified as endospore forming, obligate and facultative anaerobes [43]. This phylum contains many commensal bacterial species such as *Ruminoccocus* which, like many bacteria within the *Bacteroidetes* phylum, contribute to digestion by fermenting high-fiber carbohydrates and producing butyrate as a by-product. Butyrate has been shown to affect enteric neurons and can exert potent anti-inflammatory effects on microglia in the CNS [44–48]. A study examining the gut microbiome in chronic SCI patients found that the concentration of butyrate-producing bacteria, all of which fall into the Firmicute phylum, were consistently lower in chronic SCI patients with upper and lower motor neuron bowel disease approximately 20 months after SCI [41]. A 2018 study in human SCI patients 6 months or more after their respective injuries, showed that *Megamonas* species (*Firmicute*) was significantly decreased relative to healthy participants [40]. A contrasting effect was found in rodent models of SCI in which a statistically significant increase in *Clostridiales* (*Firmicute*) bacteria was demonstrated 2 weeks after SCI which remained significantly greater for up to 4 weeks post-injury [49]. O’Connor et al. also showed a statistically significant increase in 3 bacterial species in rats following contusive SCI, two of which belong to the *Firmicute* phylum, 8 weeks after SCI. It must also be noted that the rodents in the aforementioned study were given antibiotics (gentamicin, 5 mg/kg) for the first 7 days after injury [42]. The results of the present analysis were more similar to acute SCI studies performed on rodents such that we found Firmicute bacteria to proliferate in the acute setting after SCI. A similar increase in *Firmicute* species was noted in one of our non-SCI animals treated with Enrofloxacin, thus making it difficult to conclude that the fluctuation observed in Firmicute bacteria was related to SCI surgery or merely antibiotics administration. However, we must also consider that antibiotics can induce lasting changes to the gut microbiome that may not detected within the timeframe of the present study [50–53]. Further it must also be considered that different antibiotics can have different effects on the gut microbiome due to their distinct pharmacokinetics concerning hepatic or renal elimination (reviewed in *Kim* et al. *2017)*. Interestingly, Kigerl et al. 2016 showed that SCI induced gut dysbiosis in animals who did not receive antibiotic treatment. This highlights one of the major challenges of conducting a study to evaluate the changes in the microbiome after experimental SCI, where the inherent conditions of the experiment itself may influence the microbiome.

*Spirochaetes* are anaerobic bacteria with a distinctive spiral-shape body composition which allows them to twist and move about. Many species within the *Spirochaete* phylum are known to cause diseases such as Lyme disease (*B. burgdorferi)*, syphilis (*T. pallidium*) and leptospirosis (*Leptospira*). Interestingly, these diseases can often result in progressive neurological decline induced by severe neural atrophy [54, 55]. There is evidence of an increase in abundance of *Spirochaetes* in patients suffering from Alzheimer’s disease (AD) relative to healthy controls [56]. Experimentally, when neuronal and glial cells were exposed to *B. burgdorferi* extracted from the brains of AD patients, there was accumulation of AB-immunoreactive “plaques” and neurofibrillary tangles, a hallmark of AD progression [57].

The increase in *Spirochaetes* in the SCI acute and subacute groups is noteworthy in light of the decrease in the non-SCI animals that also received the post-surgical antibiotic regimen. Because this increase in the Spirochaetes bacteria is unique to SCI-treated animals, it would be interesting to assess how their relative abundance might be correlated with recovery following SCI, and whether this specific phylum would be a possible target for future therapeutic intervention.

Diet is perhaps the single most important determinant of the gut microbial composition throughout one’s life [58, 59]. Interestingly, the administration of our standard surgical diet had a minimal impact on the composition of the gut microbiome, with no statistically significant difference between pre- and post-dietary samples in most of the analyzed bacterial phyla, with the exception of *Proteobacteria* and *Tenericutes*. Studies have shown that the consumption of high-fat, low-fiber diets can result in increased levels of Proteobacteria relative to low-fat high-fiber diets, as seen in European children [60]. Furthermore, the consumption of artificial sweeteners and emulsifiers (commonly used as additives in processed foods), has also been shown to favour Proteobacteria [61, 62]. Therefore, it is possible that increasing the volume of wet dog food nourished various *Proteobacteria* species in the gut resulting in increased detection during next-generation rRNA sequencing.

### Bacterial fluctuation as time-dependent phenomenon

This is the first longitudinal SCI study in a large animal model to compare and contrast the impact on the microbial ecosystem at acute and subacute phases of traumatic SCI. Clearly, the greatest degree of bacterial fluctuation and α-diversity in Yucatan pigs occurs within the acute window from 0 to 14 days post-SCI. The time-dependent nature of these results differs from those presented in Kigerl et al. 2016 such that more drastic changes are noted from 14 days post-injury onward in their study and there was no statistically significant change in *Bacteroidales* and *Clostridales* concentrations in the first week after injury. Our longitudinal results show a different kinetic response to SCI surgery and antibiotic treatment such that the most dramatic change in microbial composition is noted within the first 2 weeks after treatment.

A major unanswered question from our data is whether or not the temporary shift in microbial composition is consequential to the recovery post-SCI. Kigerl et al. 2016 showed that inducing dysbiosis via antibiotics pre-SCI exacerbated injury severity resulting in worsened pathological outcomes and diminished locomotor performance in mice; additionally, the authors demonstrated that post-injury treatment using probiotics could improve functional outcome and significantly decrease lesion extent compared to control subjects. The extent to which pre or post-injury dysbiosis influences recovery and pathological outcomes has yet to be investigated in a large animal model but could be a key step to finding therapeutic targets for future treatment and translating those findings to clinical practice.

Gungor et al. 2016 examined microbiome dynamics over time and showed that chronic SCI patients (~ 20–100 months after injury) have lower levels of *Firmicute* bacteria along with higher levels of *Bacteroidetes*, which is different than the SCI-induced changes we observed in the present study and in rodent models of SCI [42, 49]. It is possible that the initial shift in microbiome composition is more reflective of injury, immune response, anesthesia, diet, etc., whereas chronic fluctuations come as a result of GI tract dysfunction such as delayed gastric emptying, impaired motility, decreased mucin production and impaired immune function. Interestingly, it has been demonstrated that post-SCI dysbiosis results in a loss of SCFA producing bacteria (many belonging to the Firmicute phylum) and may contribute to microglia-mediated neurotoxicity after injury and influence long-term recovery [41, 63–65]. In the present study, we found the concentration of fatty acid salvaging bacteria decreased significantly after SCI (Fig. 5A,B) and remained well below control samples beyond the SCI-acute window. These findings suggest that these anti-inflammatory metabolites such as butyrate, propionate and acetate, may be depleted after SCI. In rodents, Kigerl et al. 2016 showed that the administration VSL#3, a medical-grade probiotic consisting of several SCFA-producing bacteria, decreased the severity of injury and improved locomotor outcomes after a 75-kilodyne spinal contusion at the T9 level. It would therefore be beneficial to investigate the therapeutic potential of pre- or probiotics which target these species in a large animal model.

This highlights the gap in our understanding of the impact of SCI on the microbiome when comparing the pre-clinical and clinical studies. Those performed in murine models examine the acute and subacute phases of injury and generally occur < 4–8 weeks post-SCI [42, 49, 66]. In contrast, human studies have to date been largely confined to more chronic SCI patients [33, 40, 41] although we are aware of efforts to characterize the microbiome in acutely injured patients. In order to address this issue, studies need to be conducted longitudinally in acute SCI patients within the first week of their injury with prospective assessment of functional outcomes with microbial composition to correlate specific bacterial groups with outcome measures such as sensorimotor recovery, or neuropathic pain. It may be possible to utilize the microbiome as a predictive biomarker for recovery from neurological impairment similar to the Stroke Dysbiosis Index [67]. It is, of course, acknowledged that individuals who suffer a spinal cord injury are subjected to a plethora of other “physiologic perturbations” that may influence their microbiome such as enteral feeds, surgical procedures, antibiotics, and a myriad of other medications. Similar to our experiments, these issues will undoubtedly cloud the interpretation of microbiome changes that occur as the direct result of the neurologic injury. Second, we must consider extending animal studies to more chronic stages to examine how the long-term GI tract impairments and neurological recovery influence the microbiome and visa versa.

### Limitations

It is worth noting the limitations of the present study. First, we acknowledge the absence of a non-SCI treatment group which received both Enrofloxacin as well as the post-surgical dietary regiment. This cohort of animals would provide us with a more representative depiction of the microbial composition post-SCI. Second, we acknowledge the fact that we did not perform a sham SCI surgery to best imitate pre/post-surgical SCI conditions. This would ultimately be the most representative account of the microbiome changes that occur in a non-SCI animal receiving all the other experimental/surgical conditions as the SCI animals. Such conditions include not just antibiotics and dietary changes but also anesthesia, pain medications, stress response, etc. While the costs and time requirements for such a study are beyond the scope of this work, we suggest that our dataset will serve as an important benchmark and resource for future work. In addition, we believe it should be mentioned that the contusion/compression model of thoracic SCI carries several intrinsic limitations. For instance, we acknowledge that the compression and contusion injury in human patients is normally caused by structures surrounding the spinal cord such as the intervertebral discs, vertebral bone, ligaments, epidural components, articular processes and capsules, etc. and these different anatomical structures are not only compressing/contusing the spinal cord but also inducing important inflammation which would affect the general autonomic afferents/efferents differently with unknown implication in the gut microbiome. Further, we acknowledge that the antibiotic group was given oral as opposed to IV antibiotics and although the dose of antibiotics was adjusted to account for the route of drug administration, it is recognized that different methods of drug delivery can have different effects on the gut microbiome [50, 51, 53, 68]. In addition, medications used to treat SCI animals in the present study such as Fentanyl and Metaclopramide can influence digestion by decreasing and increasing gastric motility, respectively. Therefore we acknowledge that these agents can certainly influence the composition of the gut microbiome and further, the duration of given administration can also affect such outcomes. We encourage other studies to pursue this investigation as the results will no doubt uncover some interesting implications of various treatments after injury.

We also acknowledge the relatively small group size (*n* = 3) for the diet and antibiotic treated animals, which makes the interpretation of the variability observed in the microbiome changes difficult. Finally, the authors acknowledge the variation of the SCI localization, degree of contusion and duration of compression differ between animals and this produces different levels of dysfunction and is therefore a possible source of microbial variation.

## Conclusions

The data presented in this study provides a better understanding on the microbial response to SCI in the porcine microbiome. Further, we found specific bacterial phyla whose kinetic responses were unique to SCI animals and were not seen in non-SCI minipigs who received the same post-surgery diet or antibiotic regiment. We believe this information will be critical for further microbial studies involving neurological insults and could also aid in the design and development of bacterial-based therapeutic interventions post-SCI.

## Methods

All animal experiments were performed in accordance with the guidelines of the Canadian Council for Animal Care, carried out in compliance with the ARRIVE guidelines and approved by the University of British Colombia’s Animal Care Committee (A16–0311 SCI in Pigs).

### Animals and experimental design

Female Yucatan pigs (*n* = 23, purchased from either S&S Farms, CA, USA, or Sinclair Bio-resources, Columbia, MO) weighing 20-30 kg were group-housed at our large animal facility. For information regarding housing, husbandry and environmental enrichment please see previous publications [19, 20]. Upon arrival, all animals were introduced to a 3:1 mixture of pellets (300 g, Mazuri) and 100 g wet dog food (Pedigree, Meaty Loaf)(referred to as “standard diet”) twice daily with ad-libitum access to water. Animals were kept in a separate holding area for 14 days to quarantine before the initiation of any experimental procedure.

We had a number of objectives in this research study. First, we determined the bacterial composition of the gut microbiome in the Yucatan minipig over time in the normal uninjured state as this is, to the best of our knowledge, the first study to do so. Second, we looked to determine the effect of administering an oral antibiotic commonly used as prophylaxis following experimental surgery. Third, we determined the effect of altering the diet to our standard post-surgical diet on the microbiome. Lastly, we sought to determine the effect of sustaining a severe thoracic SCI on the gut microbiome.

In order to answer these research questions, we divided our animals into four treatment groups: Control (*n* = 9), SCI (*n* = 8), Diet (*n* = 3) and Antibiotics (*n* = 3). A schematic of the experimental conditions is show in Fig. 1.

#### Control group

A group of uninjured “Control” animals (*n* = 9) were fed the standard diet and did not receive antibiotics throughout the study’s duration (3–7 weeks). All animals were administered their respective diets twice daily, first in the morning (0700–0800 am) and then in the evening (1600–1700 pm). It is worth noting that all samples collected before a given treatment were also considered Control specimens.

#### Antibiotics group

Animals in the “Antibiotics” group (*n* = 3), were fed the standard diet and received oral Enrofloxacin (Baytril 10 mg/kg) antibiotic tablets for 5 days. These animals remained untreated thereafter to assess the effect of post-surgical antibiotics. Enrofloxacin is a fluoroquinolone which is efficacious against a variety of bacterial pathogens in different animal species and is commonly used to treat respiratory and gastrointestinal tract infections caused by gram-negative bacteria. Nielsen & Gyrd-Hansen (1997), demonstrated that a therapeutically active concentration of Enrofloxacin could be achieved for at least 24 h in pigs at an oral dose of 10 mg/kg and an IV dose of 5 mg/kg. Therefore, in order for us to best mimic the antibiotic dose (IV 5 mg/kg daily for 5 days) given to our minipigs after injury, and without the ability to administer IV antibiotics to intact minipigs for ethical and practical reasons, we delivered enrofloxacin orally at a dose of 10 mg/kg for 5 days.

#### Diet group

In the “Diet” group, we assessed the impact of the post-surgery diet on gut microflora. These *n* = 3 uninjured animals were fed the post-surgery diet for 5 days, before returning to the standard diet (9 days). The “Diet” consists of a 1:1.5 mixture of pellets (150 g, Mazuri) and wet dog food (225 g, Pedigree, Meaty Loaf)(referred to as “post-surgery diet”) for 5–7 days. The ratio of wet dog food to pellets is modified after surgery as wet dog food is easier for the animals to chew and digest. Animals in the Diet group were housed in separate holding areas for the duration of their study (14 days).

#### SCI group

SCI animals (*n* = 8) were subjected to a contusion/compression injury consisting of a 50 g weight drop at either the T2 or T10 level, followed by sustained compression, described in more detail below: *Porcine Model of Thoracic SCI*. All SCI animals received antibiotic treatment (Enrofloxacin (Baytril), intravenous (IV), 5 mg/kg) for the first 5–7 days after surgery along with the standard post-surgical diet as described above.

### Porcine model of thoracic SCI

Surgical procedures for spinal cord injury (SCI) and post-operative care were performed as previously described [20, 69, 70]. Animals (*n* = 8) were pre-anesthetized with an intramuscular (IM) injection of Telazol (4–6 mg/kg), Xylazine (1 mg/kg), and atropine (0.02 mg/kg). Animals were endotracheally intubated, and mechanically ventilated at ~ 15/breaths/min. General anesthesia was maintained with either a gas mixture of O_2_ (0.6%) and N_2_ (1.4%) and Isoflurane at 0.5–5% concentration or a mixture of Propofol (6–12 mg/ml), Fentanyl (8–14 mcg/kg), and Ketamine (5–12 mg/kg). Midazolam was given (0.2 mg/kg/hr., IV) to 4 of 8 animals.

The affected levels of the thoracic spine were exposed through a longitudinal midline incision. The spinous processes, laminae, and transverse processes were exposed two levels above and three levels below the impact site (eg. T8-T13 for T10 SCI). A total of 4x pedicles screws (Select™ Multi Axial Screw, Medtronic, Minneapolis, MN) were placed bilaterally in the pedicles of the spine. After the laminectomy was performed, the guide rail of the impactor was rigidly secured to the pedicle screws by two rods on both sides and aligned vertically using spirit levels. Immediately prior to the injury, the animal’s ventilation was stopped to cease respiration motion and the trigger pin was removed to induce the injury, after which ventilation was resumed. All drop heights had an additional 100 g static weight placed to simulate sustained compression.

As these animals were used in studies to answer different research questions [71–74] they were subjected to different injury levels (T2 or T10), drop heights (20 or 50 cm) and compression times (5, 30, or 120 min). The injury and impact parameters for each animal can be found in Supplemental Table 1.

After the surgery, a single injection of maropitant citrate (Cerenia; 1 mg/kg s.c.) was given to limit opioid-induced motion sickness and vomiting. Metoclopramide (0.5 mg/kg; 2-3 days) was administered to 6 out of the 8 SCI animals as needed to assist gastric emptying. All SCI animals and were maintained on a continuous rate infusion of fentanyl for pain control, which the animals were weaned off over the course of 3–4 days. This required close observation and could be adjusted several times a day if necessary.

Further, all procedures described in this study have been discussed in length during prior consultations with licensed on-site veterinarians. Our veterinarians continue to educate themselves on current techniques of anesthesia, surgery and analgesia (workshops and conferences, consultation with acknowledged experts in the field of research) and will utilize and teach new techniques as they arise to improve both the surgical and anesthetic methods used. In addition, refinements to prevent/minimize pain and discomfort was implemented through the use of aseptic surgical techniques performed by experienced surgeons. Anesthesia was be administered and carefully monitored throughout the procedure by trained animal care technicians.

### Fecal sample collection

Fecal sampling date, time, and description were logged for all sampling timepoints. Feces were generally collected fresh in the morning directly from the pen and a sample from the interior of the feces was immediately transferred into an RNase-free 1.5 mL Eppendorf tube, using a stainless-steel rod which was pre-sterilized with 70% ethanol (EtOH). All samples were labelled, and stored in a − 70 °C freezer for cryopreservation until further processing. All materials were sterilized with 70% EtOH between each use.

Fecal samples were collected on pre-determined days before and after their respective treatment (Fig. 9). A total of 262 fecal samples were analyzed using 16S rRNA gene sequencing, (described in detail in the paragraph below: *DNA extraction and 16S rRNA gene sequencing*). It is worth noting that animals in the present study participated in other ongoing research projects and were therefore euthanized at the conclusion of those studies according to their experimental timeline.

### DNA extraction and 16S rRNA gene sequencing

DNA was extracted using the MagAttract PowerSoil DNA KF Kit, according to standard protocol (Qiagen; Hilden, Germany). DNA was visualized on an agarose gel and quantified using Qubit fluorometry, according to manufacturer’s instructions (ThermoFisher; Waltham, MA). The v3 and V4 regions of the 16S rRNA gene were PCR amplified using primers F: 5′-TCGTCGGCAGCGTCAGATGTGTATAAGAGACAGCCTACGGGNGGCWGCAG and R: 5′- GTCTCGTGGGCTCGGAGATGTGTATAAGAGACAGGACTACHVGGGTATCTAATCC using 12.5 ng input DNA per sample. These amplicons were then converted to sequencing libraries using an 8-cycle indexing PCR with Nextera XT primers (Illumina; San Diego, CA). Libraries were cleaned using Ampure XP beads, according to manufacturer’s instructions (Beckman Coulter; Pasadena, CA) and QC’d using Agilent (Santa Clara, CA) Bioanalyzer and Qubit fluorometry. Libraries were then pooled and sequenced over two MiSeq v3 flow cells (Illumina) to generate paired-end 300 bp reads. Raw data was processed using bcl2fast v2.20.0.422 to generate demultiplexed fastq files.

### Sequencing data processing and analysis

Illumina sequencing data from each experiment were processed and analyzed using QIIME2 (v2019.7.0). Sequencing analyses were performed by blinded specialists. In brief, paired reads were trimmed to remove low-quality bases (Q < 20), adapter, and primer sequences using the Cutadapt module within QIIME2. Resultant reads were denoised and merged using DADA2. The reads were assigned to species-equivalent amplicon sequence variants (ASVs) at 99% similarity by QIIME2 (phylogeny align-to-tree-mafft-fasttree) using the feature-classifier classify-sklearn algorithm against the Silva_132 release reference sequences (https://www.arb-silva.de/documentation/release-132). As datasets collected via 16S rRNA sequencing are considered “compositional” due to an arbitrary total produced by the sequencing instrument (Gloor G et al. 2017), a centered log-ratio (CLR) transformation was performed on all time series plots to analyze the relative abundance of each phylum in the present study. Stacked barplots and alpha diversity metrics were not transformed. Principal component analysis (PCA) was performed on CLR values generated by ALDEx2 (medians of each distribution were calculated from these values). PICRUSt2 was used to generate functional predictions, using default parameters. Differential abundance of microbes and functional classifications were calculated (ALDEx2). Alpha diversity metrics (species richness, species evenness and volatility charts) were analyzed using QIIME2. Plots were generated in R (version 4.0.5) using either ggplot2 or PCATools (version 2.3.13, https://bioconductor.org/packages/release/bioc/html/PCAtools.html).

### Bioinformatics and statistical analysis

Differences in CLR values between treatment groups at the phylum level were assessed using a one-way ANOVA via PRISM Graphpad software (version 8.2.1). Statistical hypothesis testing (Graphpad) was used to correct for multiples comparisons. Group comparisons were then assessed relative to the control group using an independent student t-test (two-tailed). Unless otherwise specified, we use the term “microbiome” to refer to the bacterial gut microbiome. Wilcoxon Rank Sum test and Welch’s t-test statistics were computed using SCI and Control as groups of interest. Data was subsetted based on a significant *p*-values of < 0.05 in all cases, using a Benjamini-Hochberg (BH) post-hoc correction. Comparisons between alpha diversity metrics (species richness and evenness) were assessed using Kruskal-Wallis analysis (QIIME2). All tests of significance were two-sided and significance was set at *p* < 0.05.

## Supplementary Information

**Additional file 1.**

**Additional file 2.**

**Additional file 3.**

## Data Availability

The dataset supporting the conclusions of this article is available in the Open Data Commons for Spinal Cord Injury repository, “Kwon Lab-16S rRNA Sequenced Microbiome Data” in *https://scicrunch.org/odc-sci/lab/dataset?labid=103&datasetid=568*. To access, please sign up at: https://scicrunch.org/odc-sci/join, and request to access data.

## Abbreviations

AD: Alzheimers disease
ASV: Amplicon sequence variants
CLR: Centered log-ratio
CNS: Central nervous system
GI: Gastrointestinal
IV: Intravenous
QIIME: Quantitative insights into microbial ecology
SCFA: Short chain fatty acid
SCI: Spinal cord injury
T2: 2nd thoracic vertebrae
T10: 10th thoracic vertebrae

## References

[1] Round JL, Mazmanian SK (2009). The gut microbiota shapes intestinal immune responses during health and disease. Nat Rev Immunol.

[2] Hooper LV, Littman DR, Macpherson AJ. Interactions between the microbiota and the immune system. Science (80- ). 2012;336(6086):1268–73. 10.1126/science.1223490. 10.1126/science.1223490 PMC4420145 22674334

[3] Nicholson JK, Holmes E, Kinross J, Burcelin R, Gibson G, Jia W, et al. Host-gut microbiota metabolic tnteractions. Science. 2012;336(6086):1262–8. 10.1126/science.1223813. 10.1126/science.1223813 22674330

[4] Martin CR, Osadchiy V, Kalani A, Mayer EA. The brain-gut-microbiome axis. Cell Mol Gastroenterol Hepatol. 2018;6(2):133–48. 10.1016/j.jcmgh.2018.04.003. 10.1016/j.jcmgh.2018.04.003 PMC6047317 30023410

[5] Goodrich JK, Waters JL, Poole AC, Sutter JL, Koren O, Blekhman R, et al. Article human genetics shape the gut microbiome. Cell. 2014;159(4):789–99. 10.1016/j.cell.2014.09.053. 10.1016/j.cell.2014.09.053 PMC4255478 25417156

[6] Tillisch K. The effects of gut microbiota on CNS function in humans. Gut Microbes. 2014;0976(3):404–10. 10.4161/gmic.29232. 10.4161/gmic.29232 PMC4153780 24838095

[7] Yano JM, Yu K, Donaldson GP, Shastri GG, Ann P, Ma L, et al. Indigenous bacteria from the gut microbiota regulate host serotonin biosynthesis. Cell. 2015;161(2):264–76.10.1016/j.cell.2015.02.047. 10.1016/j.cell.2015.02.047 PMC4393509 25860609

[8] Samuel BS, Shaito A, Motoike T, Rey FE, Backhed F, Manchester JK, Hammer RE, Williams SC, Crowley J, Yanagisawa M, Gordon JI (2008). Effects of the gut microbiota on host adiposity are modulated by the short-chain fatty-acid binding G protein-coupled receptor, Gpr41. Proc Natl Acad Sci U S A.

[9] Haghikia A, Jörg S, Duscha A, Berg J, Manzel A, Waschbisch A, Hammer A, Lee DH, May C, Wilck N, Balogh A, Ostermann AI, Schebb NH, Akkad DA, Grohme DA, Kleinewietfeld M, Kempa S, Thöne J, Demir S, Müller DN, Gold R, Linker RA (2015). Dietary fatty acids directly impact central nervous system autoimmunity via the small intestine. Immunity..

[10] GBD 2016 Traumatic Brain Injury and Spinal Cord Injury Collaborators. Global, regional, and national burden of traumatic brain injury and spinal cord injury, 1990–2016: a systematic analysis for the Global Burden of Disease Study 2016. Lancet Neurol. 2019;18(1): 57–87. 10.1016/S1474-4422(18)30415-0. 10.1016/S1474-4422(18)30415-0 PMC6291456 30497965

[11] Kigerl KA, Mostacada K, Popovich PG (2018). Gut microbiota are disease-modifying factors after traumatic spinal Cord Injury. Neurotherapeutics..

[12] Kigerl KA, Hall JCE, Wang L, Mo X, Yu Z, Popovich PG (2016). Gut dysbiosis impairs recovery after spinal cord injury. J Exp Med.

[13] Davis SS, Illum L, Hinchcliffe M. Gastrointestinal transit of dosage forms in the pig. J Pharm Pharmacol. 2001;53(1):33–9. 10.1211/0022357011775163. 10.1211/0022357011775163 11206190

[14] Schomberg DT, Tellez A, Meudt JJ, Brady DA, Dillon KN, Arowolo FK, Wicks J, Rousselle SD, Shanmuganayagam D (2016). Miniature swine for preclinical modeling of complexities of human disease for translational scientific discovery and accelerated development of therapies and medical devices. Toxicol Pathol.

[15] Swindle MM, Smith AC, Laber-laird K, Dungan L. Farm Animals in Biomedical Research — Part One Swine in Biomedical Research: Management and Models. ILAR J. 1994;36(1):1–5. 10.1093/ilar.36.1.1.

[16] Heinritz SN, Mosenthin R, Weiss E. Use of pigs as a potential model for research into dietary modulation of the human gut microbiota. Nutr Res Rev. 2013;26(2):191–209. 10.1017/S0954422413000152. 10.1017/S0954422413000152 24134811

[17] Bellinger DA, Merricks EP, Nichols TC. Swine models of type 2 diabetes mellitus: insulin resistance, Glucose Tolerance, and Cardiovascular Complications. ILAR J. 2006;47(3):243–58. 10.1093/ilar.47.3.243. 10.1093/ilar.47.3.243 16804199

[18] Pedersen R, Ingerslev H, Sturek M, Alloosh M, Cirera S, Christoffersen BØ, et al. Characterisation of Gut Microbiota in Ossabaw and Gottingen Minipigs as Models of Obesity and Metabolic Go Syndrome. PLoS One. 2013. 8(2):1–10. 10.1371/journal.pone.0056612. 10.1371/journal.pone.0056612 PMC3577853 23437186

[19] Lee JHT, Jones CF, Okon EB, Anderson L, Tigchelaar S, Kooner P, Godbey T, Chue B, Gray G, Hildebrandt R, Cripton P, Tetzlaff W, Kwon BK. A novel porcine model of traumatic thoracic spinal cord injury. J Neurotrauma. 2013;30(3):142–59. 10.1089/neu.2012.2386. 10.1089/neu.2012.2386 23316955

[20] Kim KT, Streijger F, Manouchehri N, So K, Short K, Okon EB, Tigchelaar S, Cripton C, Kwon BK. Review of the UBC Porcine Model of Traumatic Spinal Cord Injury. J Korean Neurosurg Soc. 2018;61(5):539–47. 10.3340/jkns.2017.0276. 10.3340/jkns.2017.0276 PMC6129752 30196652

[21] Le Chatelier E, Nielsen T, Qin J, Prifti E, Hildebrand F, Falony G (2013). Richness of human gut microbiome correlates with metabolic markers. Nature..

[22] Falony G, Vieira-Silva S, Raes J. Richness and ecosystem development across faecal snapshots of the gut microbiota. Nat Microbiol. 2018;3(5):526–8. 10.1038/s41564-018-0143-5. 10.1038/s41564-018-0143-5 29693658

[23] Gajer P, Brotman RM, Bai G, Sakamoto J, Schütte UME, Zhong X, et al. Temporal Dynamics of the Human Vaginal Microbiota. Science Translational Medicine. 2012;4(132):132-152. doi: 10.1126/scitranslmed.3003605. 10.1126/scitranslmed.3003605 PMC3722878 22553250

[24] Halfvarson J, Brislawn CJ, Lamendella R, Walters WA, Bramer LM, Bonfiglio F, et al. Dynamics of the human gut microbiome in inflammatory bowel disease. Nature Microbiology. 2017;2:17004. doi: 10.1038/nmicrobiol.2017.4. 10.1038/nmicrobiol.2017.4 PMC5319707 28191884

[25] Carmody LA, Zhao J, Kalikin LM, Lebar W, Simon RH, Venkataraman A, et al. The daily dynamics of cystic fibrosis airway microbiota during clinical stability and at exacerbation. Microbiome. 2015;3(1):1–11. 10.1186/s40168-015-0074-9. 10.1186/s40168-015-0074-9 PMC4381400 25834733

[26] Haegeman B, Hamelin J, Moriarty J, Neal P, Dushoff J, Weitz JS (2013). Robust estimation of microbial diversity in theory and in practice. ISME J.

[27] Almeida A, Mitchell AL, Boland M, Forster SC, Gloor GB, Tarkowska A, et al. A new genomic blueprint of the human gut microbiota. Nature. 2019;568(7753):499–504 .10.1038/s41586-019-0965-1. 10.1038/s41586-019-0965-1 PMC6784870 30745586

[28] Lamendella R, Santo Domingo JW, Ghosh S, Martinson J, Oerther DB. Comparative fecal metagenomics unveils unique functional capacity of the swine gut. BMC Microbiol. 2011;11:103. 10.1186/1471-2180-11-103. 10.1186/1471-2180-11-103 PMC3123192 21575148

[29] Pajarillo EAB, Chae J, Balolong MP, Kim HB, Kang D. Assessment of fecal bacterial diversity among healthy piglets during the weaning transition. J Gen Appl Microbiol. 2014;146(4):140–6. 10.2323/jgam.60.140. 10.2323/jgam.60.140 25273987

[30] Kim S, Covington A, Pamer EG (2017). The intestinal microbiota: antibiotics, colonization resistance, and enteric pathogens. Immunol Rev.

[31] Frese SA, Parker K, Calvert CC, Mills DA. Diet shapes the gut microbiome of pigs during nursing and weaning. Microbiome. 2015;3(1):1–10. 10.1186/s40168-015-0091-8. 10.1186/s40168-015-0091-8 PMC4499176 26167280

[32] Wang X, Tsai T, Deng F, Wei X, Chai J, Knapp J, Apple J, Maxwell CV, Lee JA, Li Y, Zhao J (2019). Longitudinal investigation of the swine gut microbiome from birth to market reveals stage and growth performance associated bacteria. Microbiome..

[33] Lin R, Xu J, Ma Q, Chen M, Wang L, Wen S, et al. Alterations in the fecal microbiota of patients with spinal cord injury. PLoS One. 2020;15(8):1–11. 10.1371/journal.pone.0236470. 10.1371/journal.pone.0236470 PMC7402510 32750057

[34] Caporaso JG, Lauber CL, Costello EK, Berg-lyons D, Gonzalez A, Stombaugh J, et al. Moving pictures of the human microbiome. Genome Biol. 2011;5(5):R50. 10.1186/gb-2011-12-5-r50. 10.1186/gb-2011-12-5-r50 PMC3271711 21624126

[35] Faith JJ, Guruge JL, Charbonneau M, Subramanian S, Seedorf H, Goodman AL, et al. The Long-Term Stability of the Human Gut Microbiota. Sci. 2013;340(6141). 10.1126/science.1237439. 10.1126/science.1237439 PMC3791589 23828941

[36] Weinstock GM (2011). The volatile microbiome. Genome Biol.

[37] Goodrich JK, Di Rienzi SC, Poole AC, Koren O, Walters WA, Caporaso JG (2014). Conducting a microbiome study. Cell..

[38] Bastiaanssen TFS, Gururajan A, van de Wouw M, Moloney GM, Ritz NL, Long-Smith CM, et al. Volatility as a Concept to Understand the Impact of Stress on the Microbiome. Psychoneuroendocrinology. 2021;124:105047. 10.1016/j.psyneuen.2020.105047. 10.1016/j.psyneuen.2020.105047 33307493

[39] Thomas F, Hehemann JH, Rebuffet E, Czjzek M, Michel G. Environmental and gut bacteroidetes : the food connection. Front Microbiol. 2011;2:1–16. 10.3389/fmicb.2011.00093. 10.3389/fmicb.2011.00093 PMC3129010 21747801

[40] Zhang C, Zhang W, Zhang J, Jing Y, Yang M, Du L, et al. Gut microbiota dysbiosis in male patients with chronic traumatic complete spinal cord injury. J Transl Med. 2018;16(1):1–16. 10.1186/s12967-018-1735-9. 10.1186/s12967-018-1735-9 PMC6293533 30545398

[41] Gungor B, Adiguzel E, Gursel I, Yilmaz B, Gursel M. Intestinal microbiota in patients with spinal cord injury. PLoS One. 2016;11(1):1–10, e0145878. 10.1371/journal.pone.0145878. 10.1371/journal.pone.0145878 PMC4709077 26752409

[42] O’Connor G, Jeffrey E, Madorma D, Marcillo A, Abreu MT, Deo SK, et al. Investigation of microbiota alterations and intestinal inflammation post-spinal cord injury in rat model. J Neurotrauma. 2018;35(18):2159–66. 10.1089/neu.2017.5349. 10.1089/neu.2017.5349 PMC6119224 29566601

[43] Galperin MY. Genome diversity of spore-forming Firmicutes bacterial systematics from gram stain to 16S rRNA. Microbiol Spectr. 2013;1(2):1–27. 10.1128/microbiolspectrum.TBS-0015-2012. 10.1128/microbiolspectrum.TBS-0015-2012 PMC4306282 26184964

[44] Park J, Woo M, Kim S, Kim W, Kim H. Repression of interferon- g -induced inducible nitric oxide synthase ( iNOS ) gene expression in microglia by sodium butyrate is mediated through specific inhibition of ERK signaling pathways. J Neuroimmunol. 2005;168(1-2):56–64. 10.1016/j.jneuroim.2005.07.003. 10.1016/j.jneuroim.2005.07.003 16091294

[45] Chen PS, Wang C, Bortner CD, Peng GS, Wu X, Pang H, Lu RB, Gean PW, Chuang DM, Hong JS. Valproic acid and other histone deacetylase inhibitors induce microglial apoptosis and attenuate lipopolysaccharide-induced dopaminergic neurotoxicity. Neurosci. 2007;149(1):203–12. 10.1016/j.neuroscience.2007.06.053. 10.1016/j.neuroscience.2007.06.053 PMC2741413 17850978

[46] Wikoff WR, Anfora AT, Liu J, Schultz PG, Lesley SA, Peters EC, Siuzdak G. Metabolomics analysis reveals large effects of gut microflora on mammalian blood metabolites. PNAS. 2009;106(10):3698–703. 10.1073/pnas.0812874106. 10.1073/pnas.0812874106 PMC2656143 19234110

[47] Clarke G, Stilling RM, Kennedy PJ, Stanton C, Cryan JF, Dinan TG. Minireview: Gut microbiota: The neglected endocrine organ. Molecular Endocrinology. 2014;28(8):1221–38. 10.1210/me.2014-1108. 10.1210/me.2014-1108 PMC5414803 24892638

[48] Forsythe P, Bienenstock J, Kunze WA. Vagal Pathways for Microbiome-Brain-Gut Axis Communication. Advances in Experimental Medicine and Biology. 2014;817:115–33. 10.1007/978-1-4939-0897-4_5. 10.1007/978-1-4939-0897-4_5 24997031

[49] Kigerl KA, Hall JCE, Wang L, Mo X, Yu Z, Popovich PG. Gut dysbiosis impairs recovery after spinal cord injury. J Exp Med. 2016;213(12):2603–20. 10.1084/jem.20151345. 10.1084/jem.20151345 PMC5110012 27810921

[50] Dethlefsen L, Relman DA. Incomplete recovery and individualized responses of the human distal gut microbiota to repeated antibiotic perturbation. Proc Natl Acad Sci U S A. 2011;108(1):4554–61. 10.1073/pnas.1000087107. 10.1073/pnas.1000087107 PMC3063582 20847294

[51] Huse SM, Dethlefsen L, Huber JA, Welch DM, Relman DA, Sogin ML. Exploring microbial diversity and taxonomy using SSU rRNA hypervariable tag sequencing. PLoS Genet. 2008;4(11):e1000255. 10.1371/journal.pgen.1000255. 10.1371/journal.pgen.1000255 PMC2577301 19023400

[52] Jernberg C, Löfmark S, Edlund C, Jansson JK. Long-term impacts of antibiotic exposure on the human intestinal microbiota. Microbiology. 2010;156(11):3216–23. 10.1099/mic.0.040618-0. 10.1099/mic.0.040618-0 20705661

[53] Koo H, Hakim JA, Crossman DK, Kumar R, Lefkowitz EJ, Morrow CD. Individualized recovery of gut microbial strains post antibiotics. Biofilms Microbiomes. 2019;5(1):1–6. 10.1038/s41522-019-0103-8. 10.1038/s41522-019-0103-8 PMC6789009 31632686

[54] Miklossy J. Biology and neuropathology of dementia in syphilis and Lyme disease. Handbook of Clinical Neurology. 2008;89:825–44. 10.1016/S0072-9752(07)01272-9. 10.1016/S0072-9752(07)01272-9 18631798

[55] Beaman BL, Beaman L (1994). Nocardia species: host-parasite relationships. Clin Microbiol Rev.

[56] Riviere G, Riviere K, Smith K (2002). Molecular and immunological evidence of oral Treponema in the human brain and their association with Alzheimer ’ s disease. Oral Microbiol Immunol.

[57] Miklossy J, Kis A, Radenovic A, Miller L, Forro L, Martins R. et al, Beta-amyloid deposition and Alzheimer ’ s type changes induced by Borrelia spirochetes. Neurobiol Aging. 2006;27(2):228–36. 10.1016/j.neurobiolaging.2005.01.018. 10.1016/j.neurobiolaging.2005.01.018 15894409

[58] Claesson MJ, Jeffery IB, Conde S, Power SE, O'Connor EM, Cusack S, Harris HMB, Coakley M, et al. Gut microbiota composition correlates with diet and health in the elderly. Nature. 2012;88(7410):178–84. 10.1038/nature11319. 10.1038/nature11319 22797518

[59] Stanton C, Sandhu KV, Sherwin E, Et H, Dinan TG, Cryan JF. Feeding the microbiota-gut-brain axis: diet, microbiome, and neuropsychiatry. Transl Res. 2016;179:223–44. 10.1016/j.trsl.2016.10.002. 10.1016/j.trsl.2016.10.002 27832936

[60] De Filippo C, Cavalieri D, Di M, Ramazzotti M, Baptiste J. Impact of diet in shaping gut microbiota revealed by a comparative study in children from Europe and rural Africa. PNAS. 2010;107(33):14691–6. 10.1073/pnas.1005963107. 10.1073/pnas.1005963107 PMC2930426 20679230

[61] Suez J, Korem T, Zeevi D, Zilberman-schapira G, Thaiss CA, Maza O, et al. Artificial sweeteners induce glucose intolerance by altering the gut microbiota. Nature. 2014;514(7521):181–6. 10.1038/nature13793. 10.1038/nature13793 25231862

[62] Chassaing B, Koren O, Goodrich JK, Poole AC, Srinivasan S, Ley RE, et al. Promoting colitis and metabolic syndrome. Nature. 2015;519(7541):92–6. 10.1038/nature14232. 10.1038/nature14232 PMC4910713 25731162

[63] Cryan JF, Dinan TG (2012). Mind-altering microorganisms: the impact of the gut microbiota on brain and behaviour. Nat Rev Neurosci.

[64] Erny D, De Angelis ALH, Jaitin D, Wieghofer P, Staszewski O, David E (2015). Host microbiota constantly control maturation and function of microglia in the CNS. Nat Neurosci.

[65] Furusawa Y, Obata Y, Fukuda S, Endo TA, Nakato G, Takahashi D, Nakanishi Y, Uetake C, Kato K, Kato T, Takahashi M, Fukuda NN, Murakami S, Miyauchi E, Hino S, Atarashi K, Onawa S, Fujimura Y, Lockett T, Clarke JM, Topping DL, Tomita M, Hori S, Ohara O, Morita T, Koseki H, Kikuchi J, Honda K, Hase K, Ohno H (2013). Commensal microbe-derived butyrate induces the differentiation of colonic regulatory T cells. Nature..

[66] Myers SA, Gobejishvili L, Saraswat S, Wilson CG, Andres KR, Riegler AS, et al. Following spinal cord injury , PDE4B drives an acute , local in fl ammatory response and a chronic , systemic response exacerbated by gut dysbiosis and endotoxemia. Neurobiol Dis. 2019;124:353–63. 10.1016/j.nbd.2018.12.008. 10.1016/j.nbd.2018.12.008 PMC6445388 30557659

[67] Xia G, You C, Gao X, Zeng X, Zhu J, Xu K. Stroke Dysbiosis Index (SDI) in Gut Microbiome Are Associated With Brain Injury and Prognosis of Stroke. Front Neurol. 2019;10:1–13. 10.3389/fneur.2019.00397. 10.3389/fneur.2019.00397 PMC6491752 31068891

[68] Zhang L, Huang Y, Zhou Y, Buckley T, Wang HH (2013). Antibiotic administration routes significantly influence the levels of antibiotic resistance in gut microbiota. Antimicrob Agents Chemother.

[69] Tigchelaar S, Streijger F, Sinha S, Flibotte S, Manouchehri N, So K, Shortt K, Okon E, Rizzuto MA, Malenica I, Courtright-Lim A, Eisen A, Keuren-Jensen KV, Nislow C, Kwon BK (2017). Serum MicroRNAs reflect Injury severity in a large animal model of thoracic spinal Cord Injury. Sci Rep.

[70] Streijger F, Lee JHT, Chak J, Dressler D, Manouchehri N, Okon EB, Anderson LM, Melnyk AD, Cripton PA, Kwon BK (2015). The effect of whole-body resonance vibration in a porcine model of spinal cord injury. J Neurotrauma.

[71] Keung MS, Streijger F, Herrity A, Ethridge J, Dougherty SM, Aslan S, et al. Characterization of Lower Urinary Tract Dysfunction after Thoracic Spinal Cord Injury in Yucatan Mini-Pigs. J Neurotrauma. 2021;38(9):1–63. 10.1089/neu.2020.7404. 10.1089/neu.2020.7404 33499736

[72] Cheung A, Tu L, Manouchehri N, Kim KT, So K, Webster M, Fisk S, Tigchelaar S, Dalkilic SS, Sayre EC, Streijger F, Macnab A, Kwon BK, Shadgan B (2020). Continuous optical monitoring of spinal Cord oxygenation and hemodynamics during the first seven days post-Injury in a porcine model of acute spinal Cord Injury. J Neurotrauma.

[73] Williams AM, Manouchehri N, Erskine E, Tauh K, So K, Shortt K, et al. Cardio-centric hemodynamic management improves spinal cord oxygenation and mitigates hemorrhage in acute spinal cord injury. Nat Commun. 2020;11(1):1–12. 10.1038/s41467-020-18905-8. 10.1038/s41467-020-18905-8 PMC7562705 33060602

[74] West CR, Poormasjedi-Meibod MS, Manouchehri N, Williams AM, Erskine EL, Webster M, Fisk S, Morrison C, Short K, So K, Cheung A, Streijger F, Kwon BK (2020). A porcine model for studying the cardiovascular consequences of high-thoracic spinal cord injury. J Physiol.

