# Correction to: Characterization of the gut microbiome in a porcine model of thoracic spinal cord injury

**DOI:** 10.1186/s12864-021-08202-z

**Published:** 2021-12-06

**Authors:** Adam Doelman, Seth Tigchelaar, Brian McConeghy, Sunita Sinha, Martin S. Keung, Neda Manouchehri, Megan Webster, Shera Fisk, Charlotte Morrison, Femke Streijger, Corey Nislow, Brian K. Kwon

**Affiliations:** 1 International Collaboration on Repair Discoveries, University of British Columbia, Vancouver, BC Canada; 2 Sequencing and Bioinformatics Consortium, University of British Columbia, Vancouver, BC Canada; 3 Department of Orthopedics, Vancouver Spine Surgery Institute, University of British Columbia, Vancouver, BC Canada

**Correction to: BMC Genomics 22, 775 (2021)**

**https://doi.org/10.1186/s12864-021-07979-3**

Following publication of the original article [1], it was reported that the figure legends did not correspond to the correct figures. The legends for figures 1 – 9 were mistakenly assigned to Figs. 8, 9, 1, 2, 3, 4, 5, 6 and 7 respectively.

The figures legends now correspond to the correct figures and the original article [1] has been updated.
