# Supplementary material for: Characterization of the gut microbiome in a porcine model of thoracic spinal cord injury
Source: BMC Genomics. 2021 Oct 30;22:775. doi: 10.1186/s12864-021-07979-3 (PMC8557039; doi:10.1186/s12864-021-07979-3)
Supplement: Supplementary file 1 — Additional file 1. [file 12864_2021_7979_MOESM1_ESM.docx]

**Table 1.**

| **Phylum** | **ANOVA** | | **Control (n=93)** | **Antibiotics (n=45)** | | **Diet (n=45)** | | **SCI-Acute (n=58)** | | **SCI-Subacute (n=20)** | |
| --- | --- | --- | --- | --- | --- | --- | --- | --- | --- | --- | --- |
|  | F | p-value | % | % | p | % | p | % | p | % | p |
| **Firmicutes** | 11.49 | <0.0001 | 11.18 | 11.43 | 0.167 | 11.05 | 0.392 | 12.10 | 0.000 | 11.04 | 0.442 |
| **Bacteroidetes** | 4.592 | 0.0013 | 10.93 | 10.54 | 0.036 | 10.74 | 0.169 | 11.25 | 0.044 | 10.91 | 0.864 |
| **Spirochaetes** | 37.01 | <0.0001 | 7.32 | 5.17 | 0.000 | 7.18 | 0.395 | 9.39 | 0.000 | 8.26 | 0.001 |
| **Proteobacteria** | 7.278 | <0.0001 | 6.51 | 6.56 | 0.885 | 7.40 | 0.0004 | 5.95 | 0.033 | 5.77 | 0.009 |
| **Tenericutes** | 6.877 | <0.0001 | 4.87 | 4.05 | 0.005 | 3.96 | 0.000 | 3.46 | 0.000 | 4.69 | 0.536 |
| Patescibacteria | 2.058 | 0.0868 | 3.63 | 4.12 | 0.241 | 3.56 | 0.868 | 3.56 | 0.883 | 1.97 | 0.068 |
| Actinobacteria | 0.983 | 0.4173 | 3.36 | 3.70 | 0.523 | 2.93 | 0.477 | 4.03 | 0.174 | 3.66 | 0.626 |
| **Epsilonbacteraeota** | 10.31 | <0.0001 | 2.87 | 1.42 | 0.003 | 4.01 | 0.001 | 0.86 | 0.001 | 1.73 | 0.169 |
| **Cyanobacteria** | 40.59 | <0.0001 | 2.35 | 3.22 | 0.067 | 3.07 | 0.079 | -1.89 | 0.000 | -1.67 | 0.000 |
| **Fibrobacteres** | 2.413 | 0.0495 | 0.96 | 0.26 | 0.289 | 1.48 | 0.429 | -0.07 | 0.143 | 2.56 | 0.093 |

**Supplemental Table 1.** Spinal cord injury impact parameters.

| **Animal ID** | **Injury Level** | **Impact Height (cm)** | **Compression Time (min)** | **Impact Force (kdynes)** | **Impulse (kdynes*s)** | **Displacement (mm)** | **Vcontact (mm/s)** |
| --- | --- | --- | --- | --- | --- | --- | --- |
| 6502 | T10 | 20 | 5 | 2557.9 | 9.89 | 3.51 | 1802.69 |
| 6504 | T10 | 20 | 5 | 3170.52 | 10.28 | 3.56 | 1807.19 |
| 6134 | T2 | 20 | 120 | 3612.3 | 12.12 | 3.94 | 1915.48 |
| 6135 | T2 | 20 | 120 | 2376.54 | 10.99 | 3.51 | 1772.18 |
| 6506 | T10 | 20 | 30 | 4270.77 | 12.39 | 4.47 | 1988.69 |
| 6507 | T10 | 20 | 30 | 3758.58 | 11.77 | 3.9 | 1973.54 |
| 9514 | T10 | 20 | 5 | 3066.02 | 11.56 | 4.37 | 1643.29 |
| 9524 | T10 | 20 | 5 | 3338.09 | 11.4 | 4.26 | 1668.25 |

|  |  |  |  |  |  |  |  |
| --- | --- | --- | --- | --- | --- | --- | --- |
|  |  |  |  |  |  |  |  |
|  |  |  |  |  |  |  |  |
|  |  |  |  |  |  |  |  |
|  |  |  |  |  |  |  |  |
|  |  |  |  |  |  |  |  |
|  |  |  |  |  |  |  |  |
|  |  |  |  |  |  |  |  |
|  |  |  |  |  |  |  |  |

|  |  |  |  |  |  |  |  |
| --- | --- | --- | --- | --- | --- | --- | --- |
|  |  |  |  |  |  |  |  |
|  |  |  |  |  |  |  |  |
|  |  |  |  |  |  |  |  |
|  |  |  |  |  |  |  |  |
|  |  |  |  |  |  |  |  |
|  |  |  |  |  |  |  |  |
|  |  |  |  |  |  |  |  |
|  |  |  |  |  |  |  |  |
