# Supplementary figures and images for: Characterization of the gut microbiome in a porcine model of thoracic spinal cord injury
Source: BMC Genomics. 2021 Oct 30;22:775. doi: 10.1186/s12864-021-07979-3 (PMC8557039; doi:10.1186/s12864-021-07979-3)

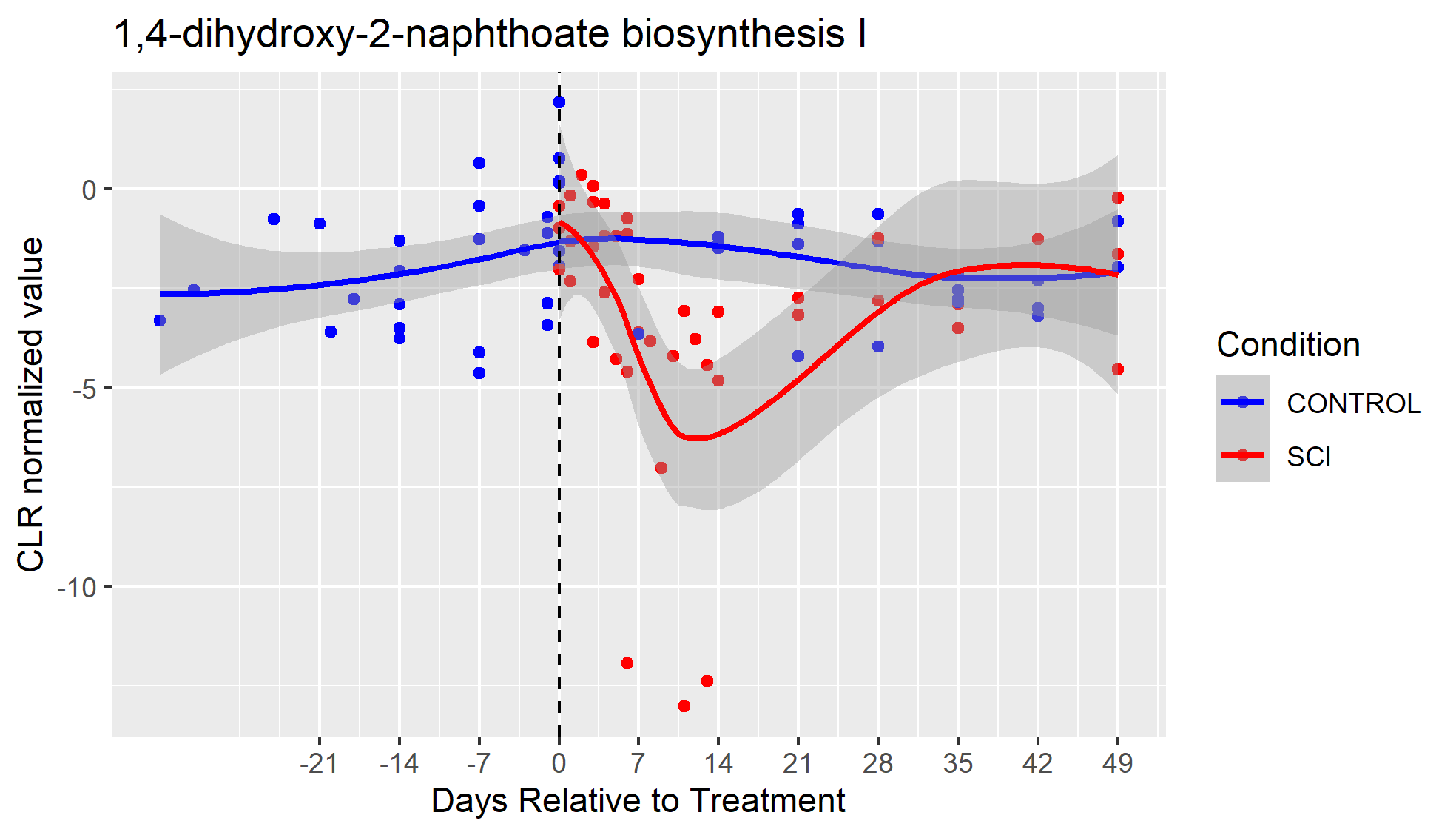

Supplement: Supplementary file 3 — Additional file 3. [file 12864_2021_7979_MOESM3_ESM.zip › pathways_SCI_vs_CONTROL_1,4-dihydroxy-2-naphthoate_biosynthesis_I.png]

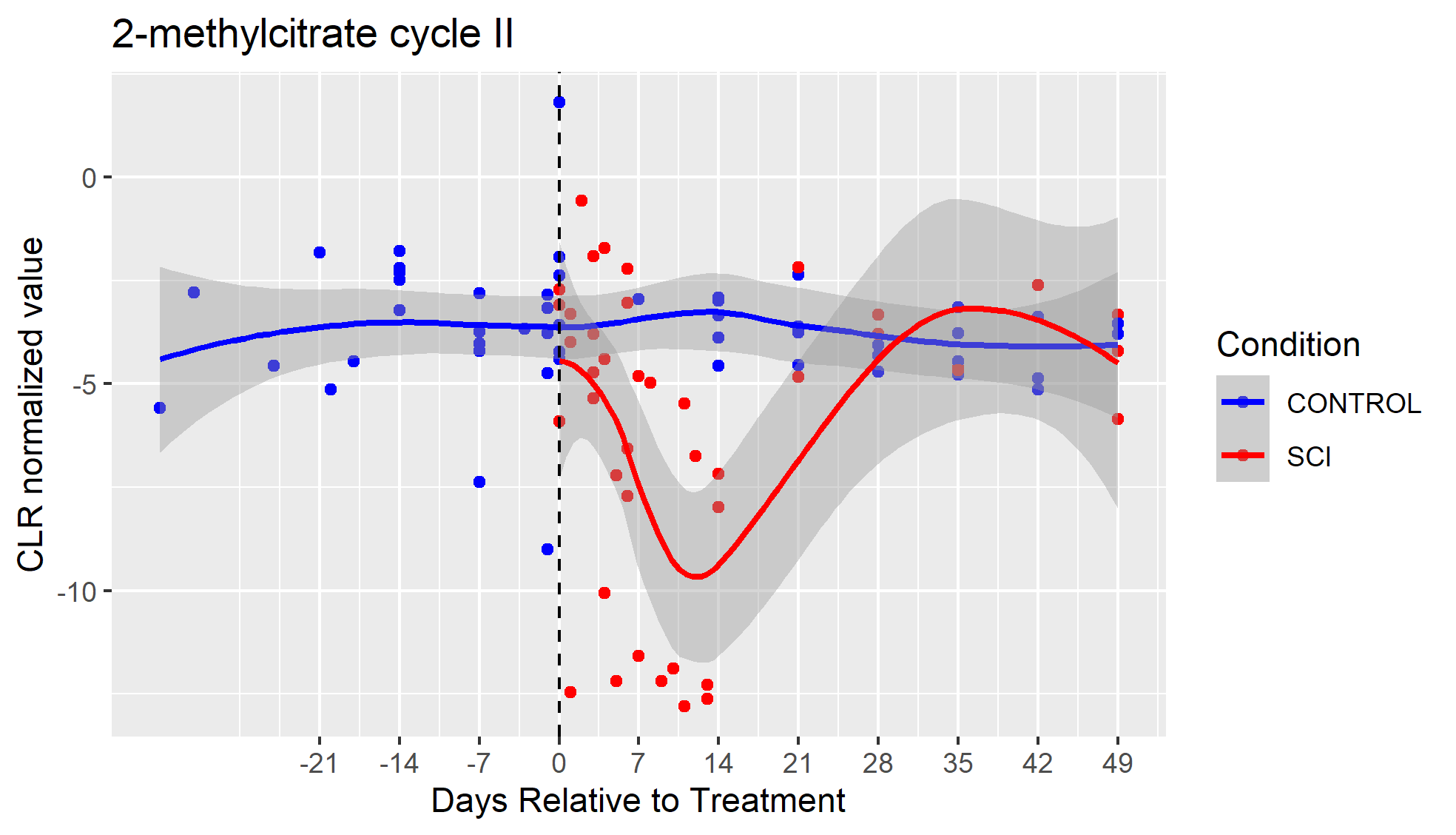

Supplement: Supplementary file 3 — Additional file 3. [file 12864_2021_7979_MOESM3_ESM.zip › pathways_SCI_vs_CONTROL_2-methylcitrate_cycle_II.png]

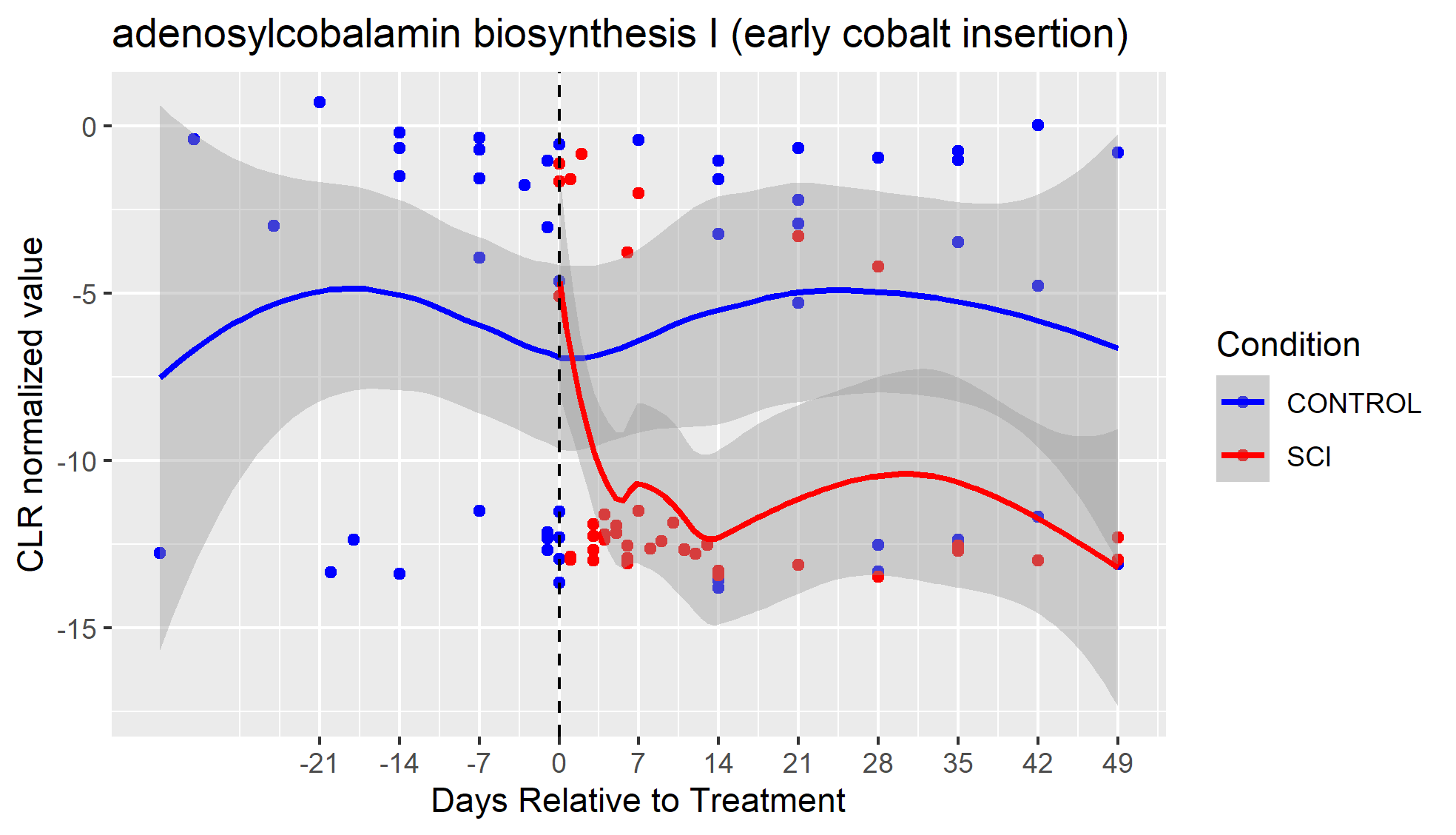

Supplement: Supplementary file 3 — Additional file 3. [file 12864_2021_7979_MOESM3_ESM.zip › pathways_SCI_vs_CONTROL_adenosylcobalamin_biosynthesis_I_(early_cobalt_insertion).png]

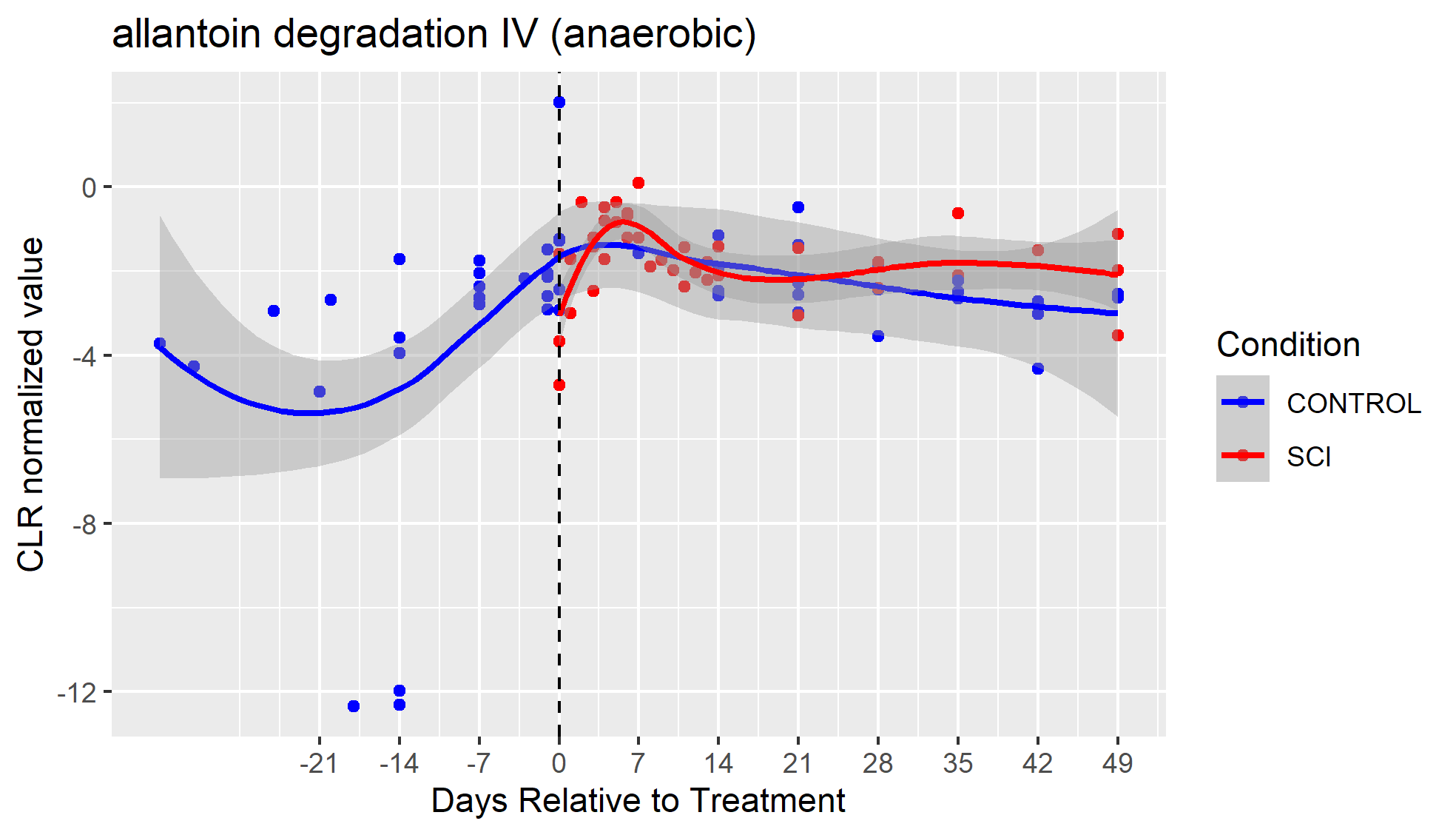

Supplement: Supplementary file 3 — Additional file 3. [file 12864_2021_7979_MOESM3_ESM.zip › pathways_SCI_vs_CONTROL_allantoin_degradation_IV_(anaerobic).png]

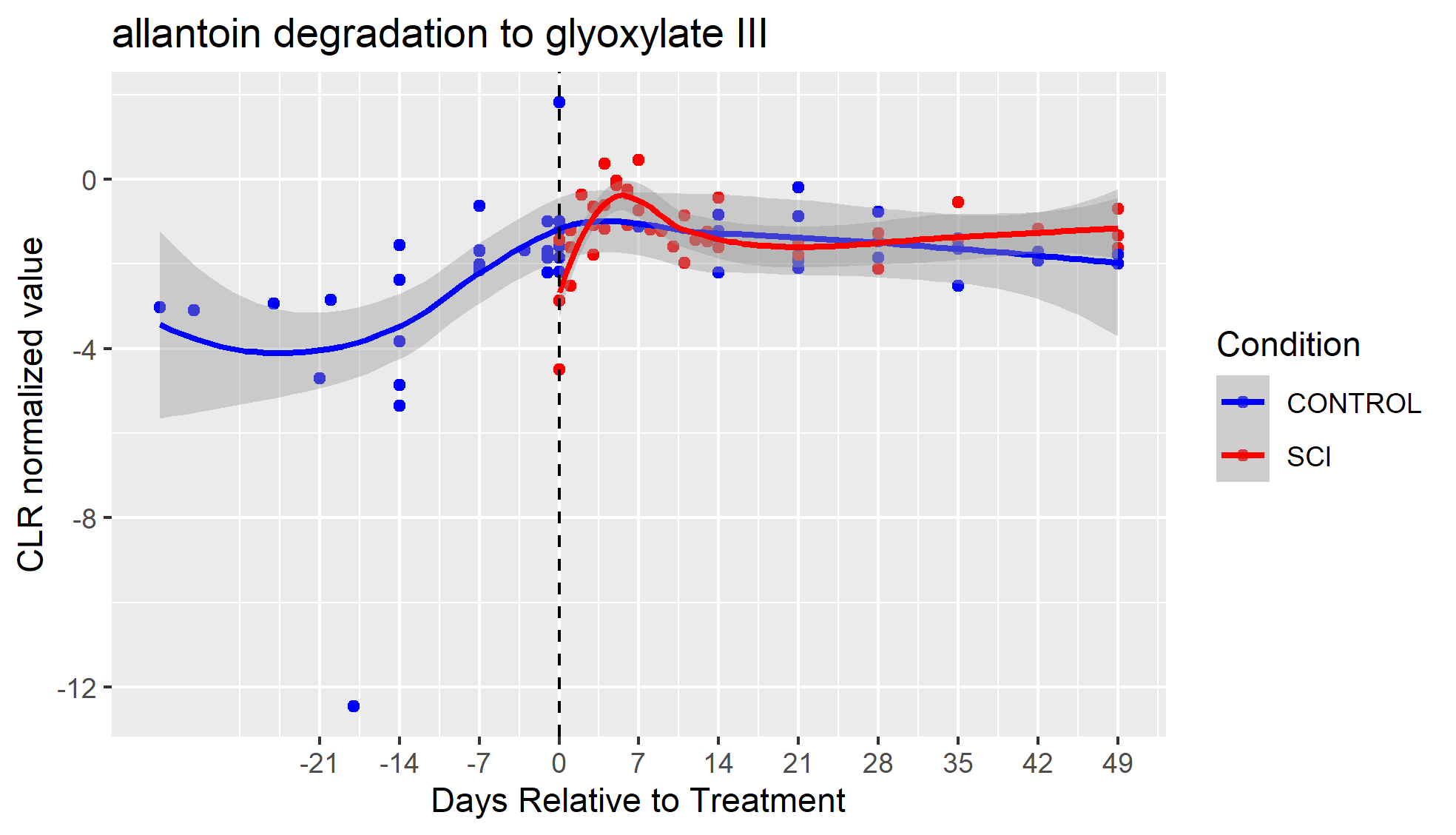

Supplement: Supplementary file 3 — Additional file 3. [file 12864_2021_7979_MOESM3_ESM.zip › pathways_SCI_vs_CONTROL_allantoin_degradation_to_glyoxylate_III.png]

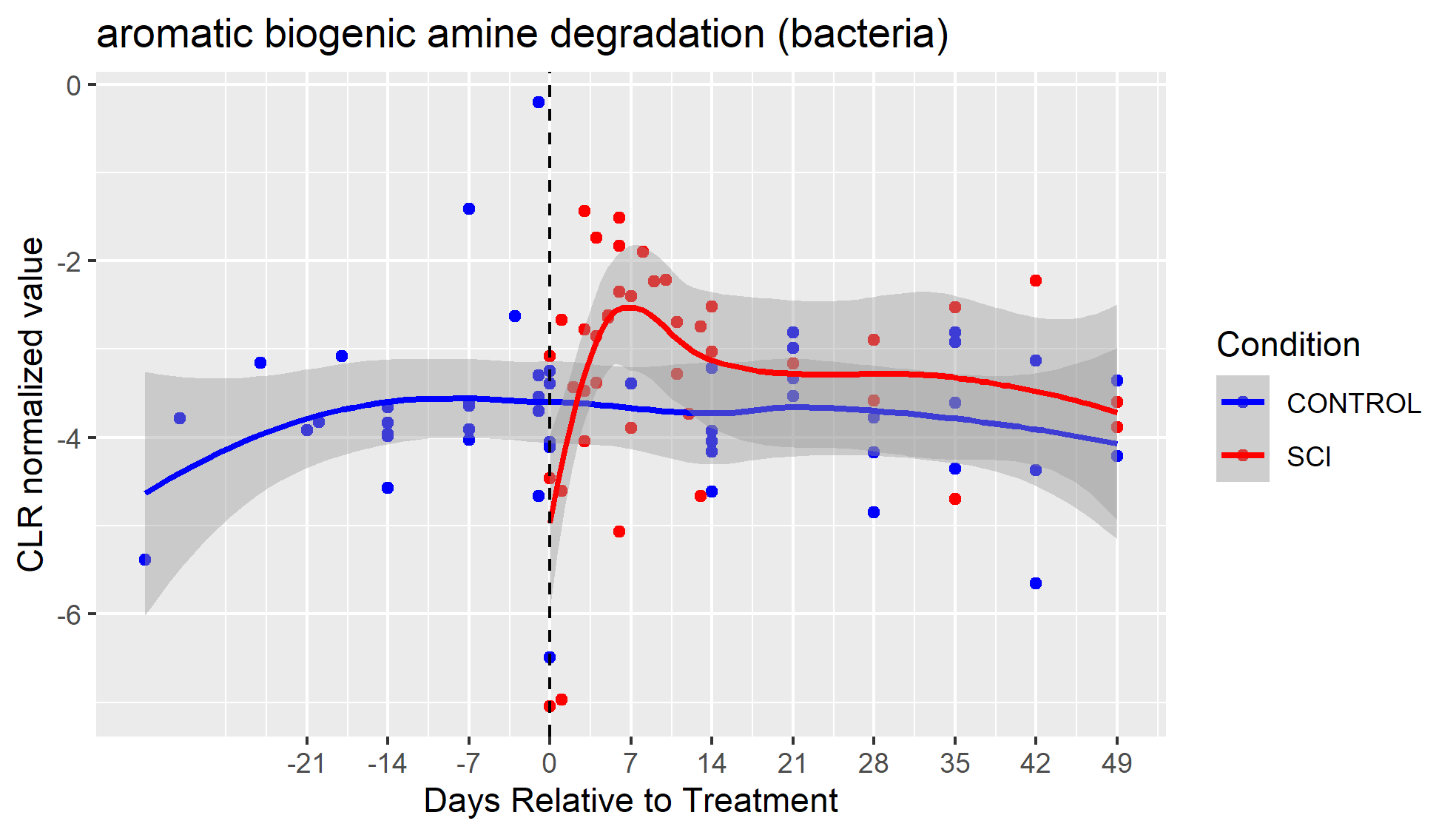

Supplement: Supplementary file 3 — Additional file 3. [file 12864_2021_7979_MOESM3_ESM.zip › pathways_SCI_vs_CONTROL_aromatic_biogenic_amine_degradation_(bacteria).png]

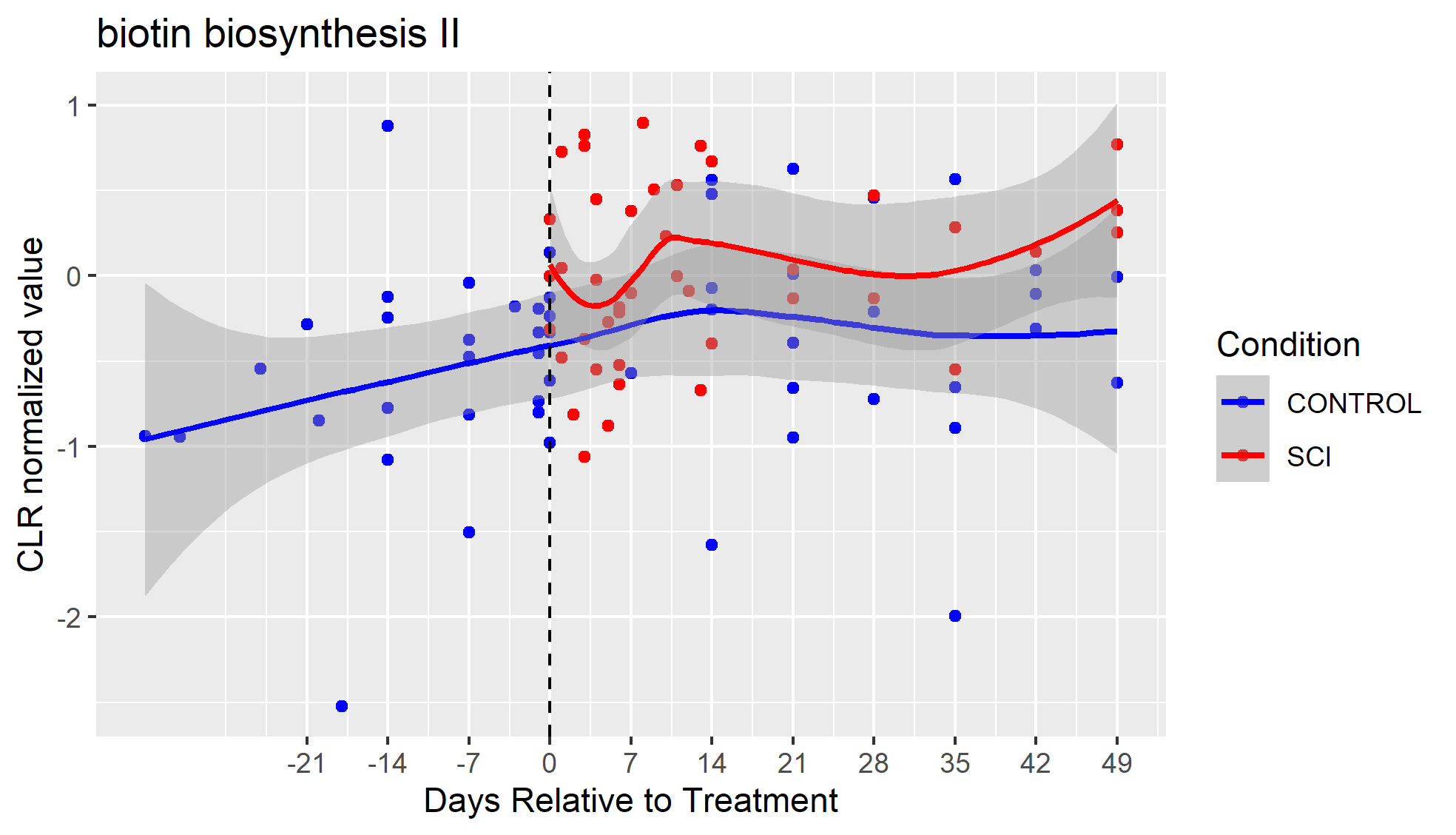

Supplement: Supplementary file 3 — Additional file 3. [file 12864_2021_7979_MOESM3_ESM.zip › pathways_SCI_vs_CONTROL_biotin_biosynthesis_II.png]

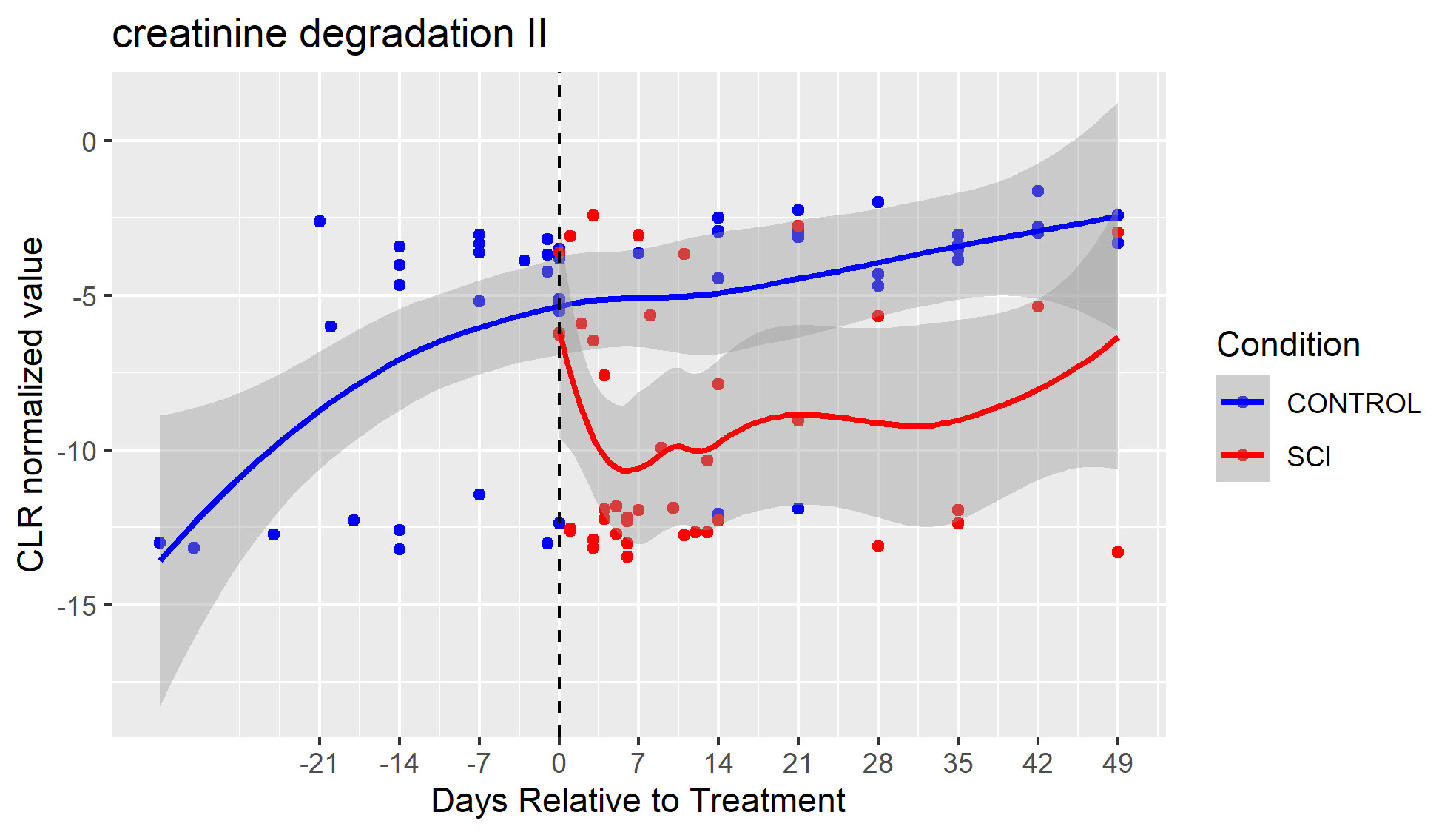

Supplement: Supplementary file 3 — Additional file 3. [file 12864_2021_7979_MOESM3_ESM.zip › pathways_SCI_vs_CONTROL_creatinine_degradation_II.png]

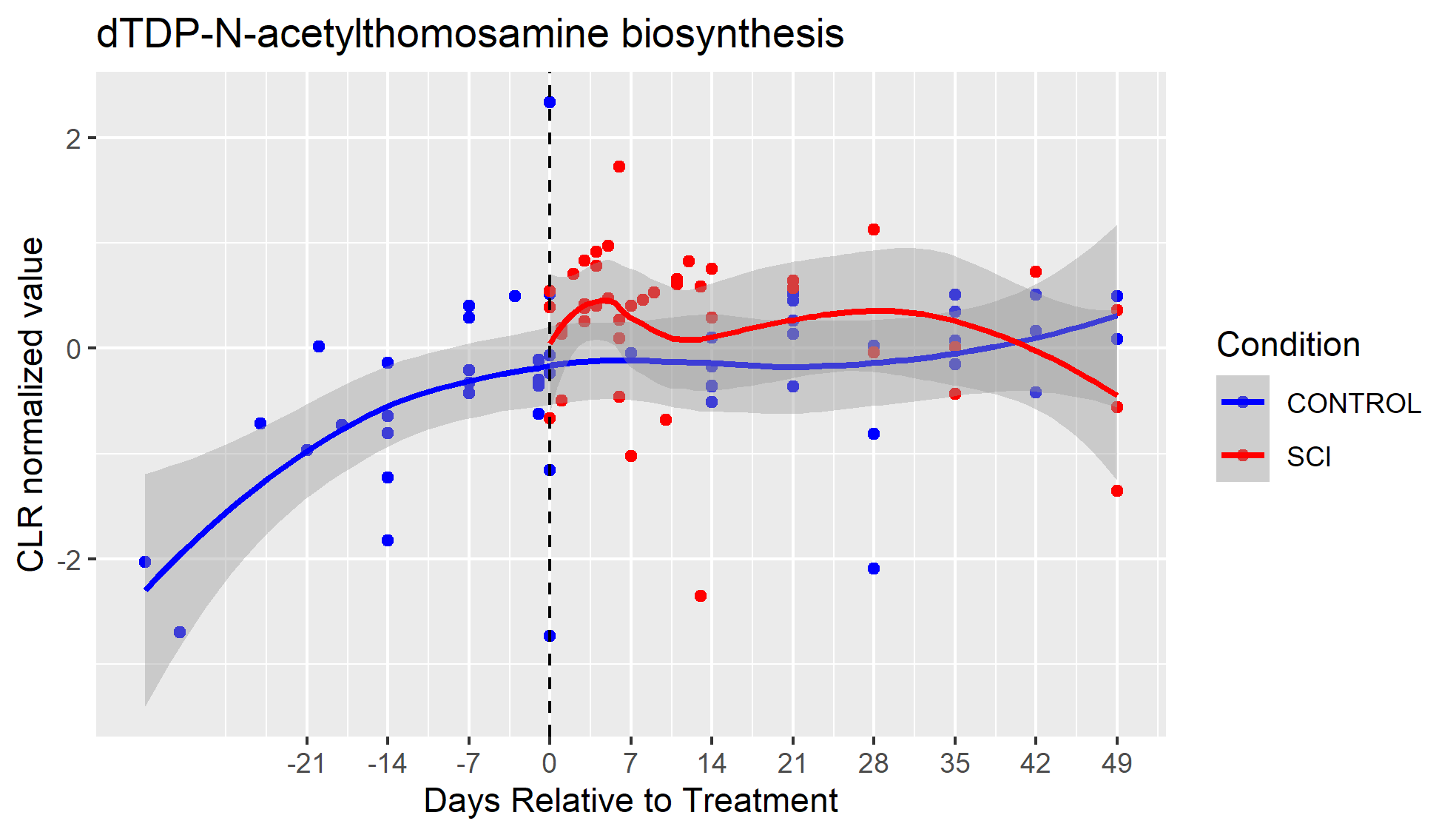

Supplement: Supplementary file 3 — Additional file 3. [file 12864_2021_7979_MOESM3_ESM.zip › pathways_SCI_vs_CONTROL_dTDP-N-acetylthomosamine_biosynthesis.png]

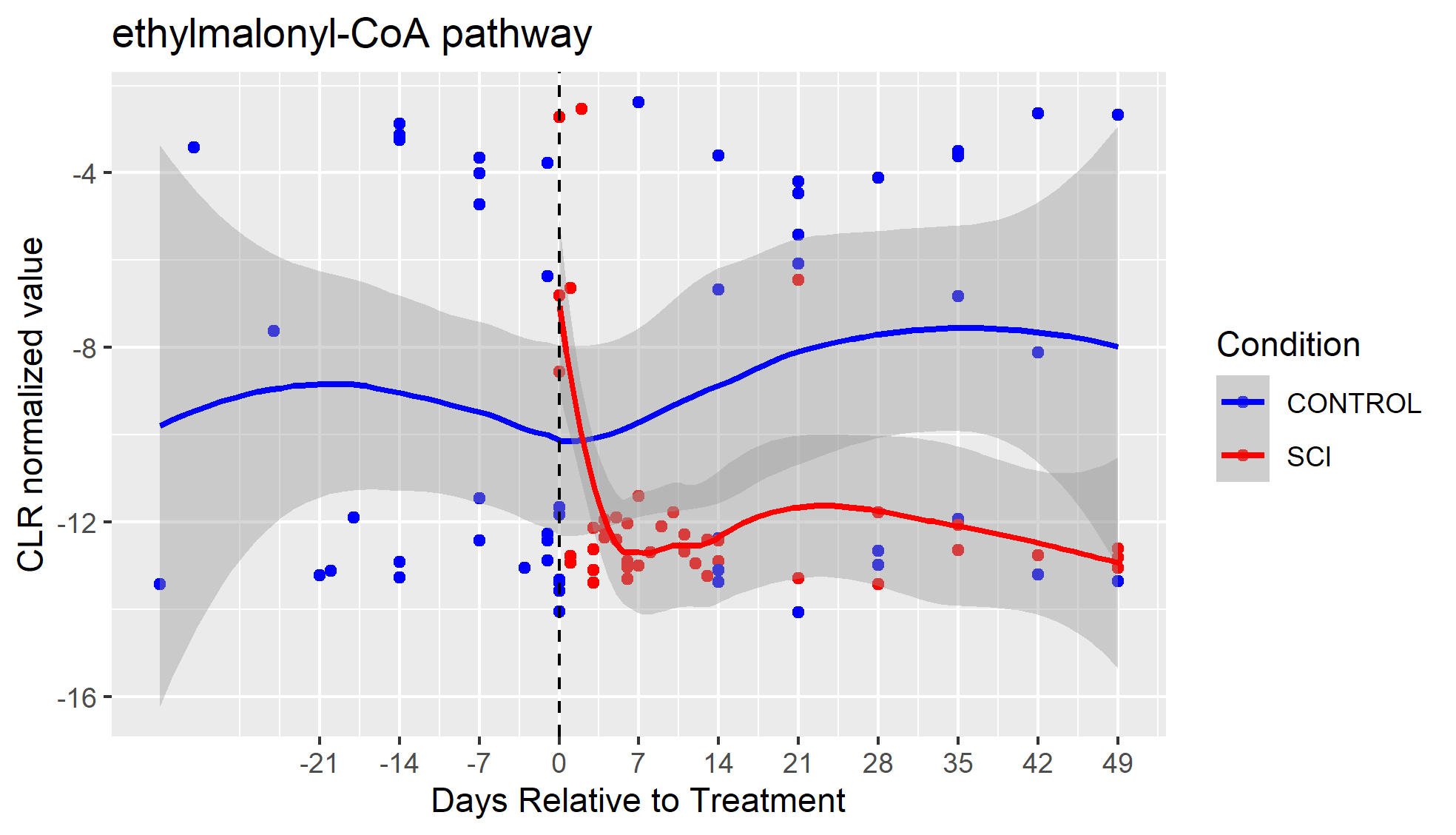

Supplement: Supplementary file 3 — Additional file 3. [file 12864_2021_7979_MOESM3_ESM.zip › pathways_SCI_vs_CONTROL_ethylmalonyl-CoA_pathway.png]

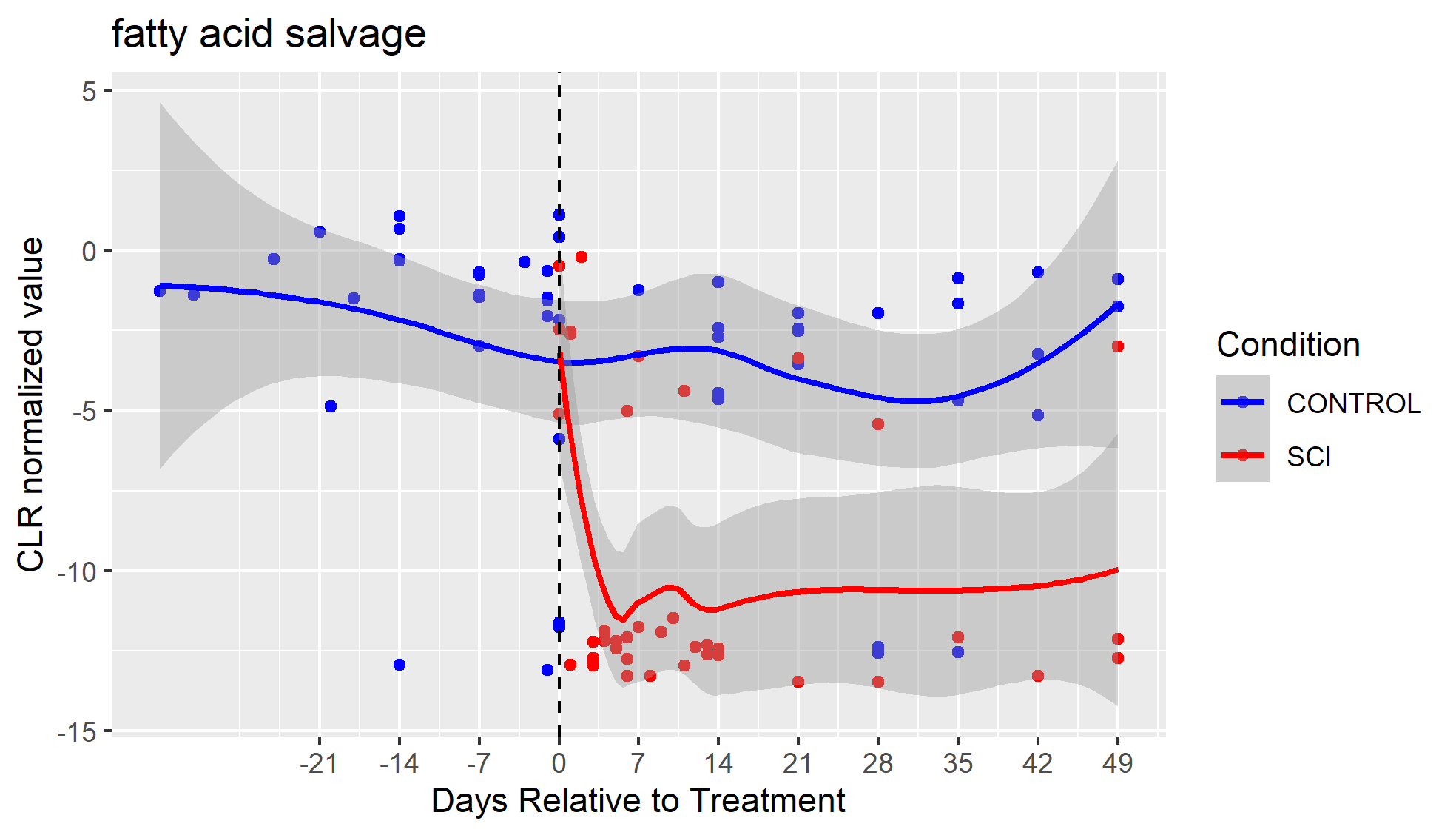

Supplement: Supplementary file 3 — Additional file 3. [file 12864_2021_7979_MOESM3_ESM.zip › pathways_SCI_vs_CONTROL_fatty_acid_salvage.png]

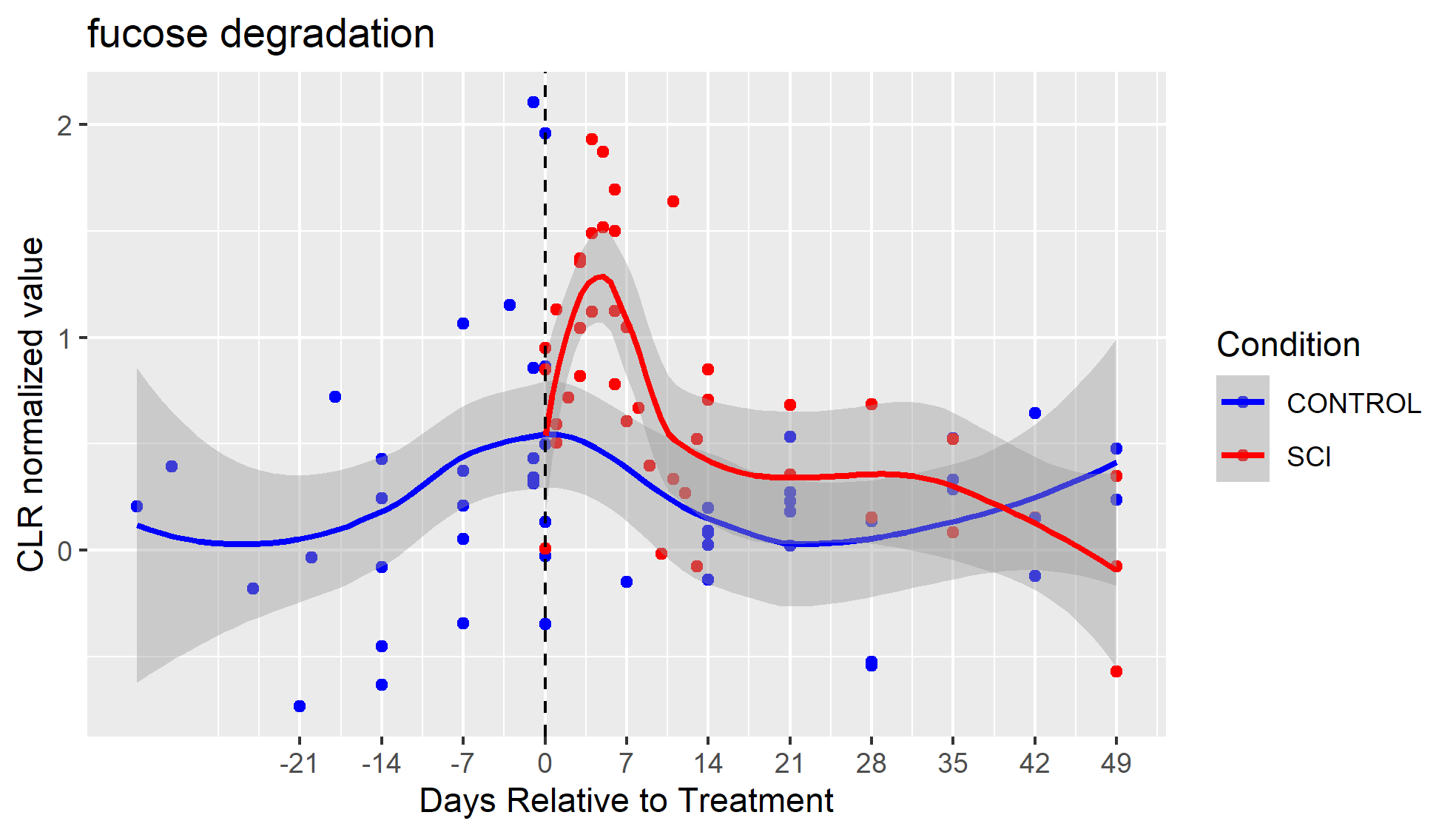

Supplement: Supplementary file 3 — Additional file 3. [file 12864_2021_7979_MOESM3_ESM.zip › pathways_SCI_vs_CONTROL_fucose_degradation.png]

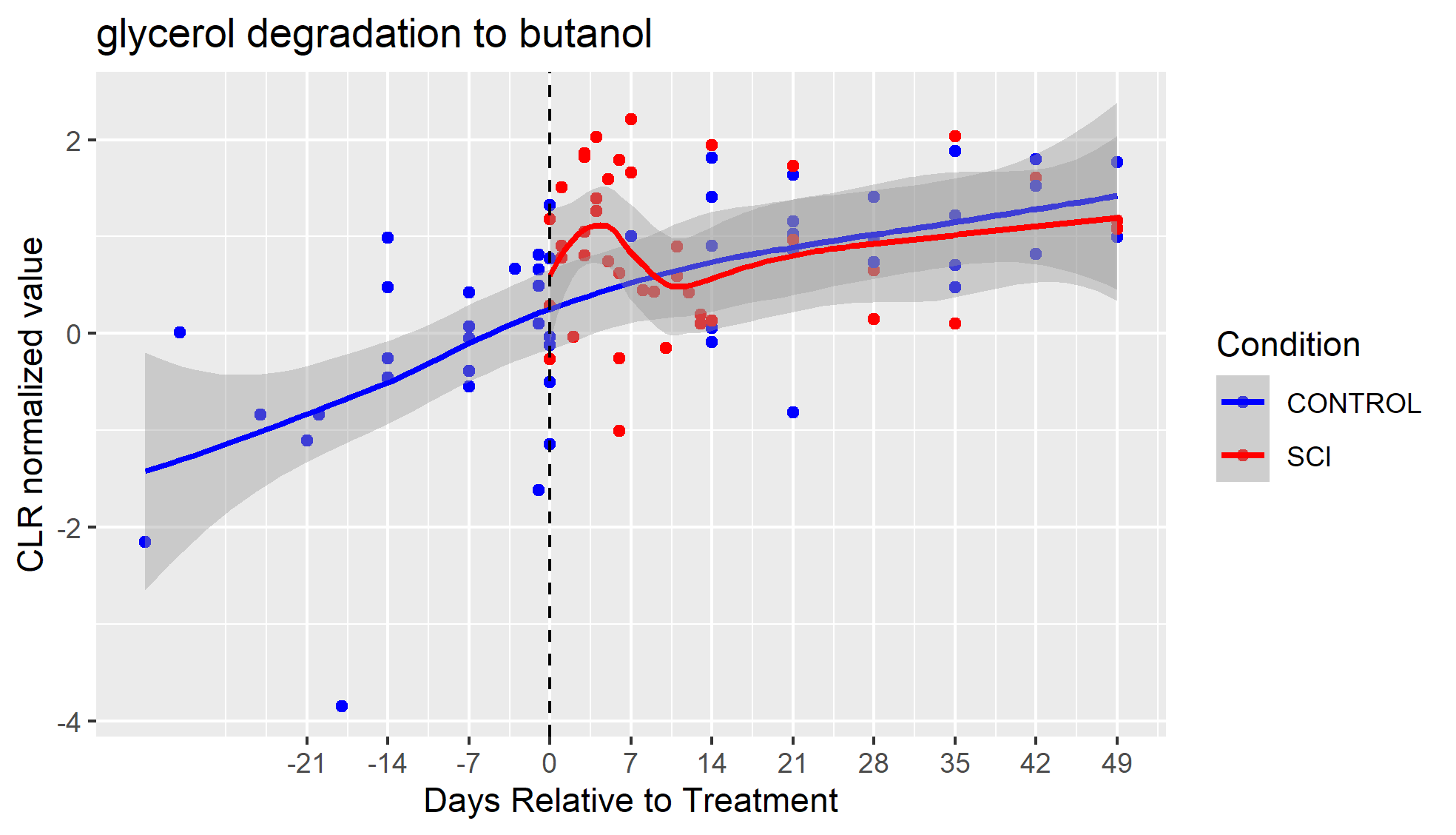

Supplement: Supplementary file 3 — Additional file 3. [file 12864_2021_7979_MOESM3_ESM.zip › pathways_SCI_vs_CONTROL_glycerol_degradation_to_butanol.png]

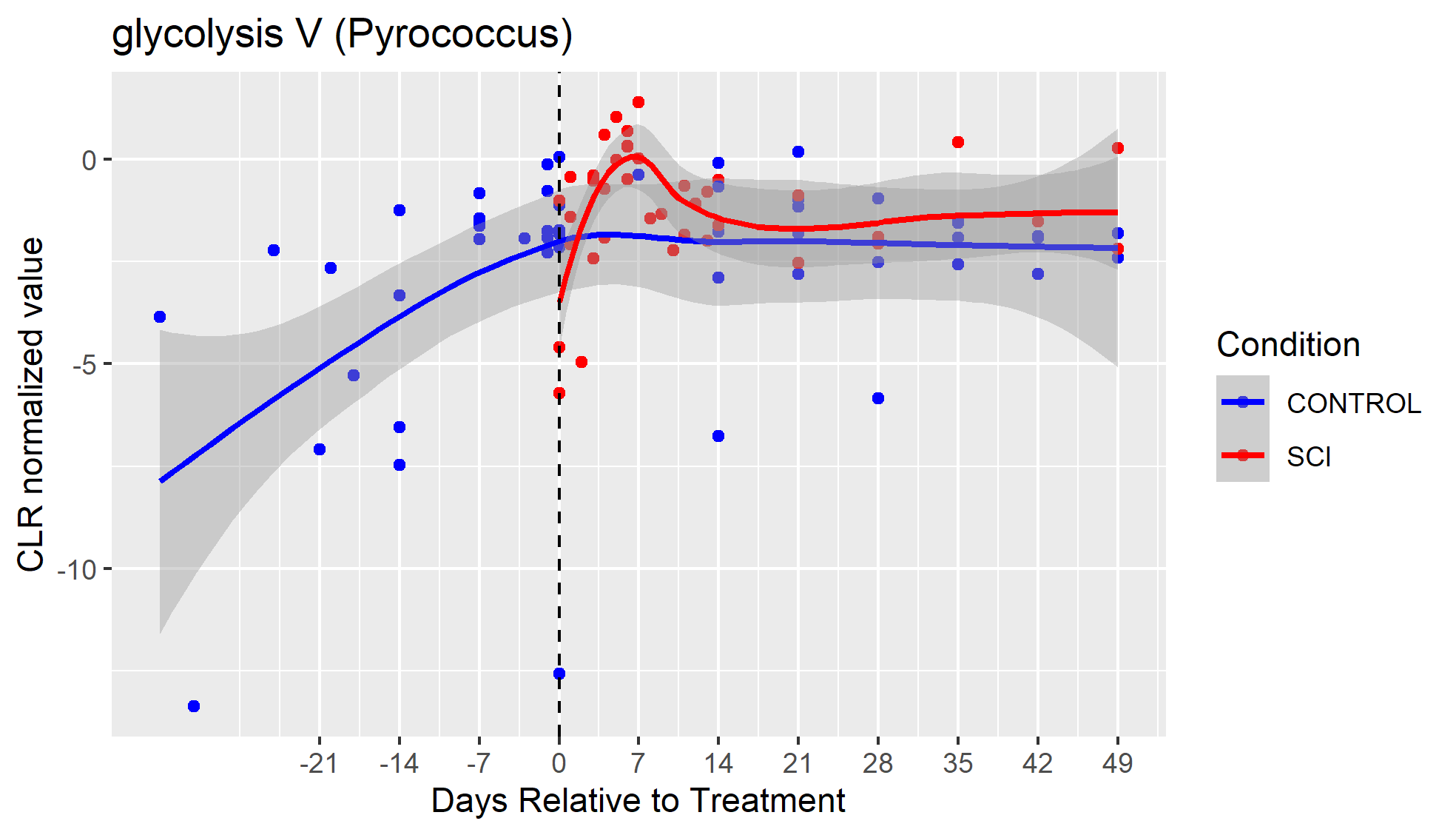

Supplement: Supplementary file 3 — Additional file 3. [file 12864_2021_7979_MOESM3_ESM.zip › pathways_SCI_vs_CONTROL_glycolysis_V_(Pyrococcus).png]

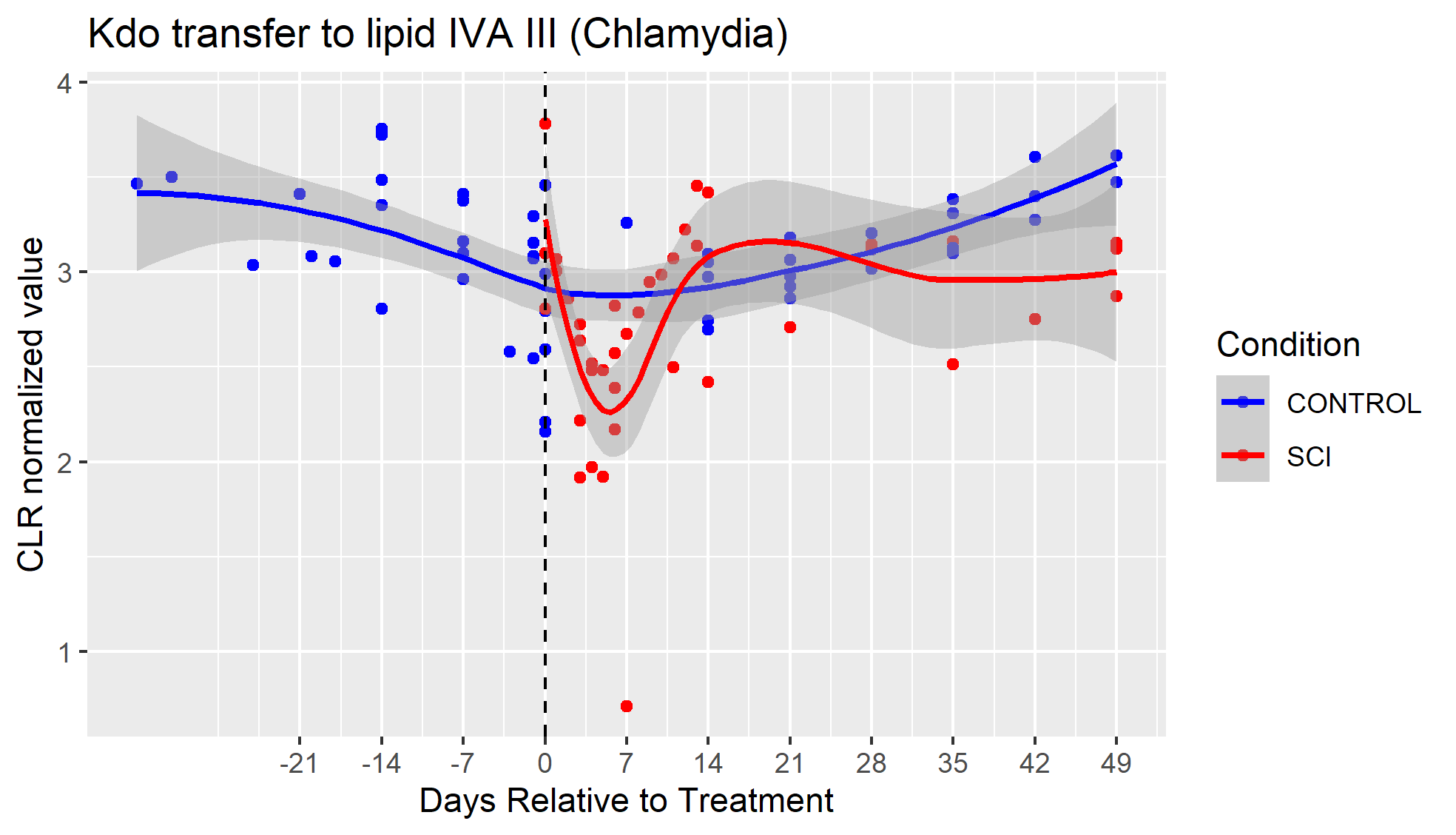

Supplement: Supplementary file 3 — Additional file 3. [file 12864_2021_7979_MOESM3_ESM.zip › pathways_SCI_vs_CONTROL_Kdo_transfer_to_lipid_IVA_III_(Chlamydia).png]

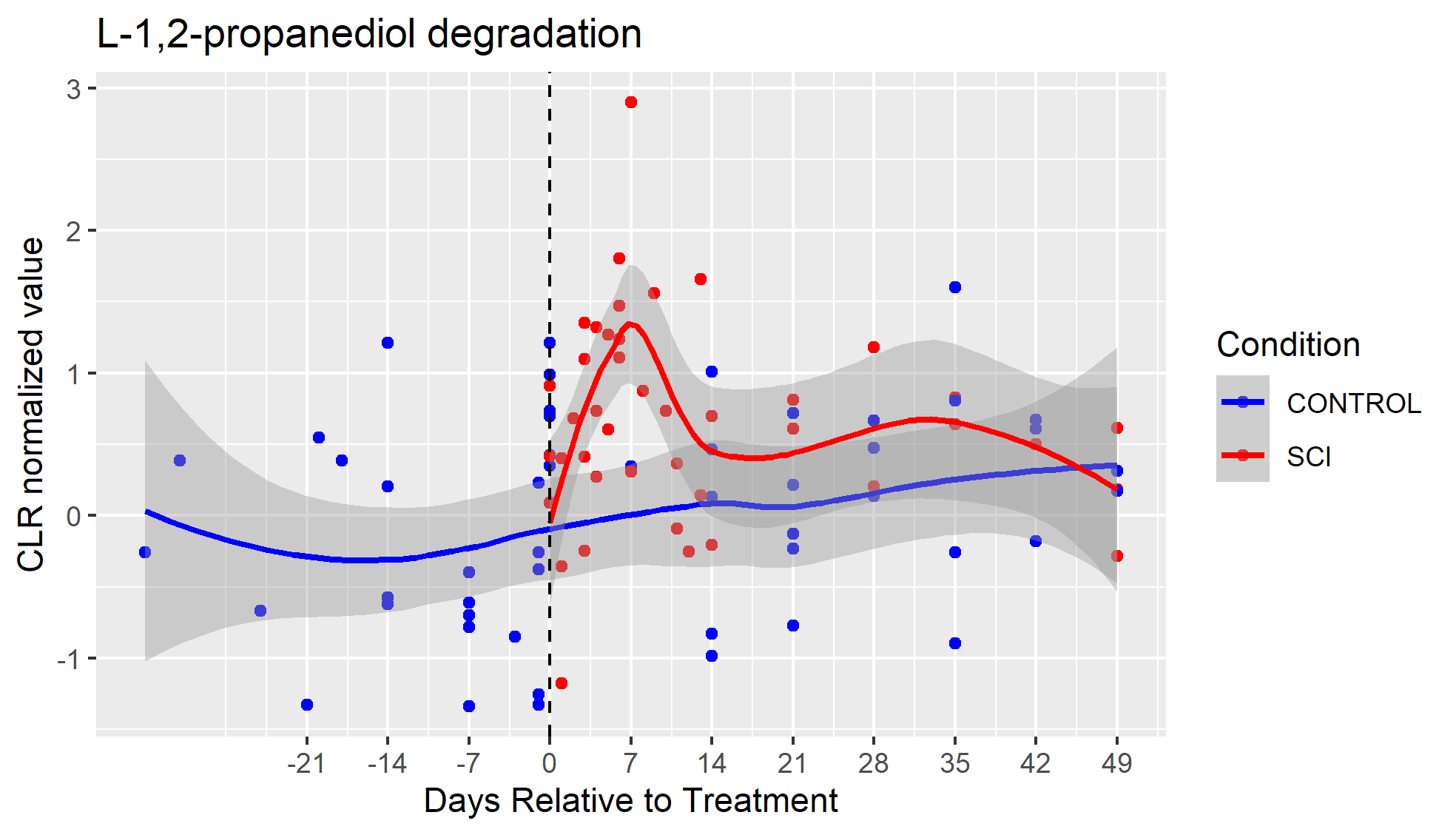

Supplement: Supplementary file 3 — Additional file 3. [file 12864_2021_7979_MOESM3_ESM.zip › pathways_SCI_vs_CONTROL_L-1,2-propanediol_degradation.png]

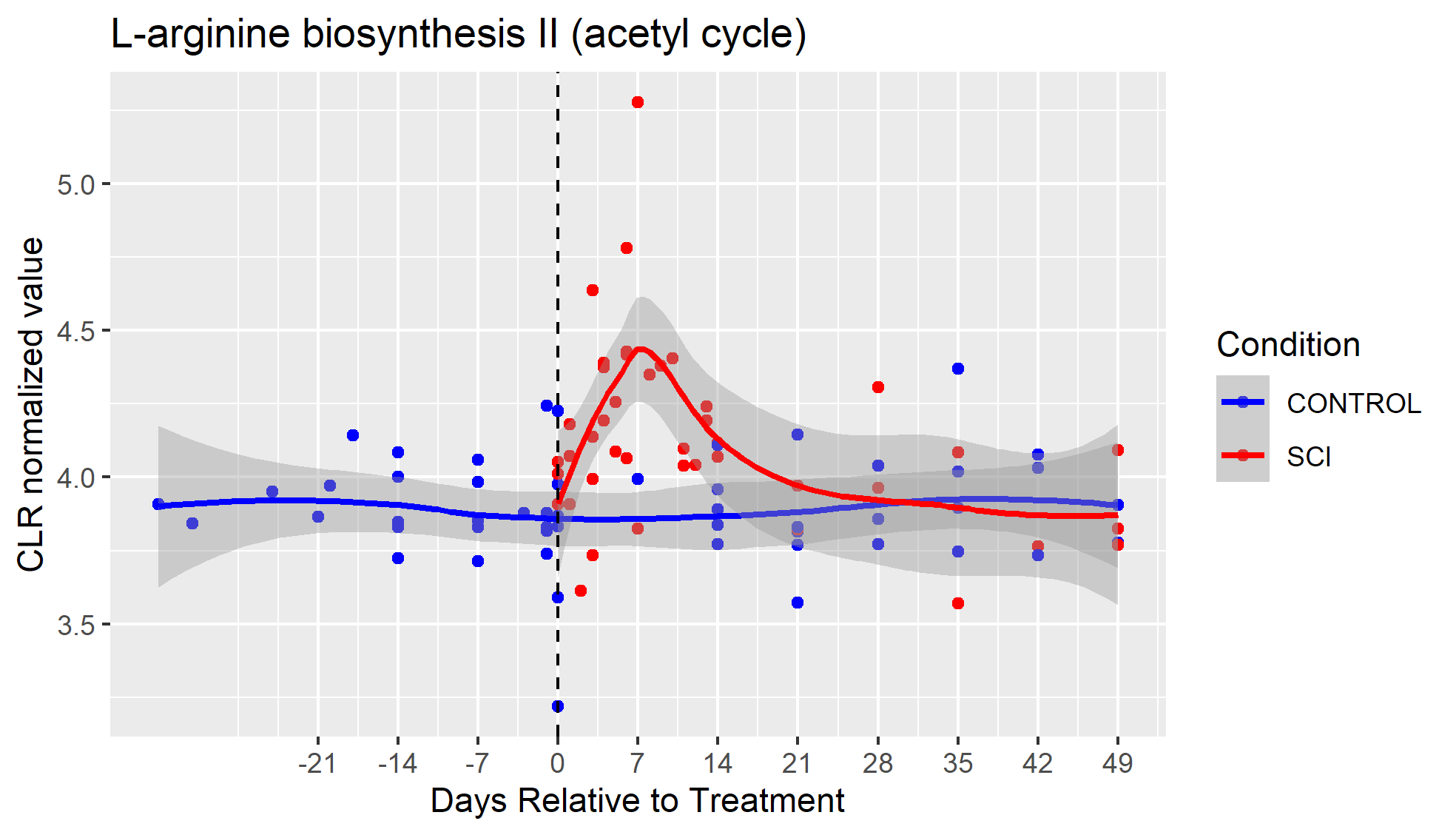

Supplement: Supplementary file 3 — Additional file 3. [file 12864_2021_7979_MOESM3_ESM.zip › pathways_SCI_vs_CONTROL_L-arginine_biosynthesis_II_(acetyl_cycle).png]

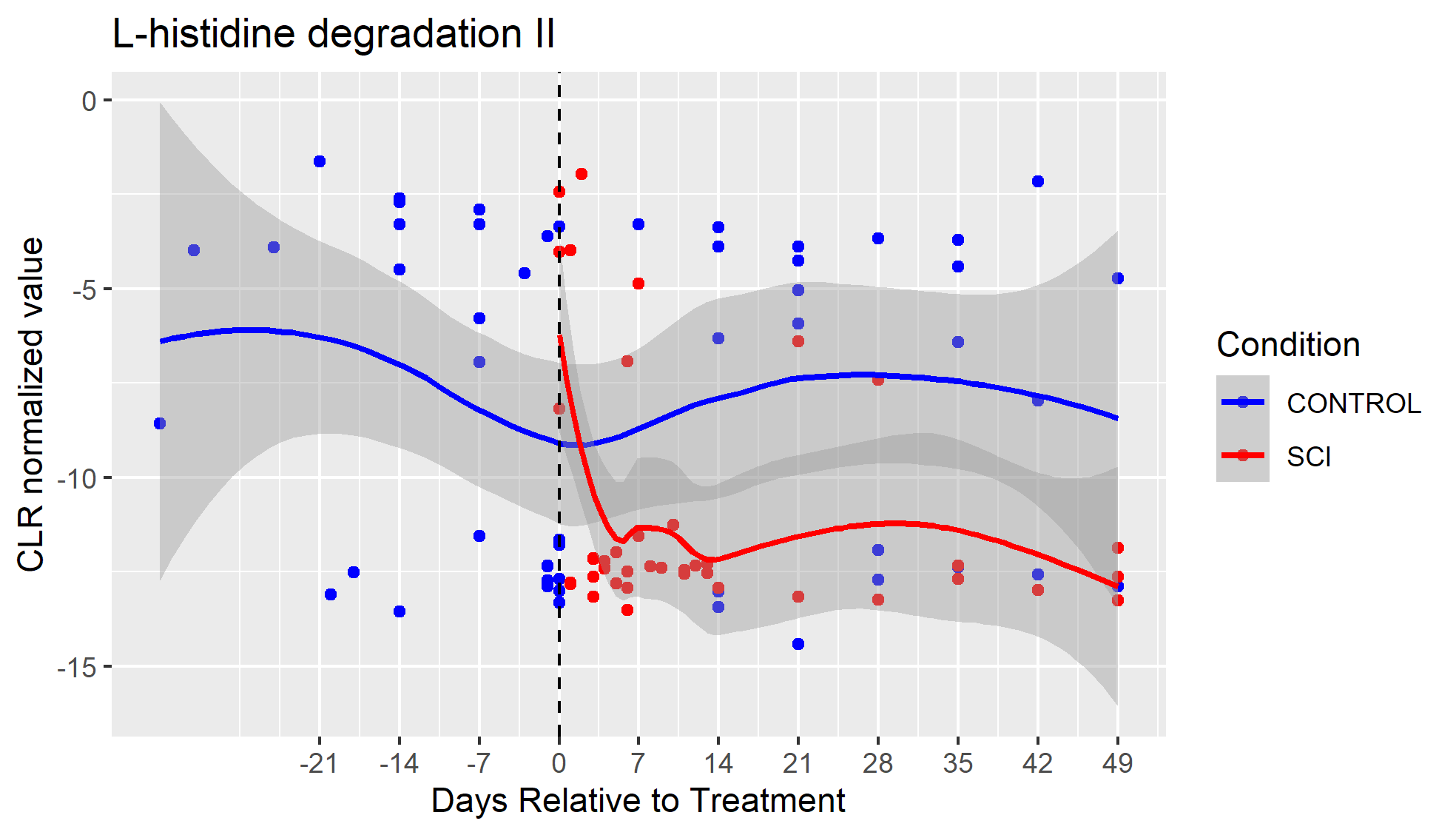

Supplement: Supplementary file 3 — Additional file 3. [file 12864_2021_7979_MOESM3_ESM.zip › pathways_SCI_vs_CONTROL_L-histidine_degradation_II.png]

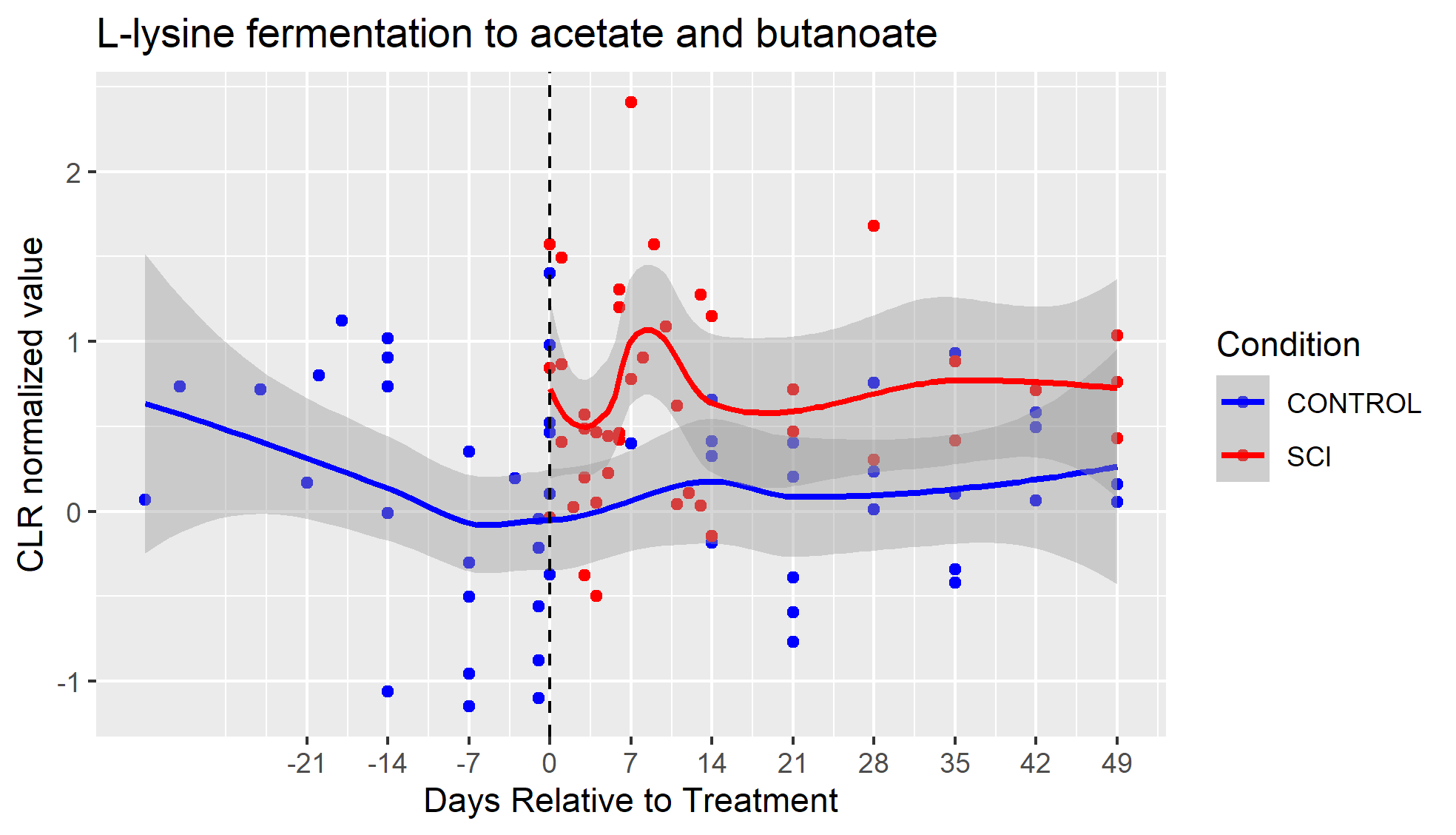

Supplement: Supplementary file 3 — Additional file 3. [file 12864_2021_7979_MOESM3_ESM.zip › pathways_SCI_vs_CONTROL_L-lysine_fermentation_to_acetate_and_butanoate.png]

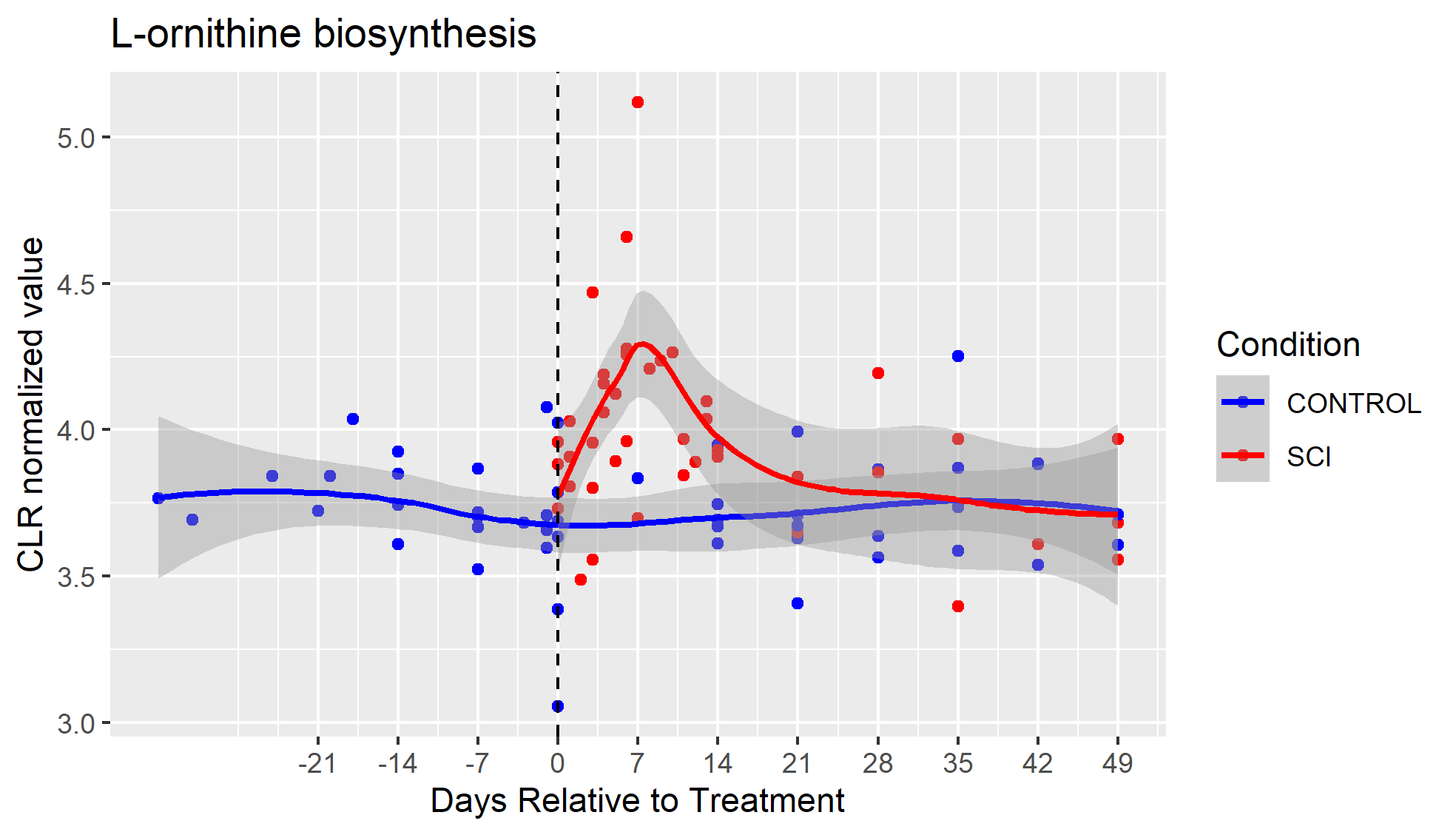

Supplement: Supplementary file 3 — Additional file 3. [file 12864_2021_7979_MOESM3_ESM.zip › pathways_SCI_vs_CONTROL_L-ornithine_biosynthesis.png]

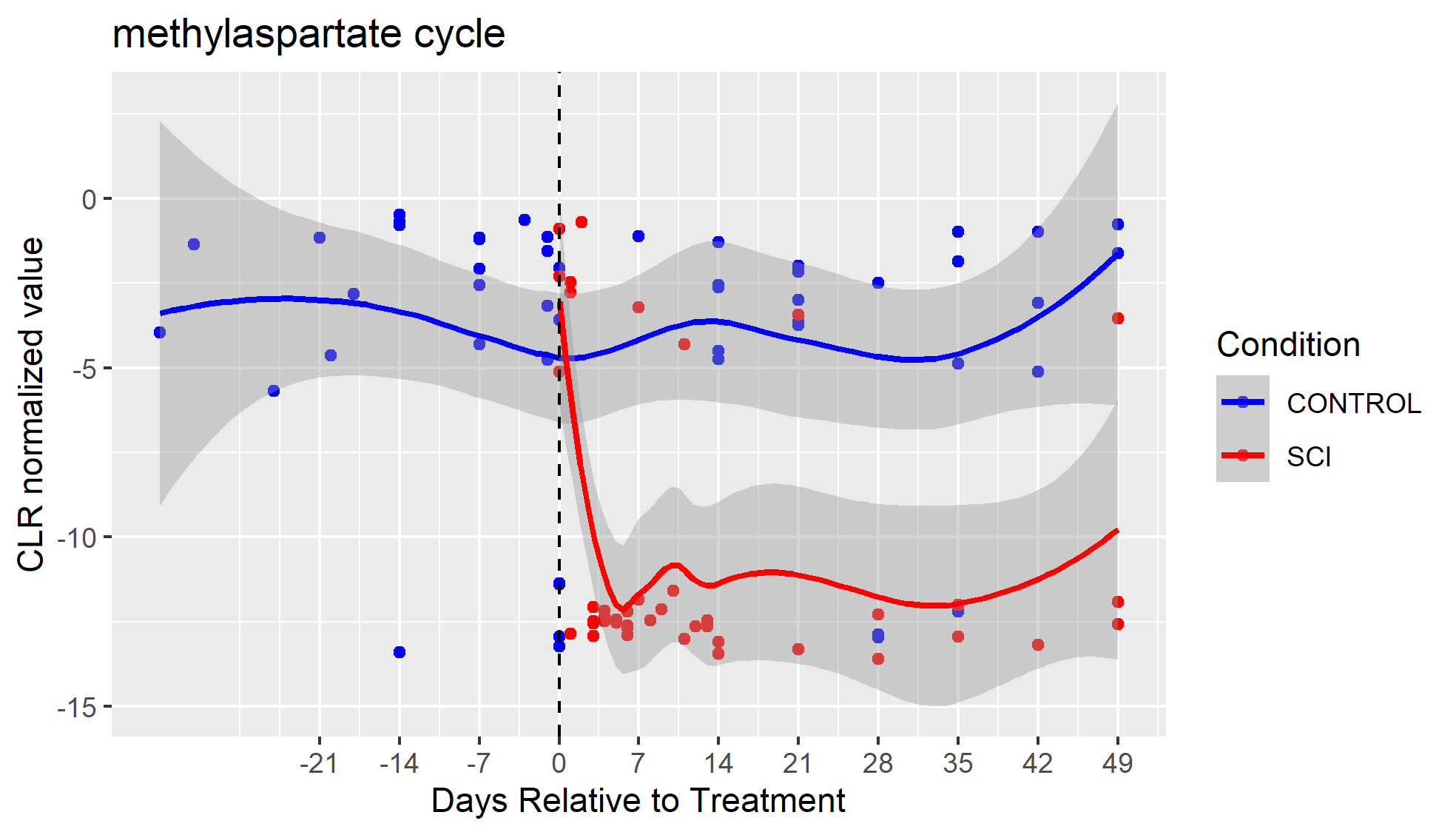

Supplement: Supplementary file 3 — Additional file 3. [file 12864_2021_7979_MOESM3_ESM.zip › pathways_SCI_vs_CONTROL_methylaspartate_cycle.png]

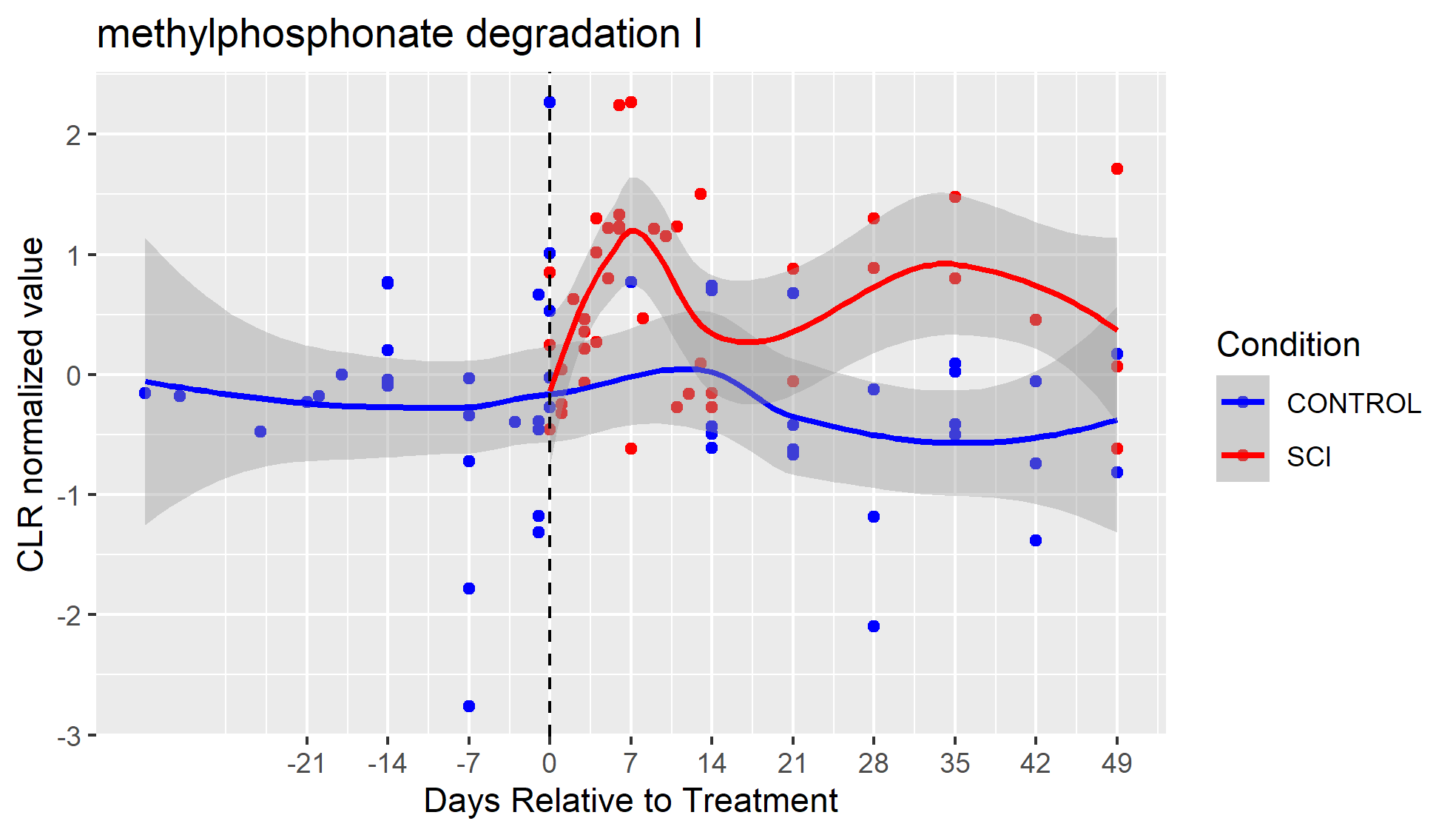

Supplement: Supplementary file 3 — Additional file 3. [file 12864_2021_7979_MOESM3_ESM.zip › pathways_SCI_vs_CONTROL_methylphosphonate_degradation_I.png]

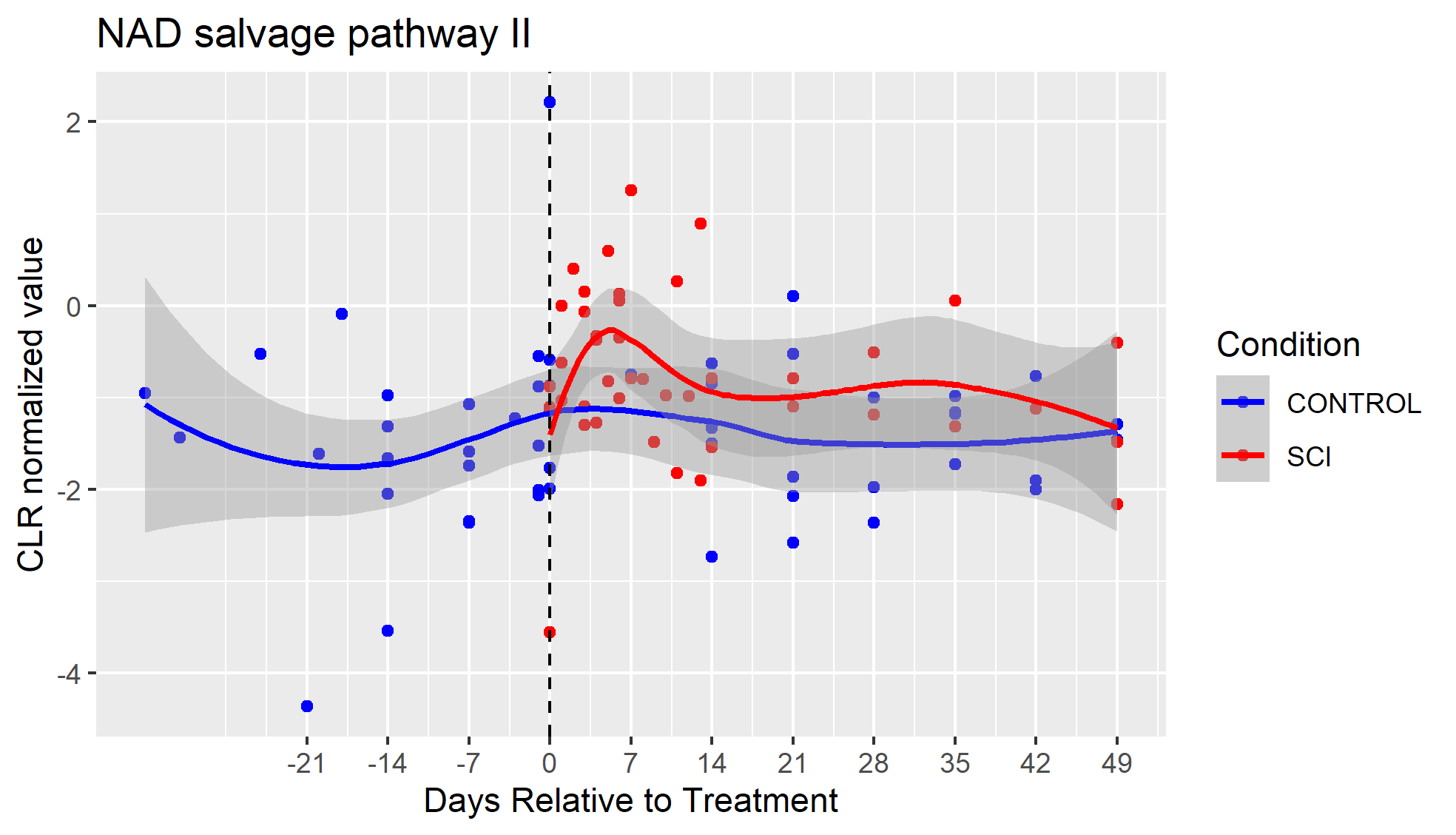

Supplement: Supplementary file 3 — Additional file 3. [file 12864_2021_7979_MOESM3_ESM.zip › pathways_SCI_vs_CONTROL_NAD_salvage_pathway_II.png]

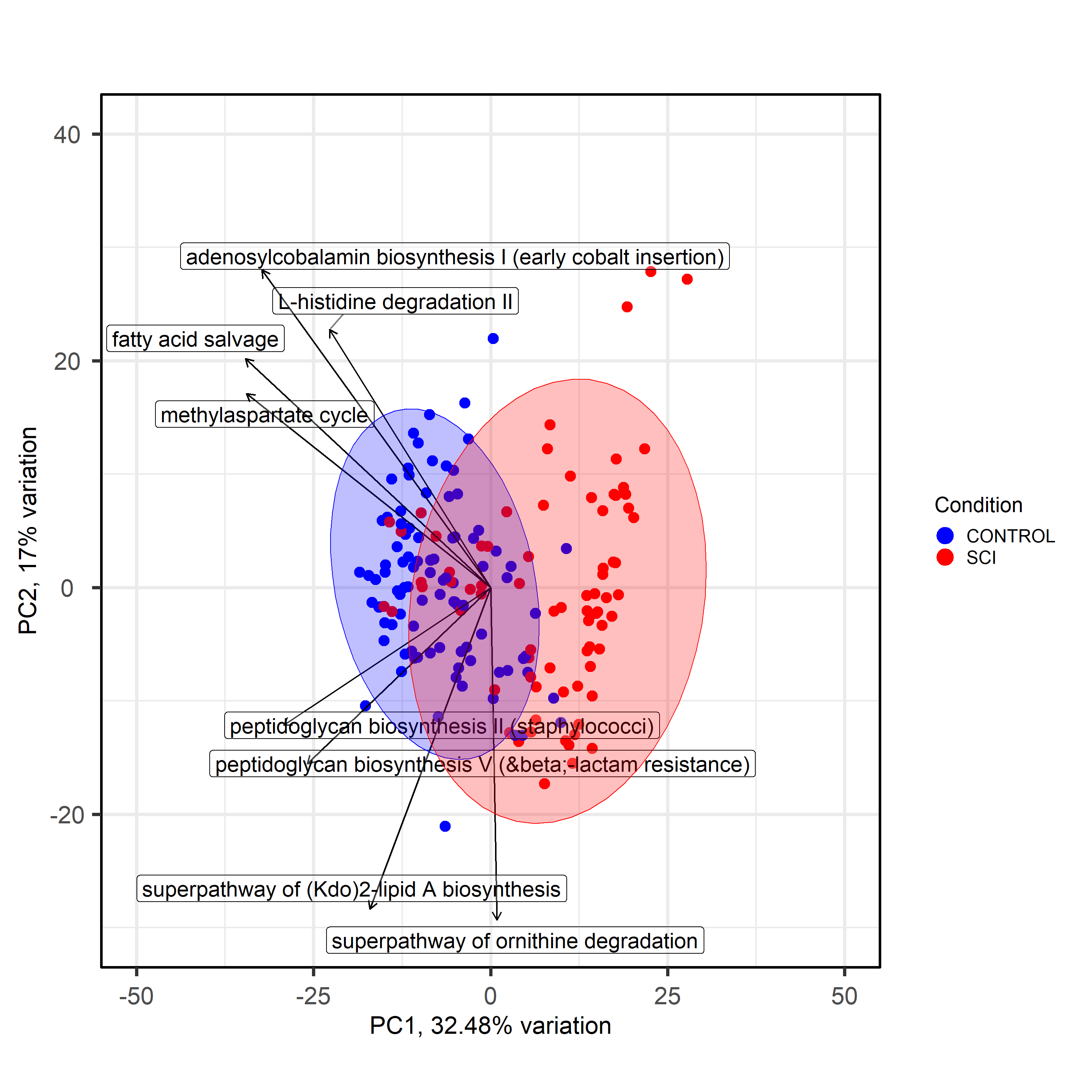

Supplement: Supplementary file 3 — Additional file 3. [file 12864_2021_7979_MOESM3_ESM.zip › pathways_SCI_vs_CONTROL_PCA_PC1_PC2.png]

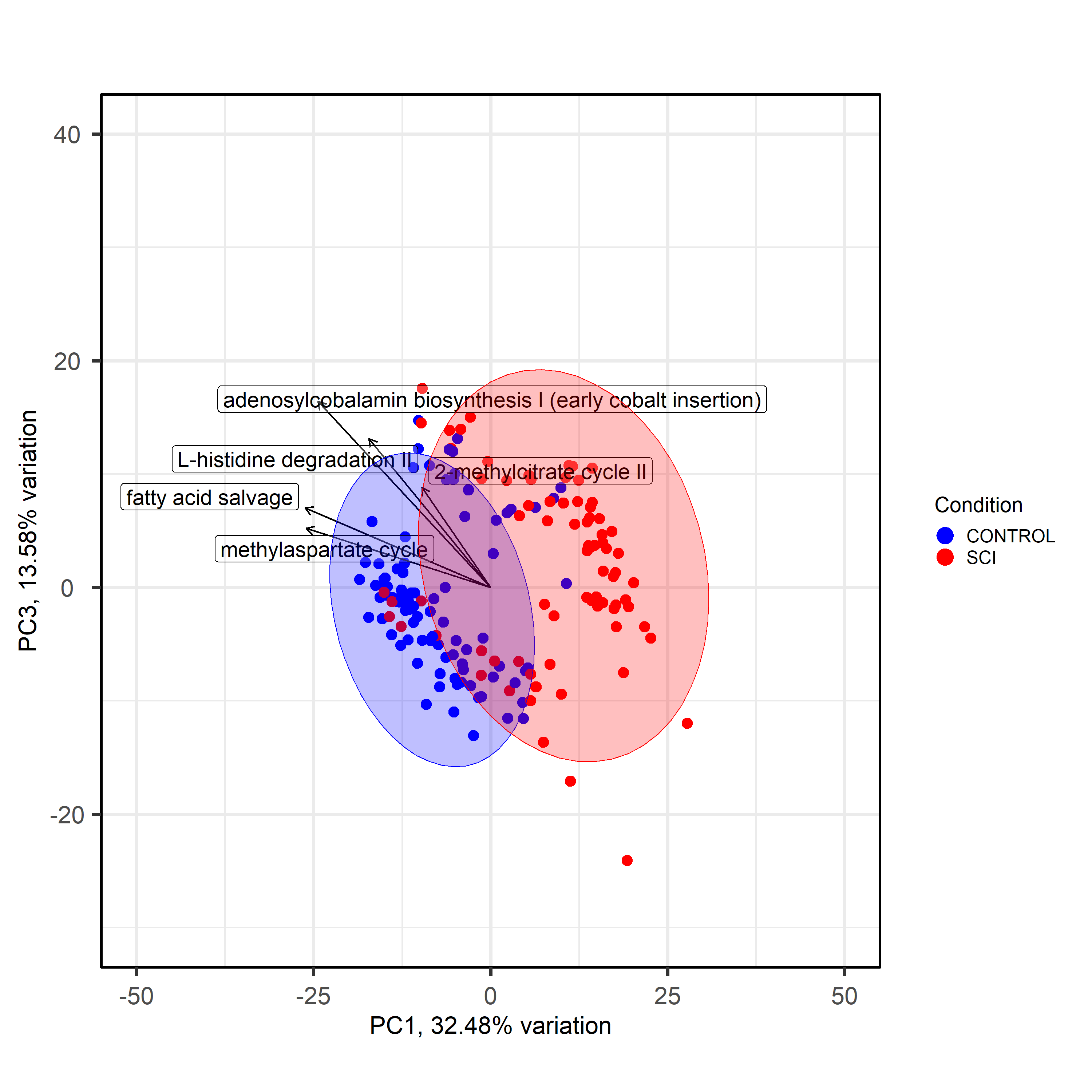

Supplement: Supplementary file 3 — Additional file 3. [file 12864_2021_7979_MOESM3_ESM.zip › pathways_SCI_vs_CONTROL_PCA_PC1_PC3.png]

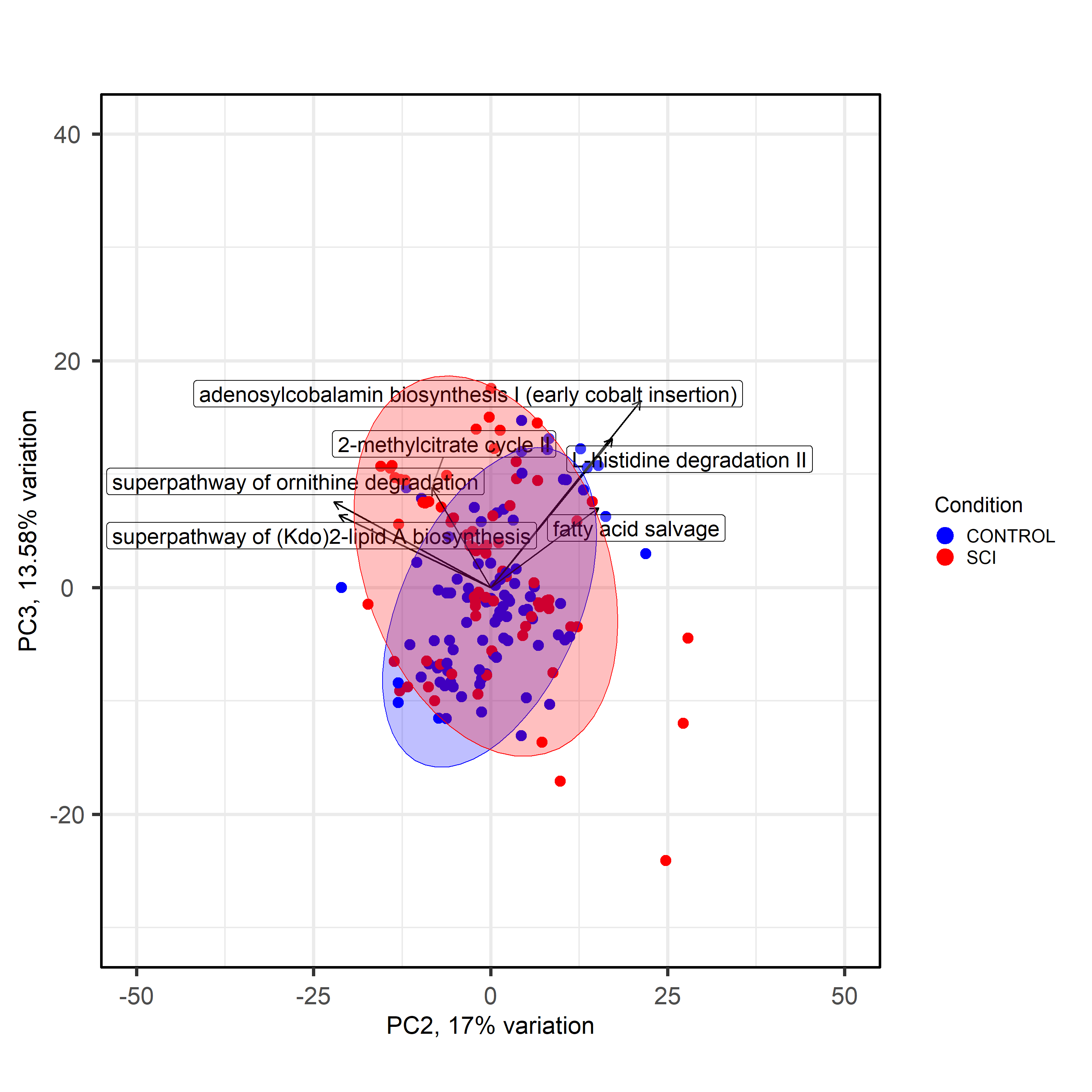

Supplement: Supplementary file 3 — Additional file 3. [file 12864_2021_7979_MOESM3_ESM.zip › pathways_SCI_vs_CONTROL_PCA_PC2_PC3.png]

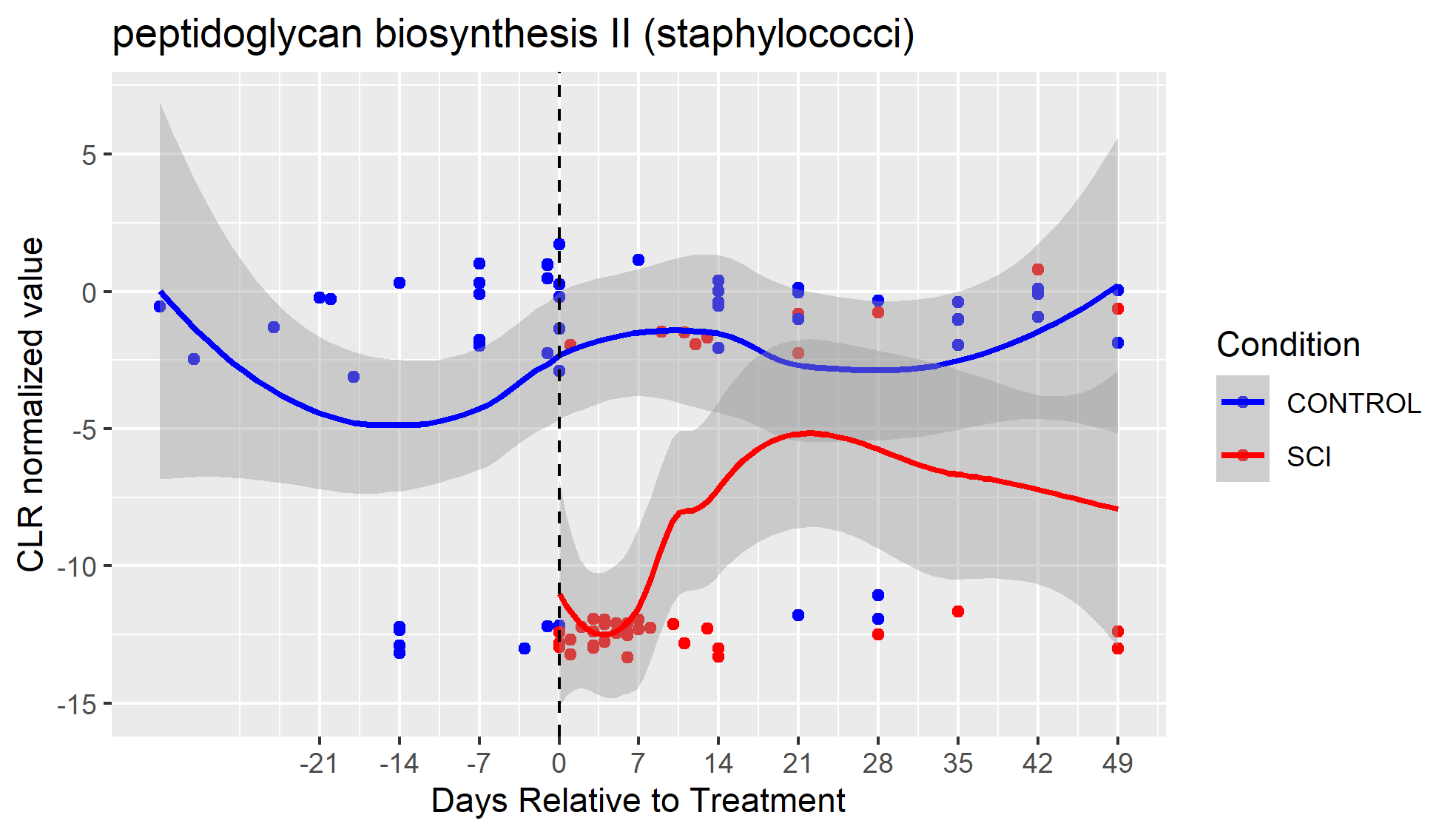

Supplement: Supplementary file 3 — Additional file 3. [file 12864_2021_7979_MOESM3_ESM.zip › pathways_SCI_vs_CONTROL_peptidoglycan_biosynthesis_II_(staphylococci).png]

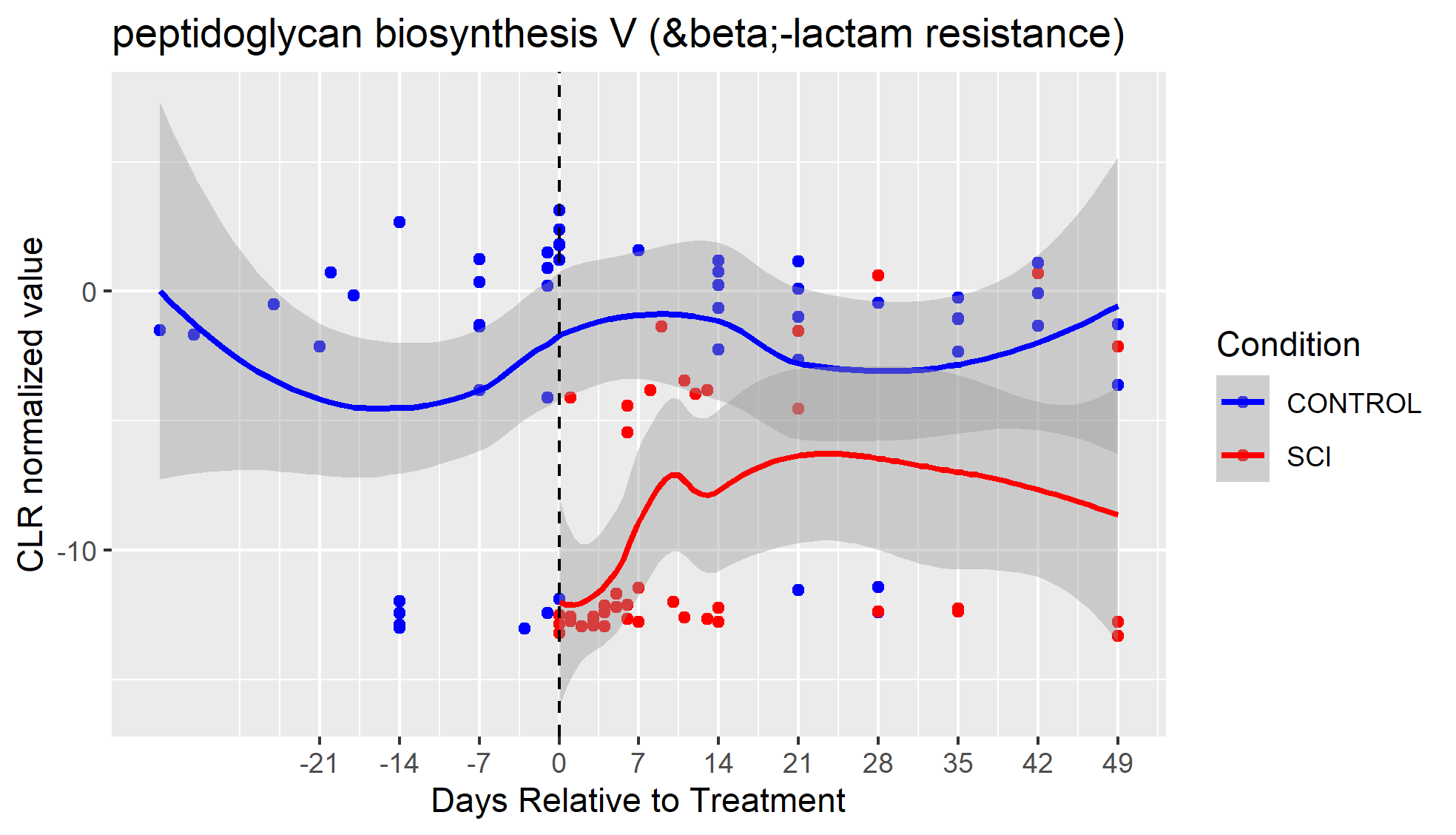

Supplement: Supplementary file 3 — Additional file 3. [file 12864_2021_7979_MOESM3_ESM.zip › pathways_SCI_vs_CONTROL_peptidoglycan_biosynthesis_V_(β-lactam_resistance).png]

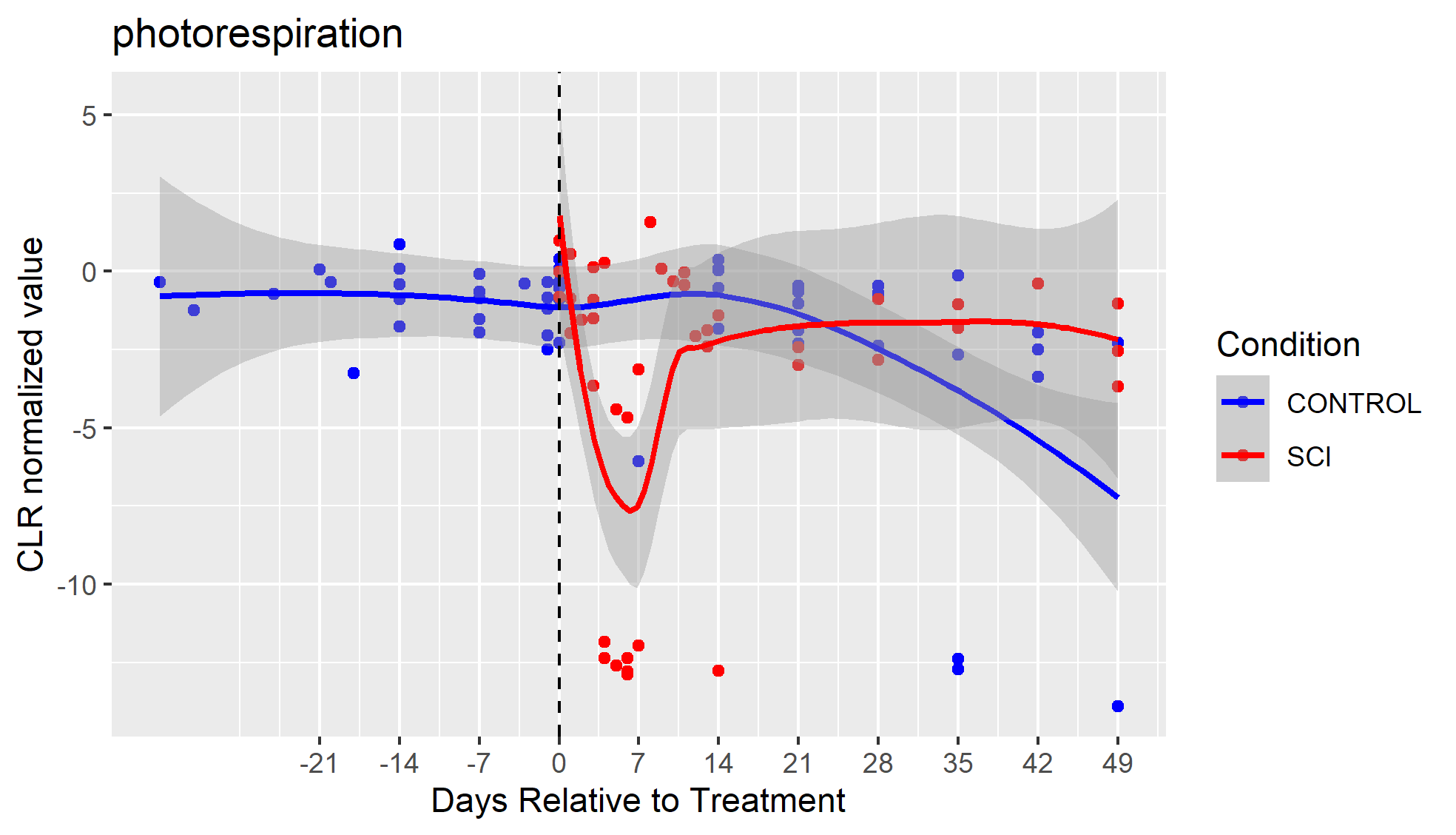

Supplement: Supplementary file 3 — Additional file 3. [file 12864_2021_7979_MOESM3_ESM.zip › pathways_SCI_vs_CONTROL_photorespiration.png]

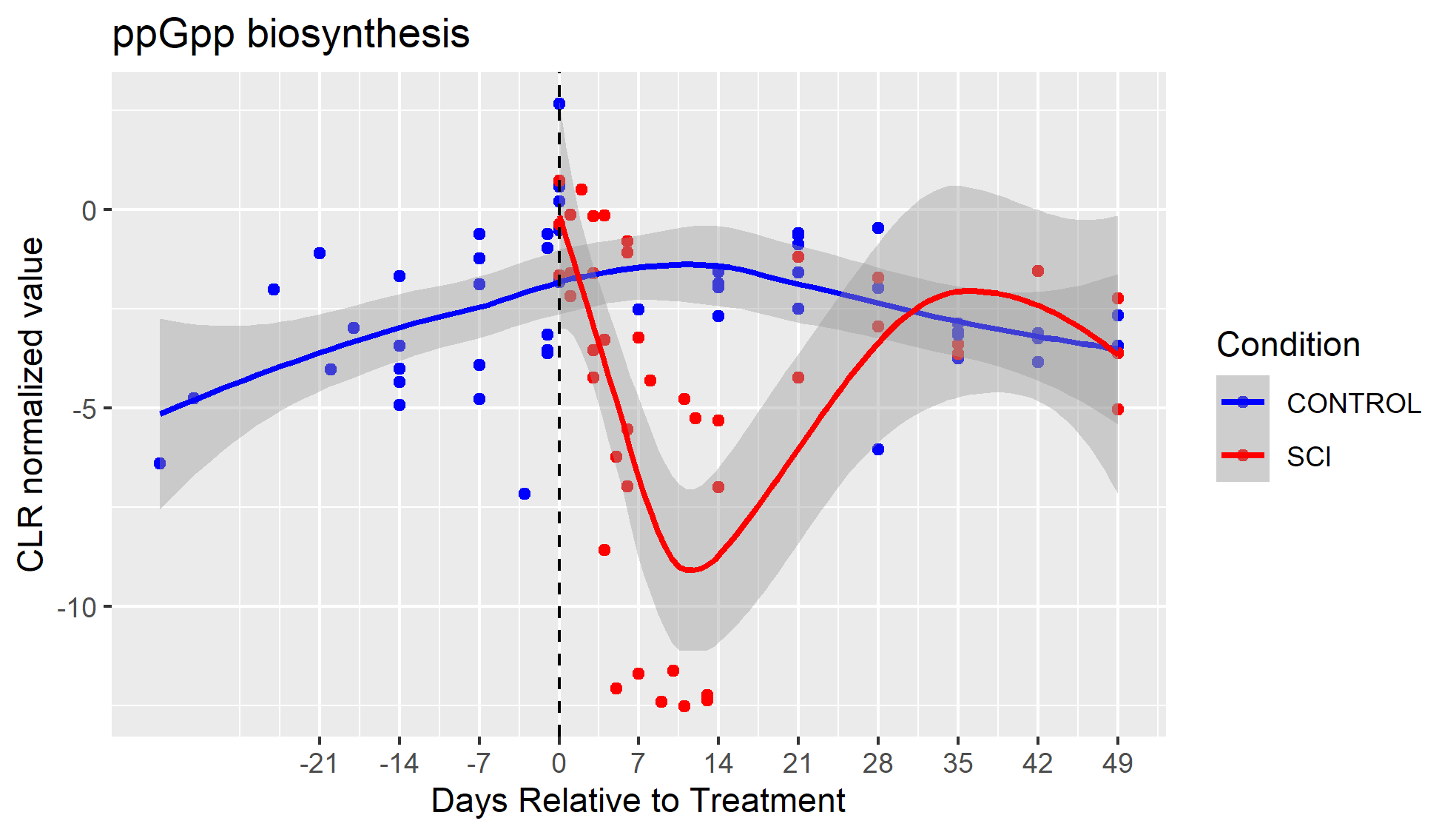

Supplement: Supplementary file 3 — Additional file 3. [file 12864_2021_7979_MOESM3_ESM.zip › pathways_SCI_vs_CONTROL_ppGpp_biosynthesis.png]

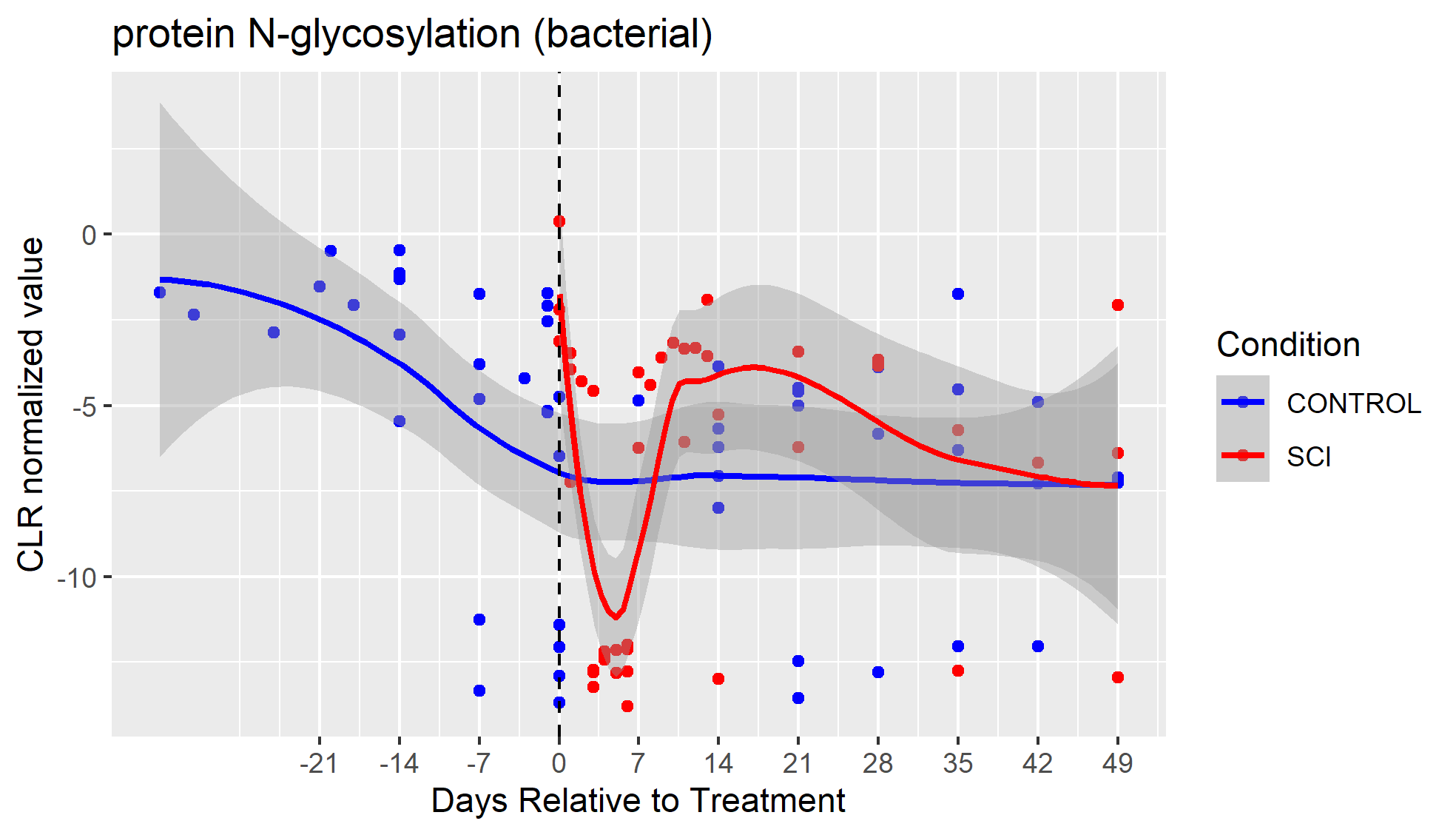

Supplement: Supplementary file 3 — Additional file 3. [file 12864_2021_7979_MOESM3_ESM.zip › pathways_SCI_vs_CONTROL_protein_N-glycosylation_(bacterial).png]

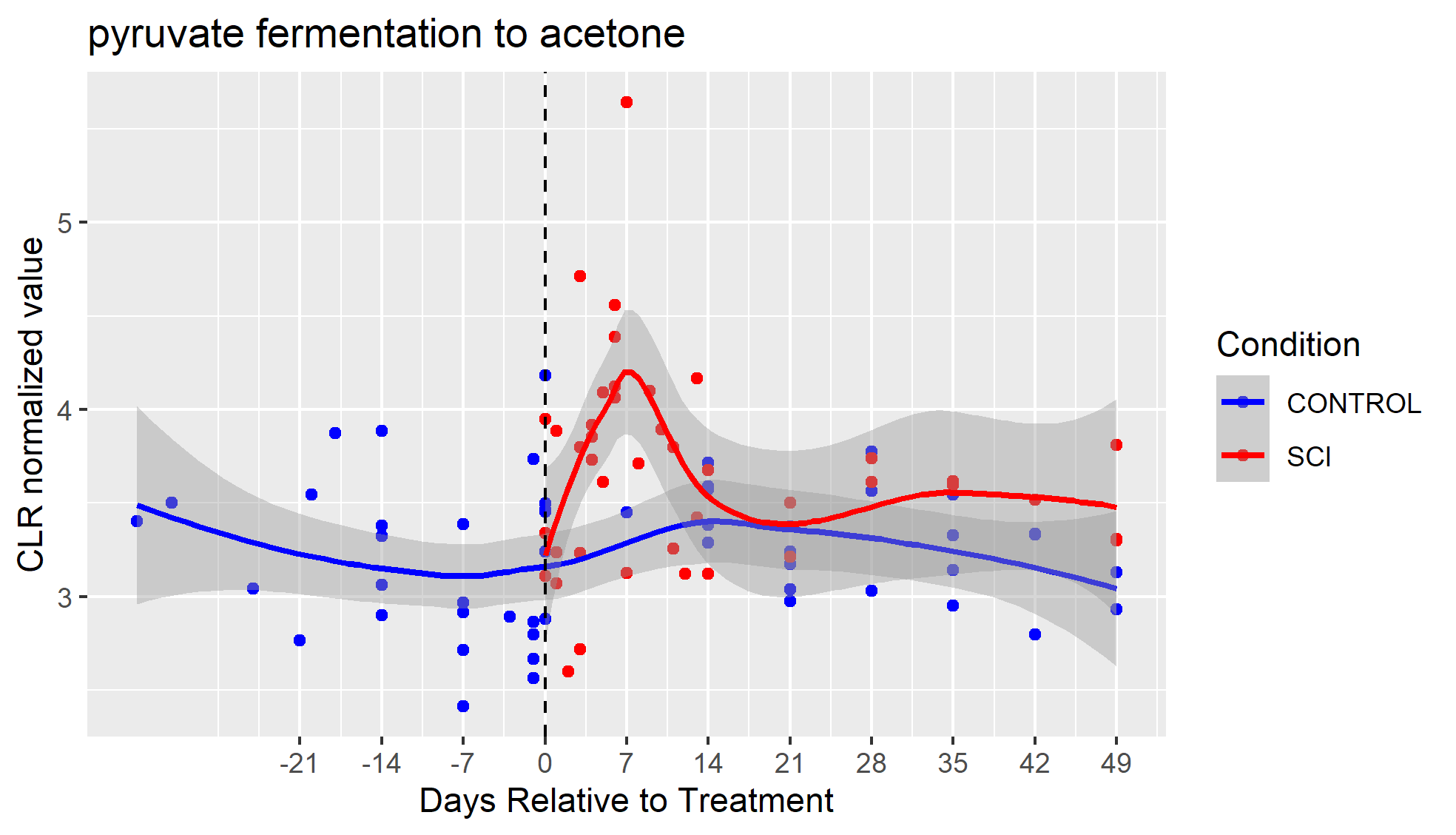

Supplement: Supplementary file 3 — Additional file 3. [file 12864_2021_7979_MOESM3_ESM.zip › pathways_SCI_vs_CONTROL_pyruvate_fermentation_to_acetone.png]

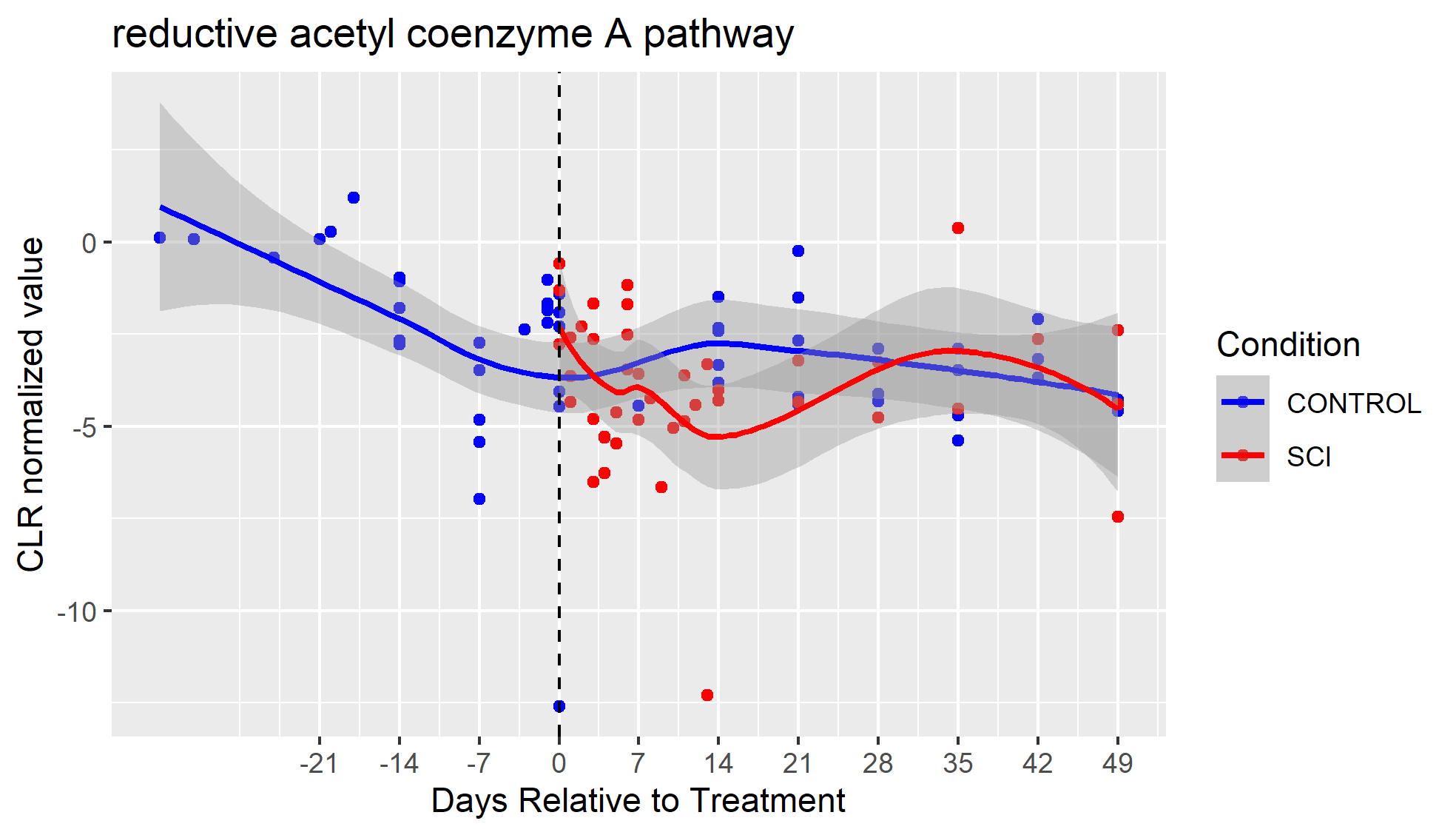

Supplement: Supplementary file 3 — Additional file 3. [file 12864_2021_7979_MOESM3_ESM.zip › pathways_SCI_vs_CONTROL_reductive_acetyl_coenzyme_A_pathway.png]

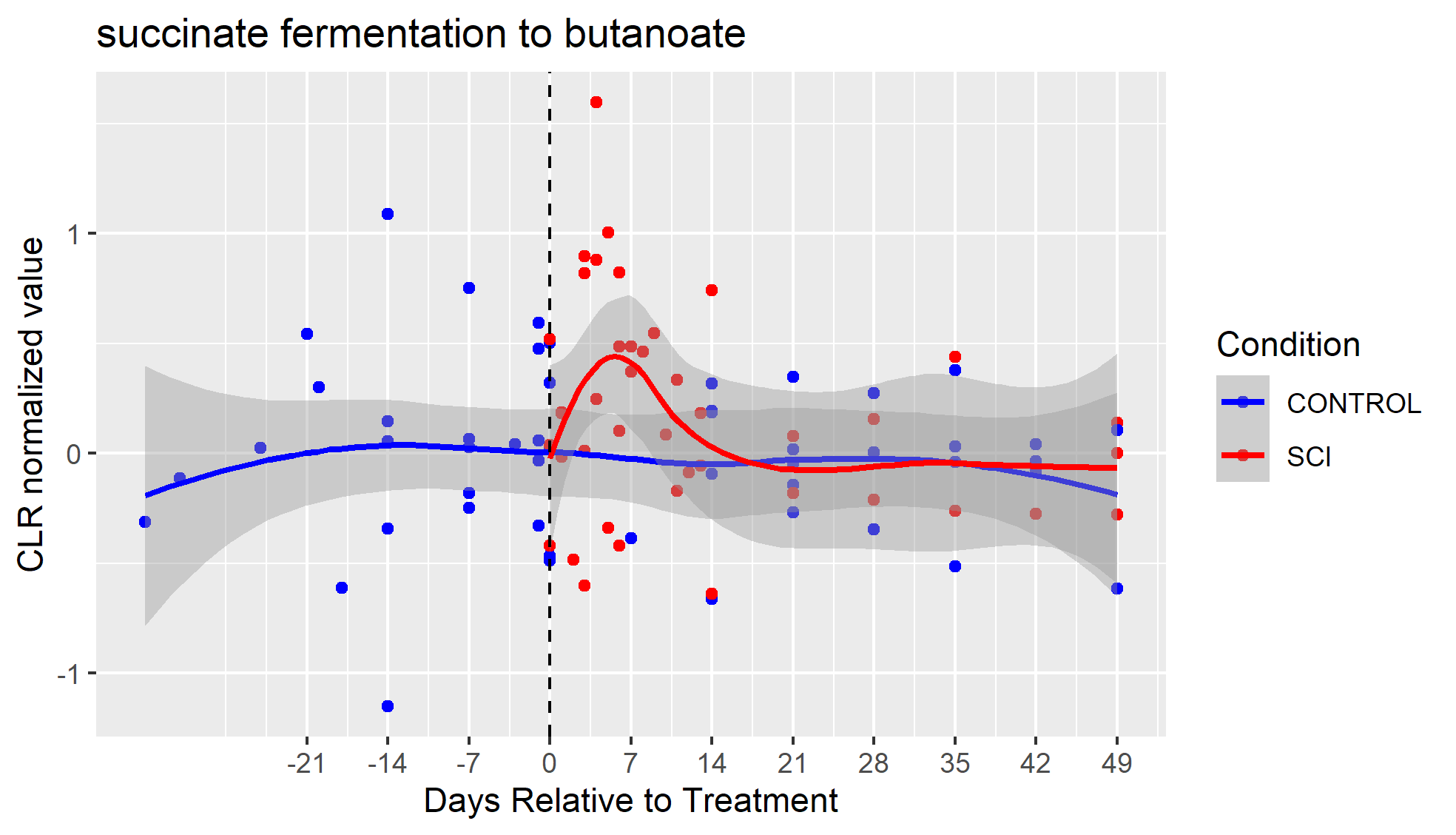

Supplement: Supplementary file 3 — Additional file 3. [file 12864_2021_7979_MOESM3_ESM.zip › pathways_SCI_vs_CONTROL_succinate_fermentation_to_butanoate.png]

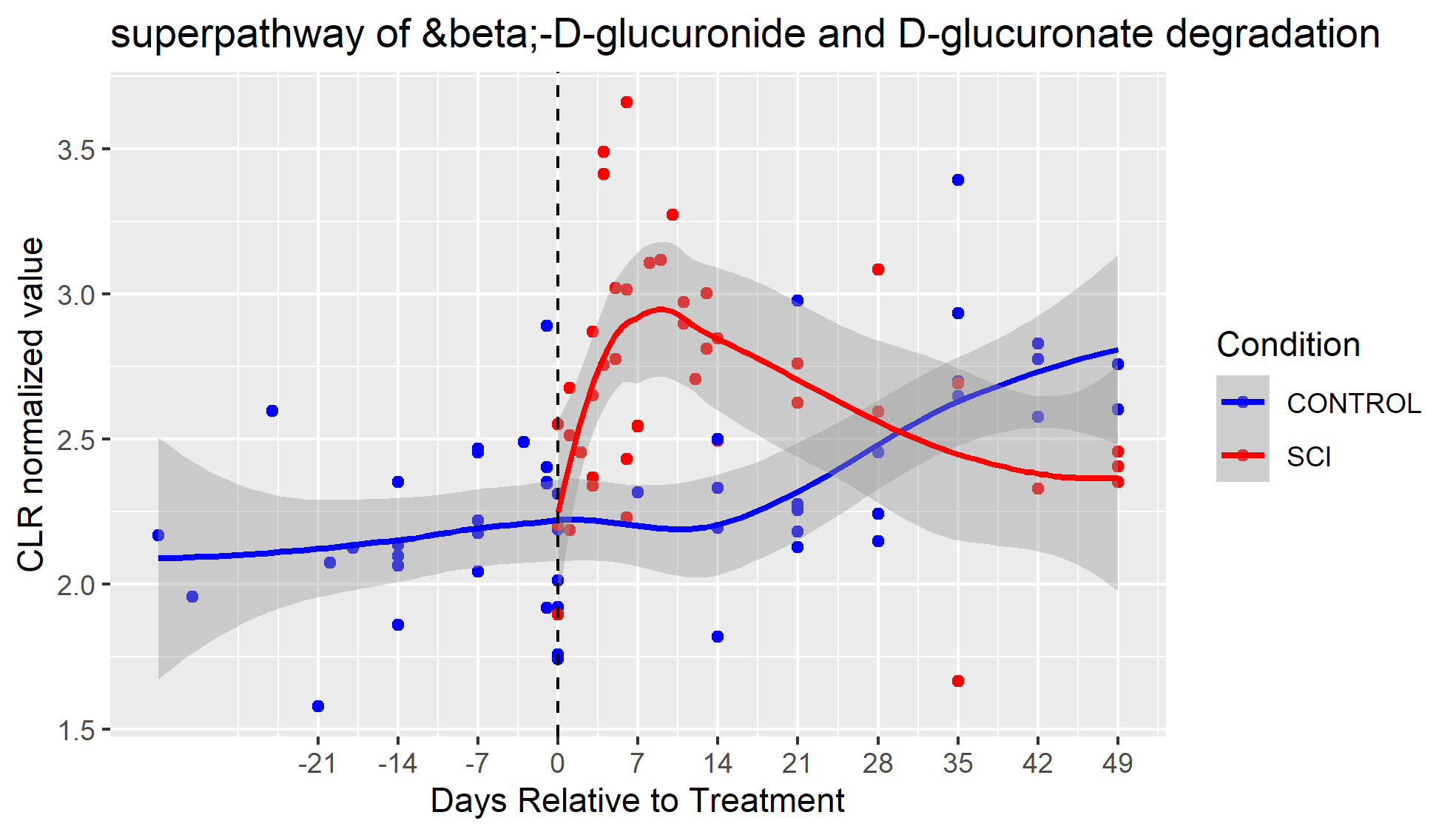

Supplement: Supplementary file 3 — Additional file 3. [file 12864_2021_7979_MOESM3_ESM.zip › pathways_SCI_vs_CONTROL_superpathway_of_β-D-glucuronide_and_D-glucuronate_degradation.png]

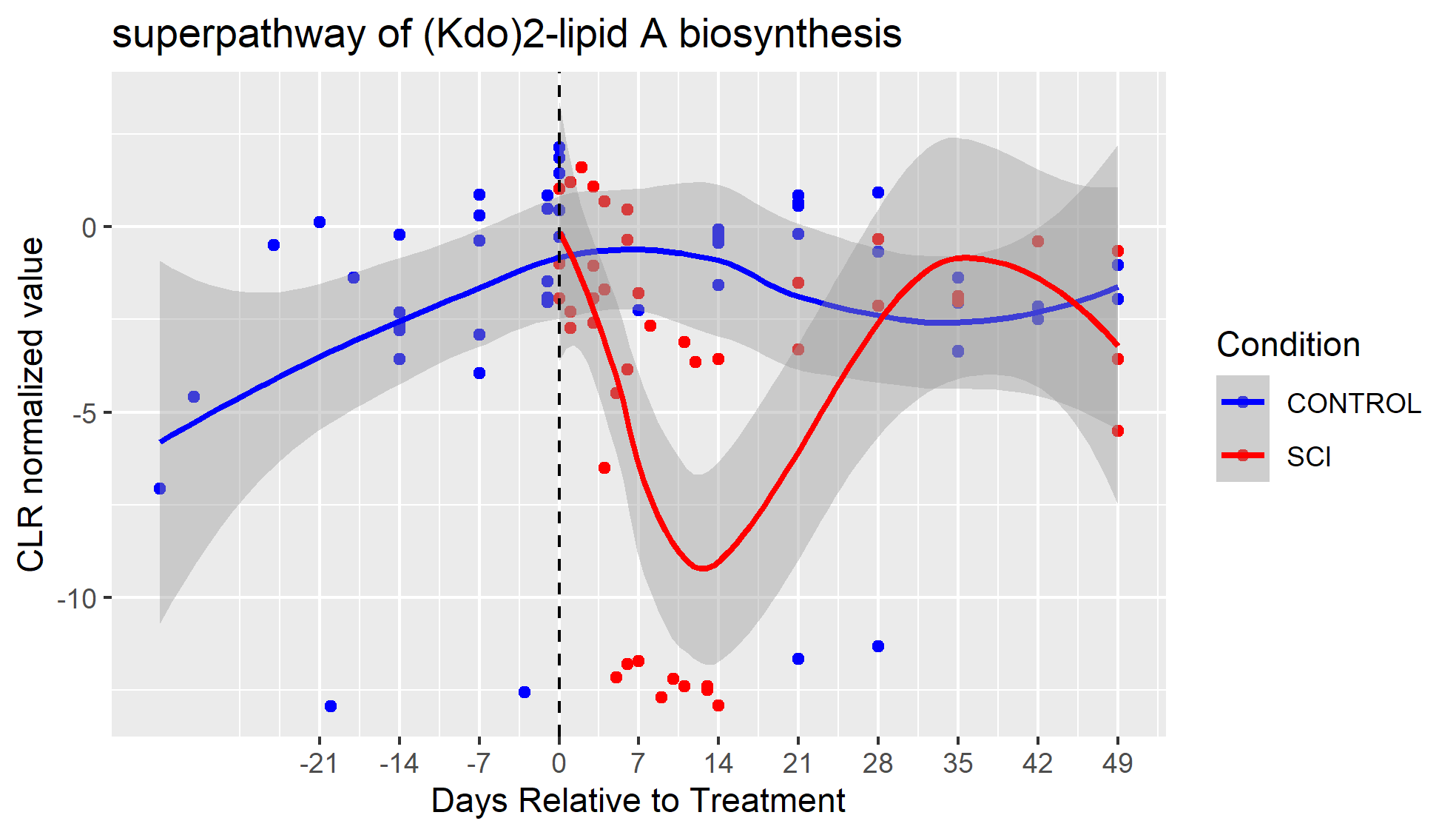

Supplement: Supplementary file 3 — Additional file 3. [file 12864_2021_7979_MOESM3_ESM.zip › pathways_SCI_vs_CONTROL_superpathway_of_(Kdo)2-lipid_A_biosynthesis.png]

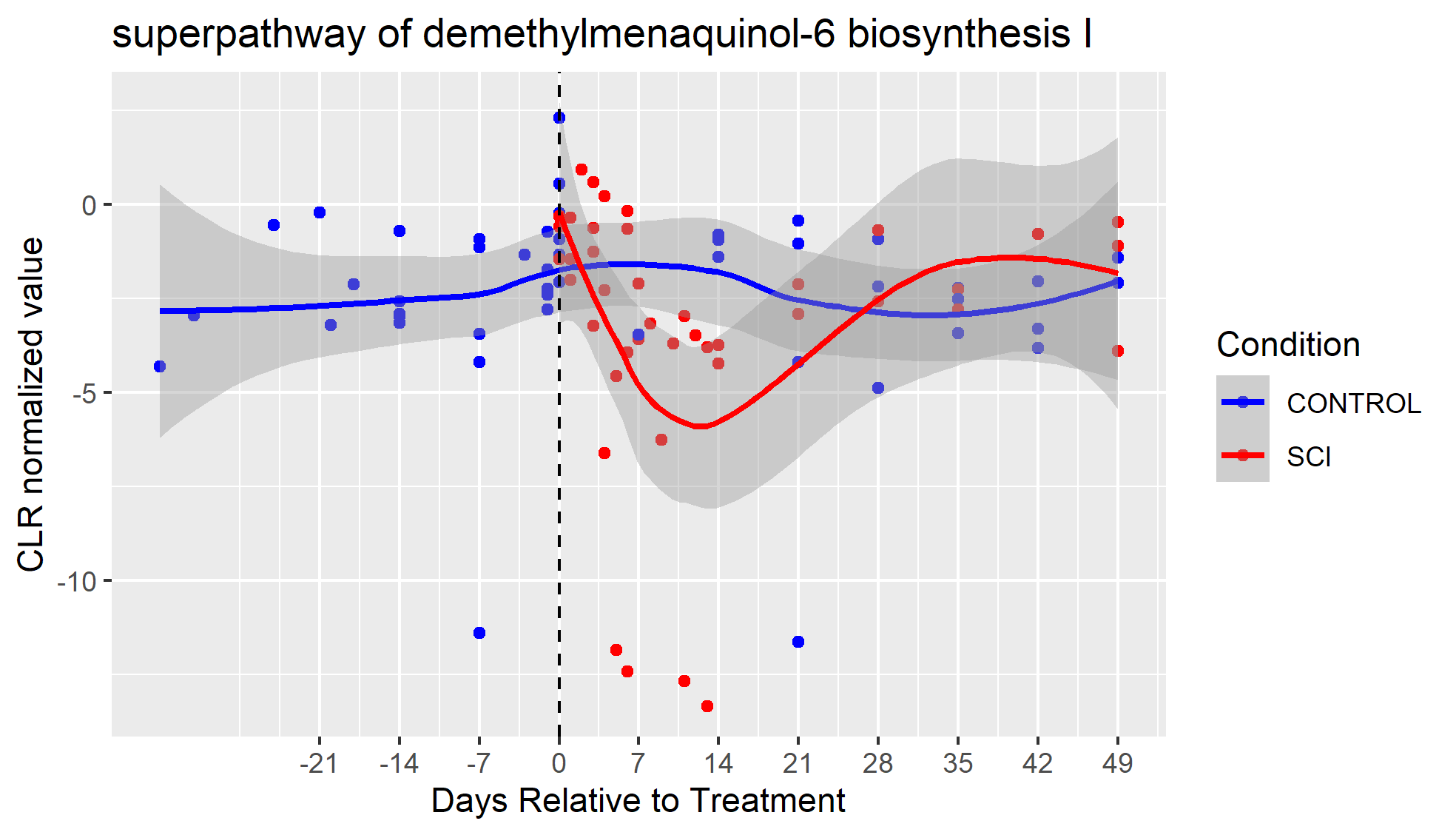

Supplement: Supplementary file 3 — Additional file 3. [file 12864_2021_7979_MOESM3_ESM.zip › pathways_SCI_vs_CONTROL_superpathway_of_demethylmenaquinol-6_biosynthesis_I.png]

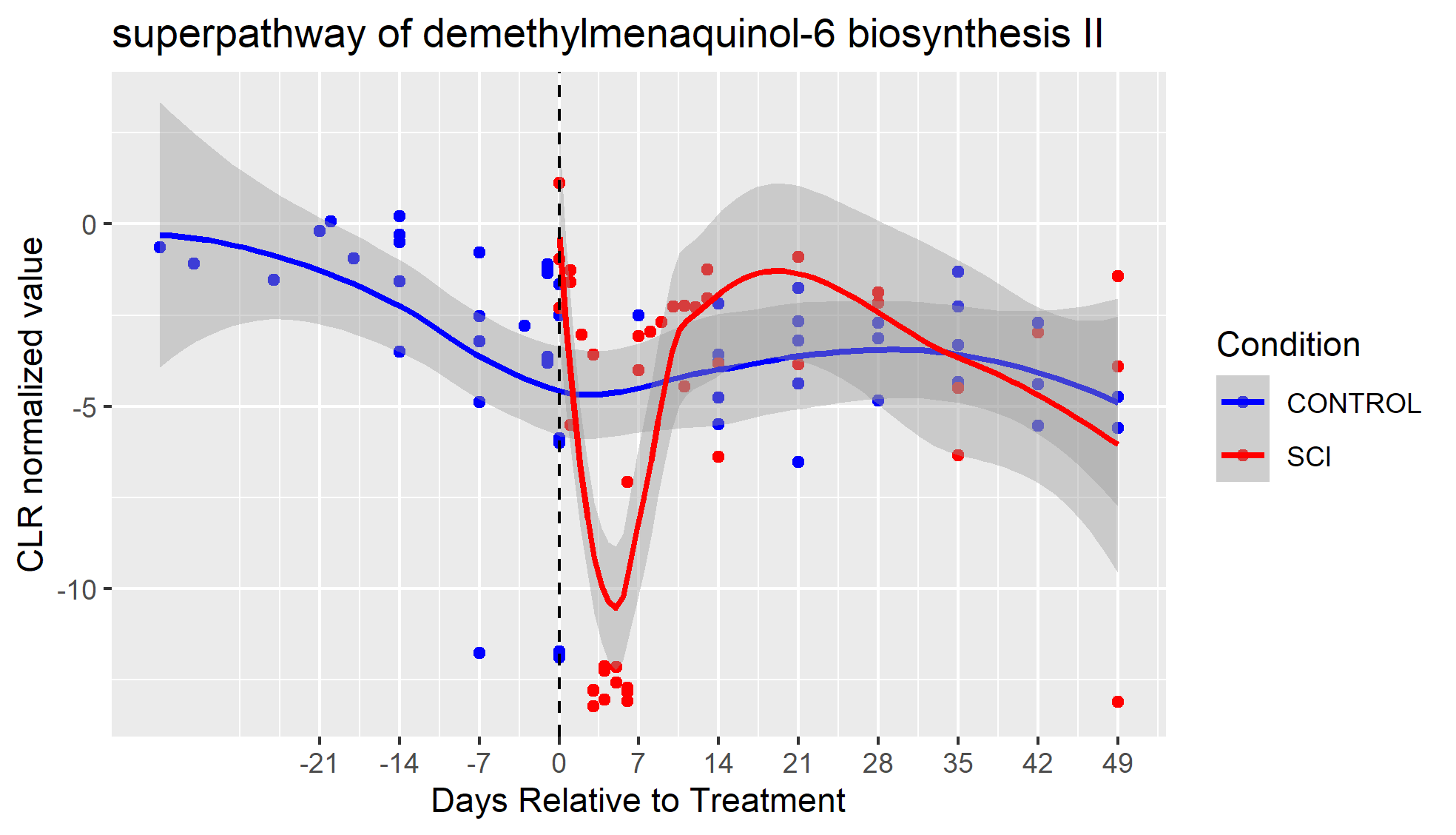

Supplement: Supplementary file 3 — Additional file 3. [file 12864_2021_7979_MOESM3_ESM.zip › pathways_SCI_vs_CONTROL_superpathway_of_demethylmenaquinol-6_biosynthesis_II.png]

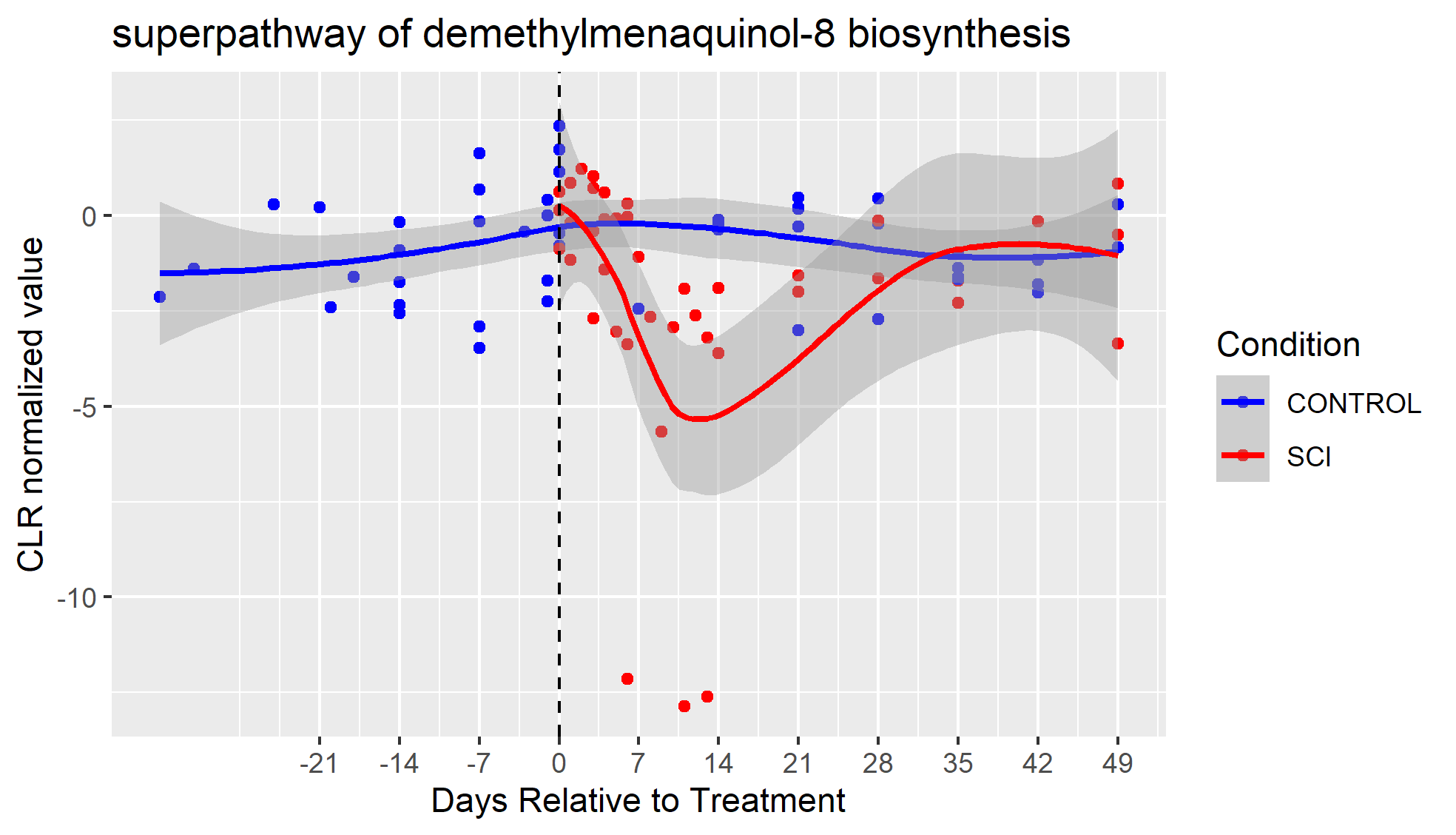

Supplement: Supplementary file 3 — Additional file 3. [file 12864_2021_7979_MOESM3_ESM.zip › pathways_SCI_vs_CONTROL_superpathway_of_demethylmenaquinol-8_biosynthesis.png]

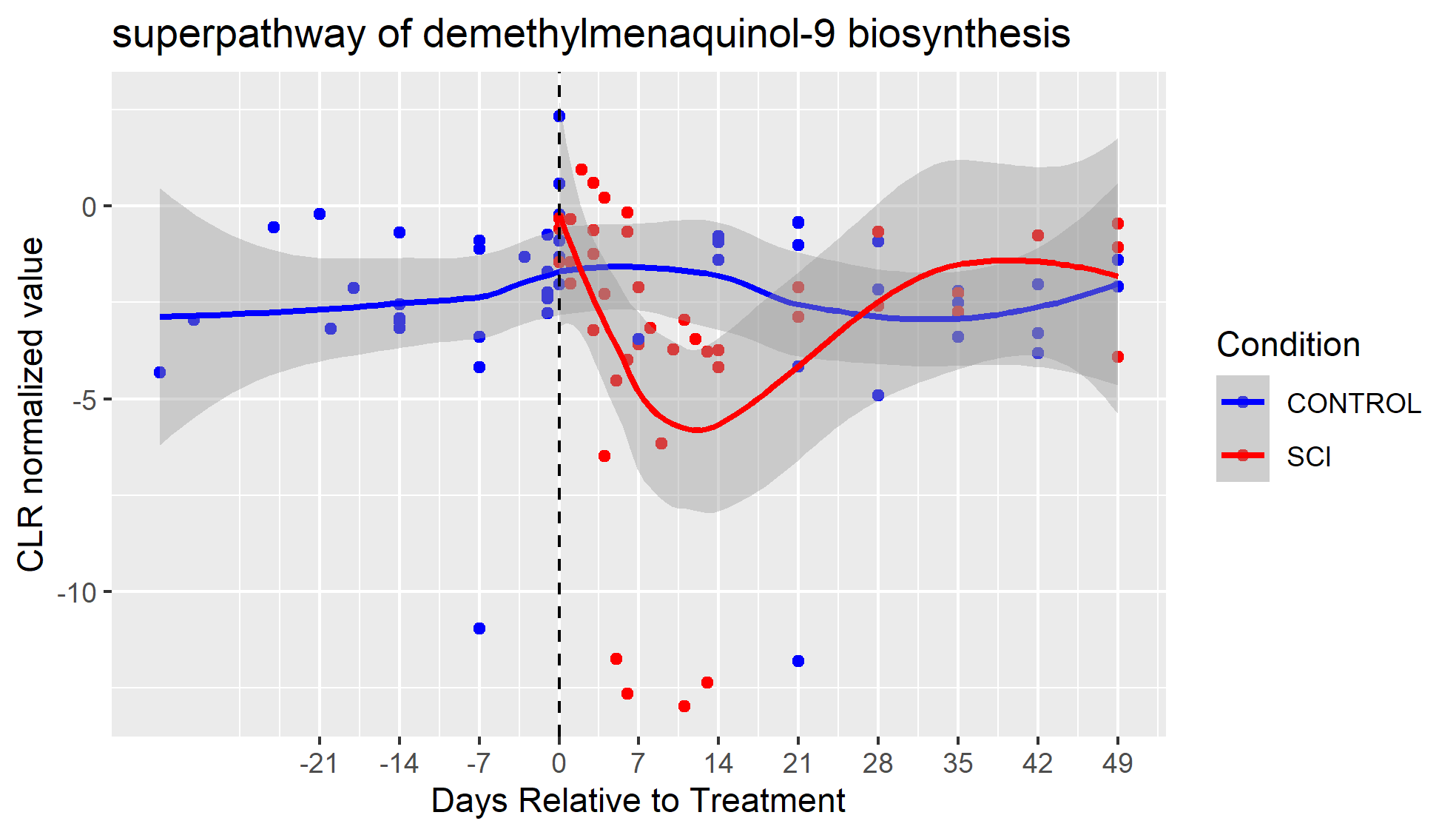

Supplement: Supplementary file 3 — Additional file 3. [file 12864_2021_7979_MOESM3_ESM.zip › pathways_SCI_vs_CONTROL_superpathway_of_demethylmenaquinol-9_biosynthesis.png]

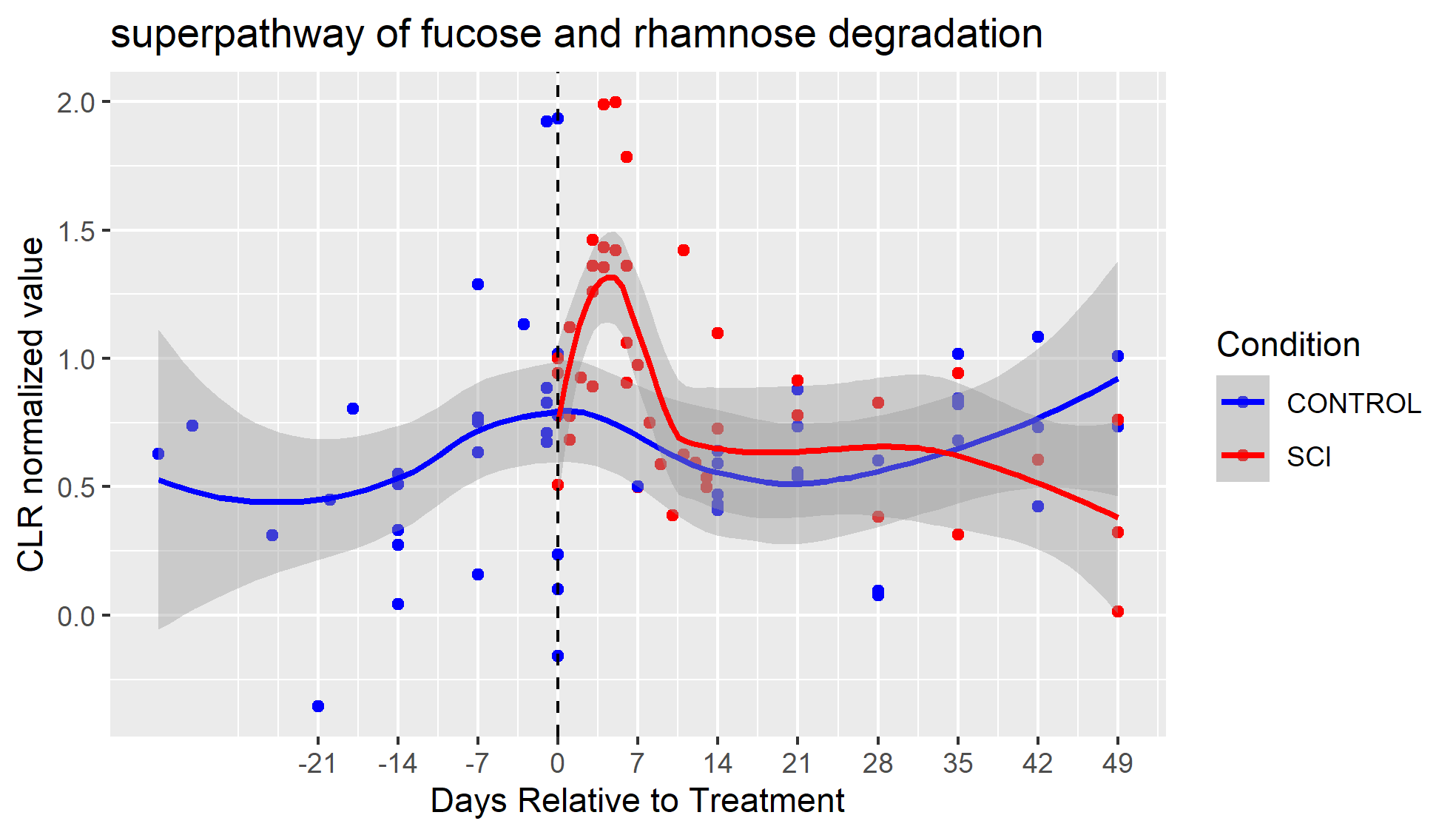

Supplement: Supplementary file 3 — Additional file 3. [file 12864_2021_7979_MOESM3_ESM.zip › pathways_SCI_vs_CONTROL_superpathway_of_fucose_and_rhamnose_degradation.png]

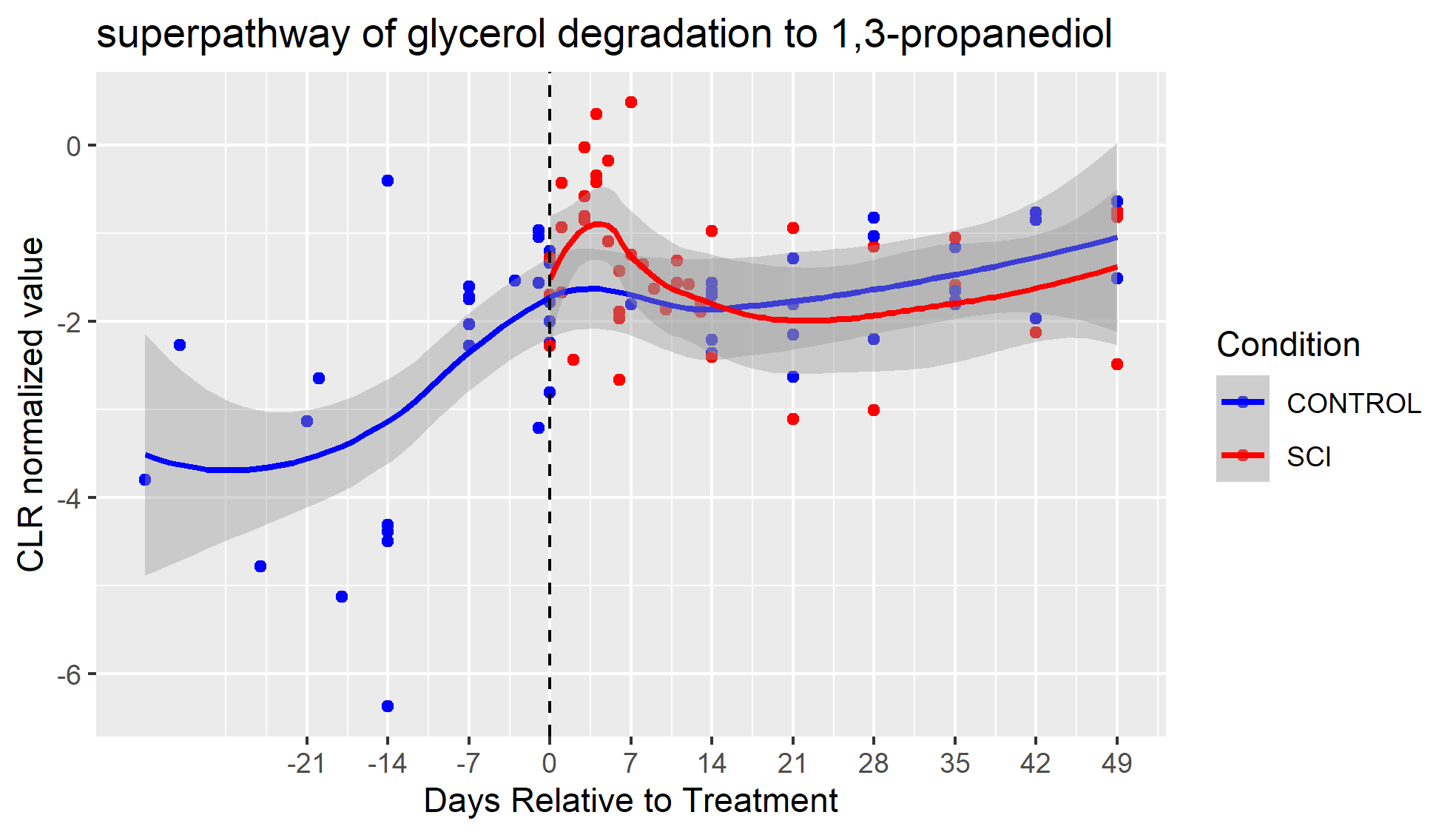

Supplement: Supplementary file 3 — Additional file 3. [file 12864_2021_7979_MOESM3_ESM.zip › pathways_SCI_vs_CONTROL_superpathway_of_glycerol_degradation_to_1,3-propanediol.png]

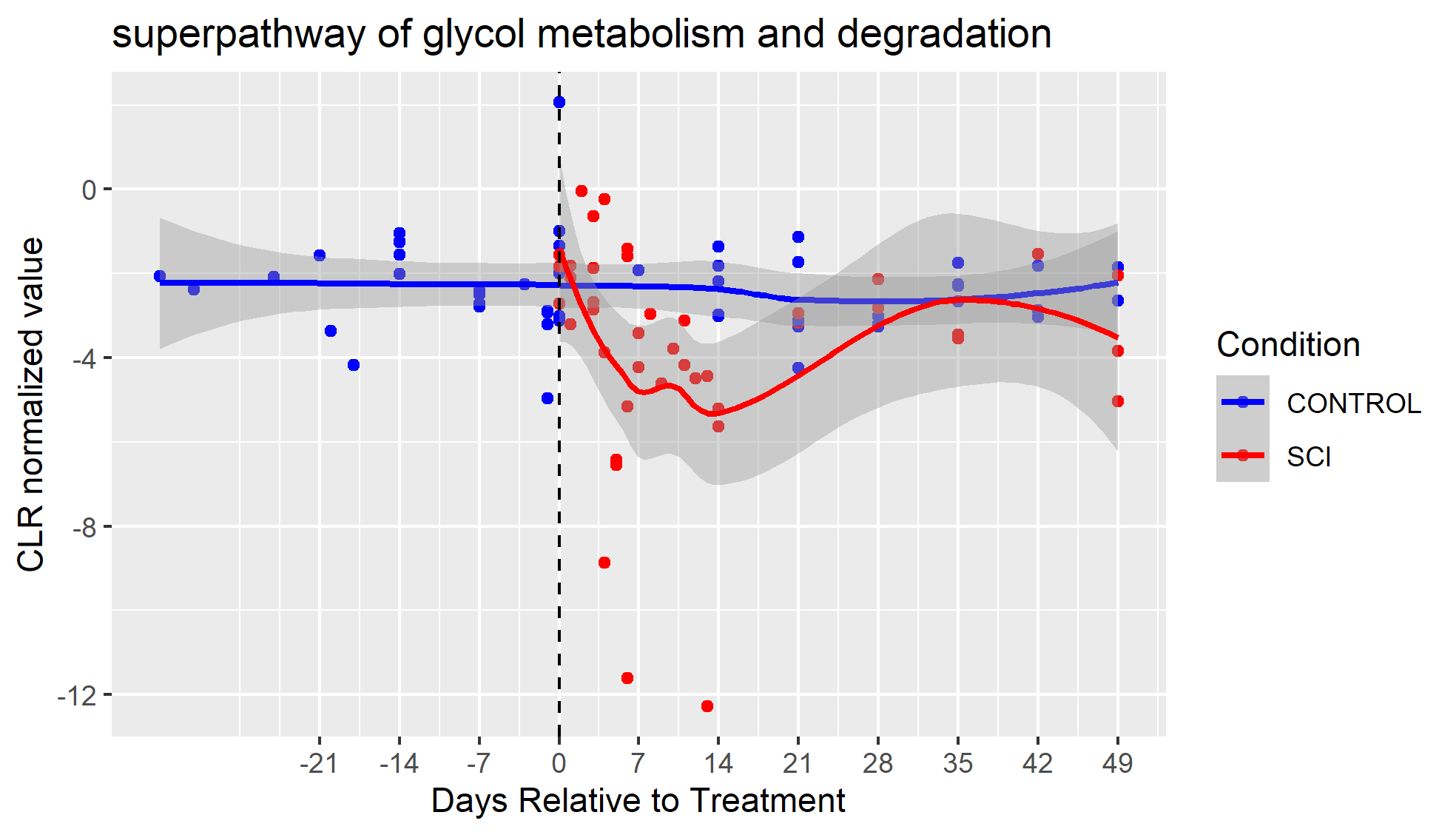

Supplement: Supplementary file 3 — Additional file 3. [file 12864_2021_7979_MOESM3_ESM.zip › pathways_SCI_vs_CONTROL_superpathway_of_glycol_metabolism_and_degradation.png]

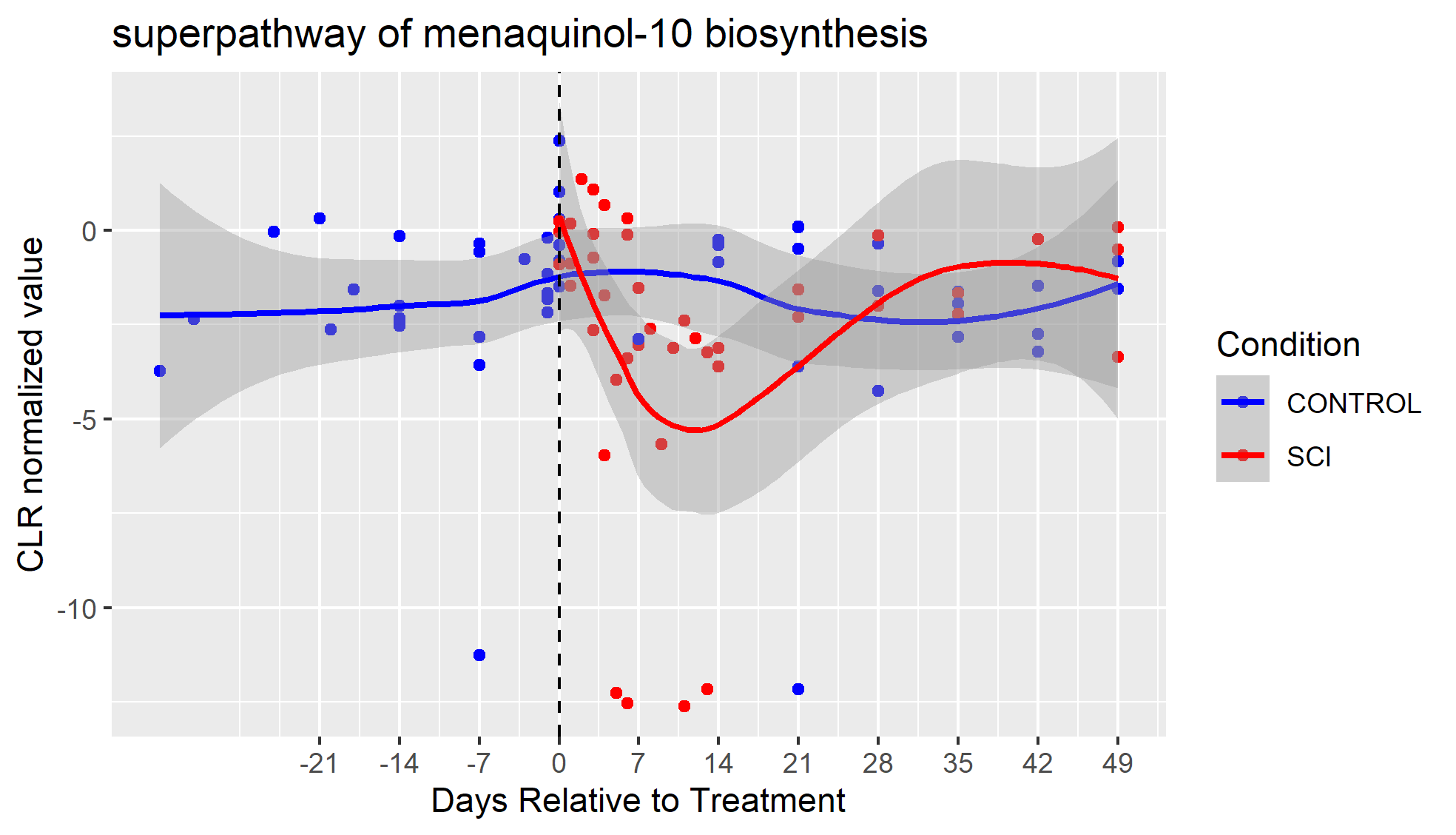

Supplement: Supplementary file 3 — Additional file 3. [file 12864_2021_7979_MOESM3_ESM.zip › pathways_SCI_vs_CONTROL_superpathway_of_menaquinol-10_biosynthesis.png]

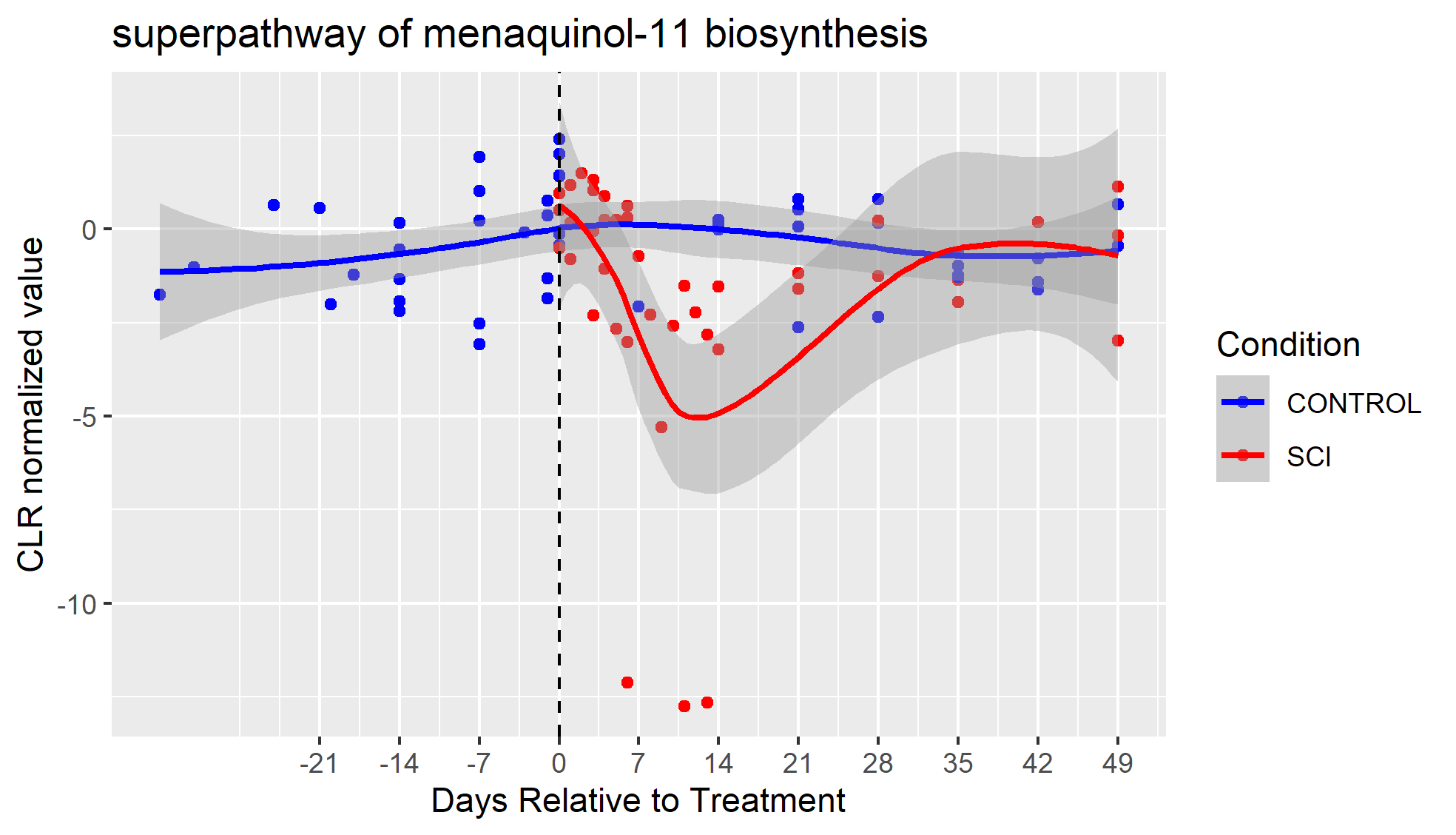

Supplement: Supplementary file 3 — Additional file 3. [file 12864_2021_7979_MOESM3_ESM.zip › pathways_SCI_vs_CONTROL_superpathway_of_menaquinol-11_biosynthesis.png]

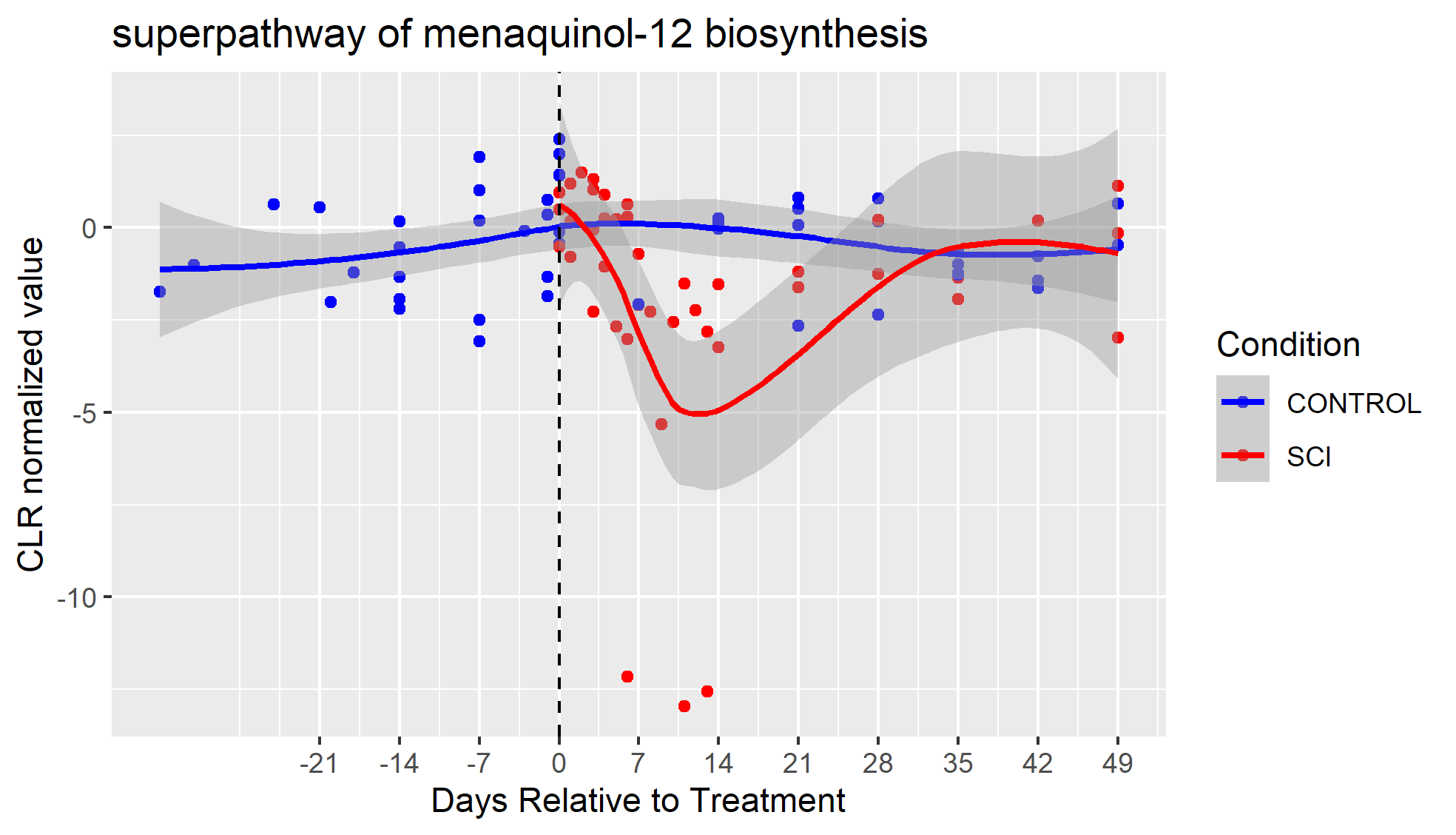

Supplement: Supplementary file 3 — Additional file 3. [file 12864_2021_7979_MOESM3_ESM.zip › pathways_SCI_vs_CONTROL_superpathway_of_menaquinol-12_biosynthesis.png]

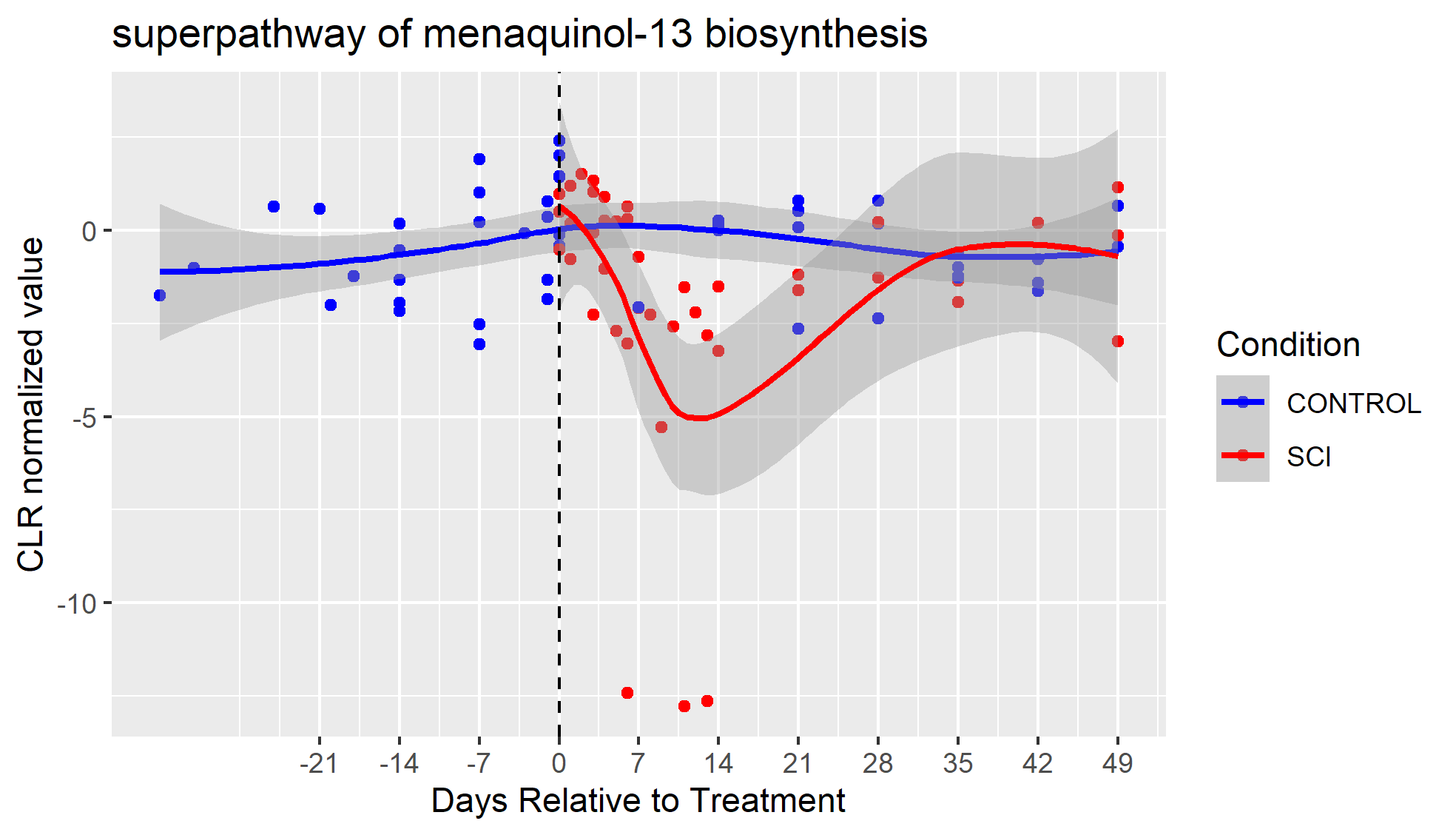

Supplement: Supplementary file 3 — Additional file 3. [file 12864_2021_7979_MOESM3_ESM.zip › pathways_SCI_vs_CONTROL_superpathway_of_menaquinol-13_biosynthesis.png]

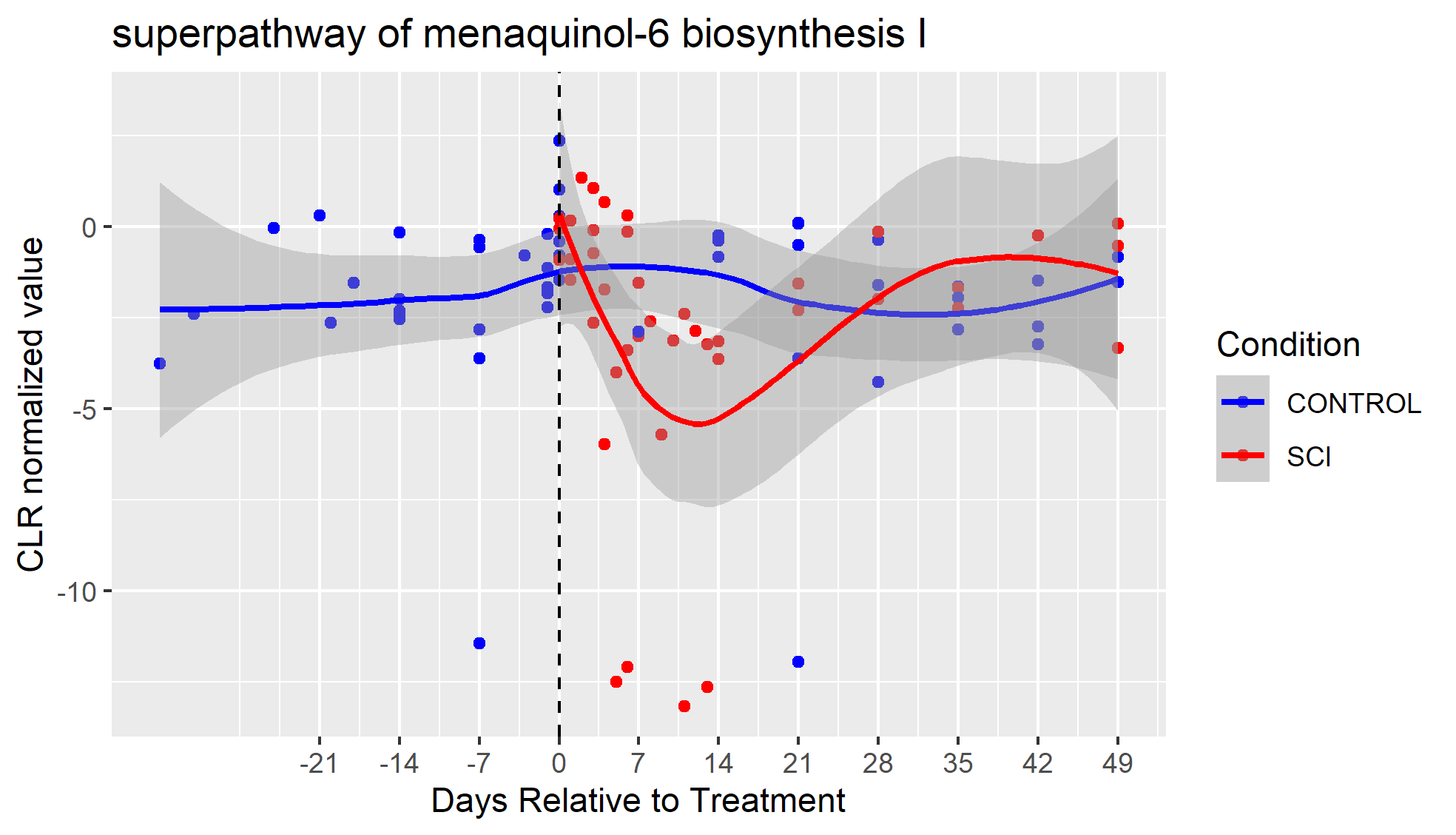

Supplement: Supplementary file 3 — Additional file 3. [file 12864_2021_7979_MOESM3_ESM.zip › pathways_SCI_vs_CONTROL_superpathway_of_menaquinol-6_biosynthesis_I.png]

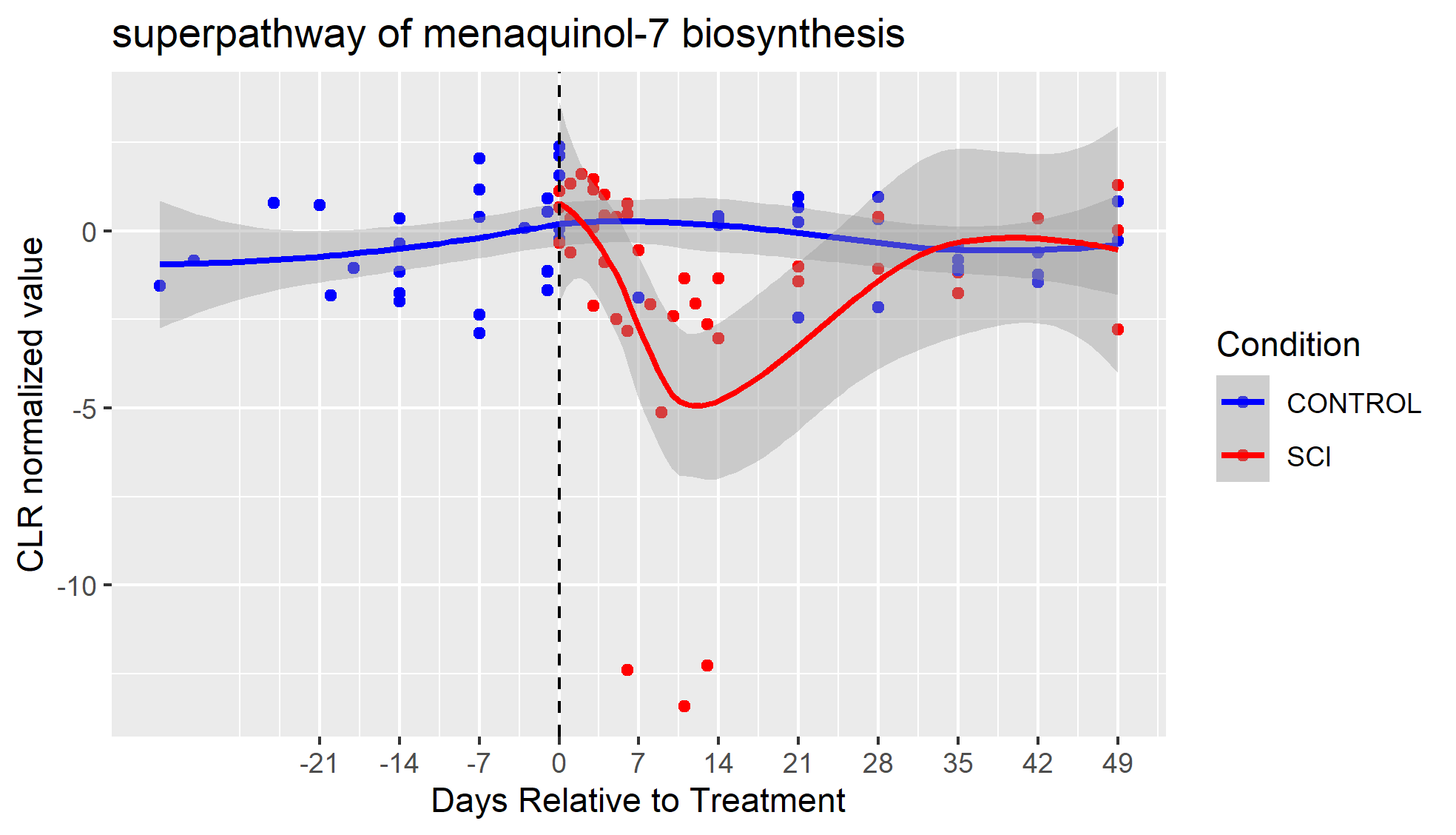

Supplement: Supplementary file 3 — Additional file 3. [file 12864_2021_7979_MOESM3_ESM.zip › pathways_SCI_vs_CONTROL_superpathway_of_menaquinol-7_biosynthesis.png]

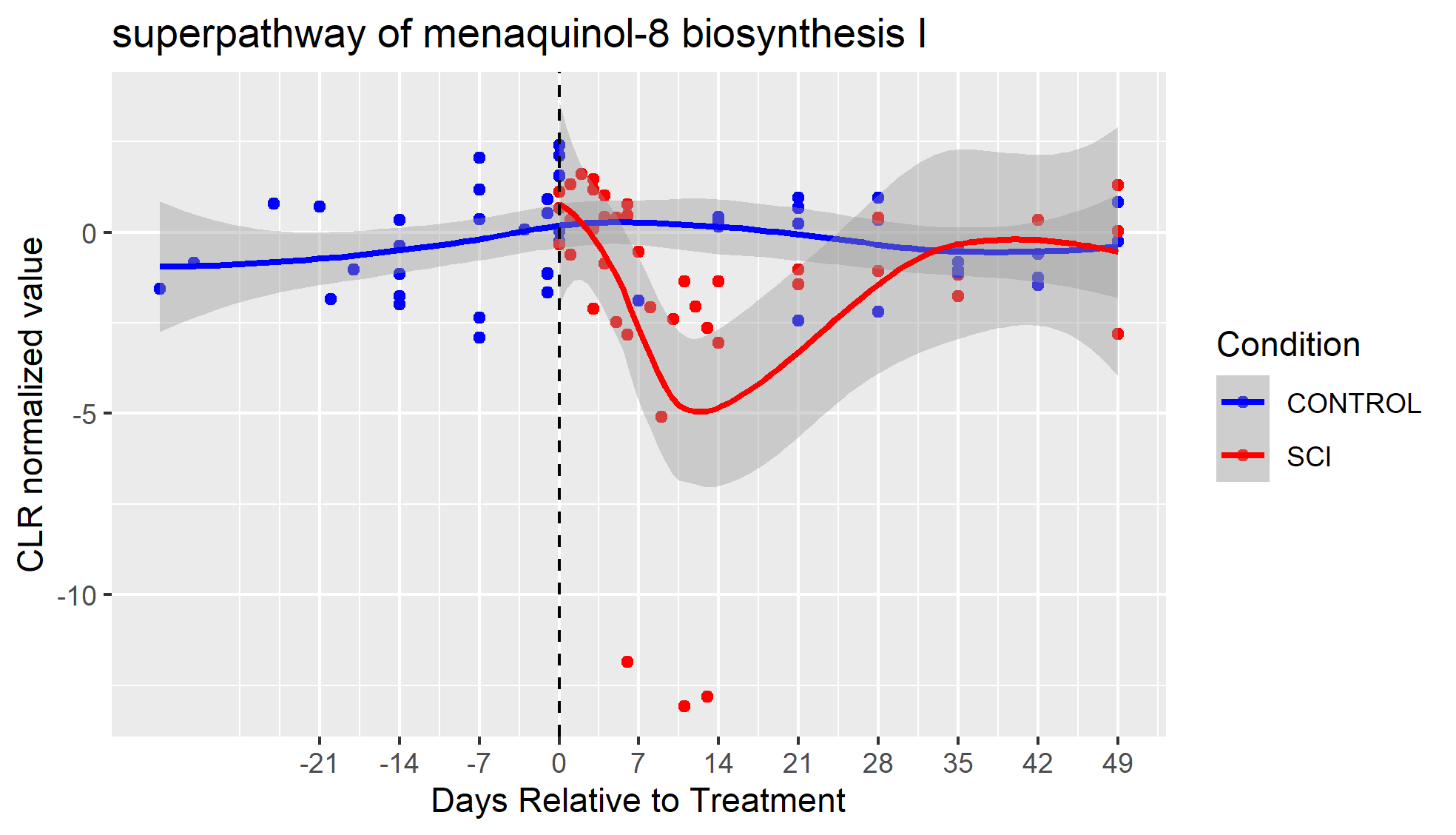

Supplement: Supplementary file 3 — Additional file 3. [file 12864_2021_7979_MOESM3_ESM.zip › pathways_SCI_vs_CONTROL_superpathway_of_menaquinol-8_biosynthesis_I.png]

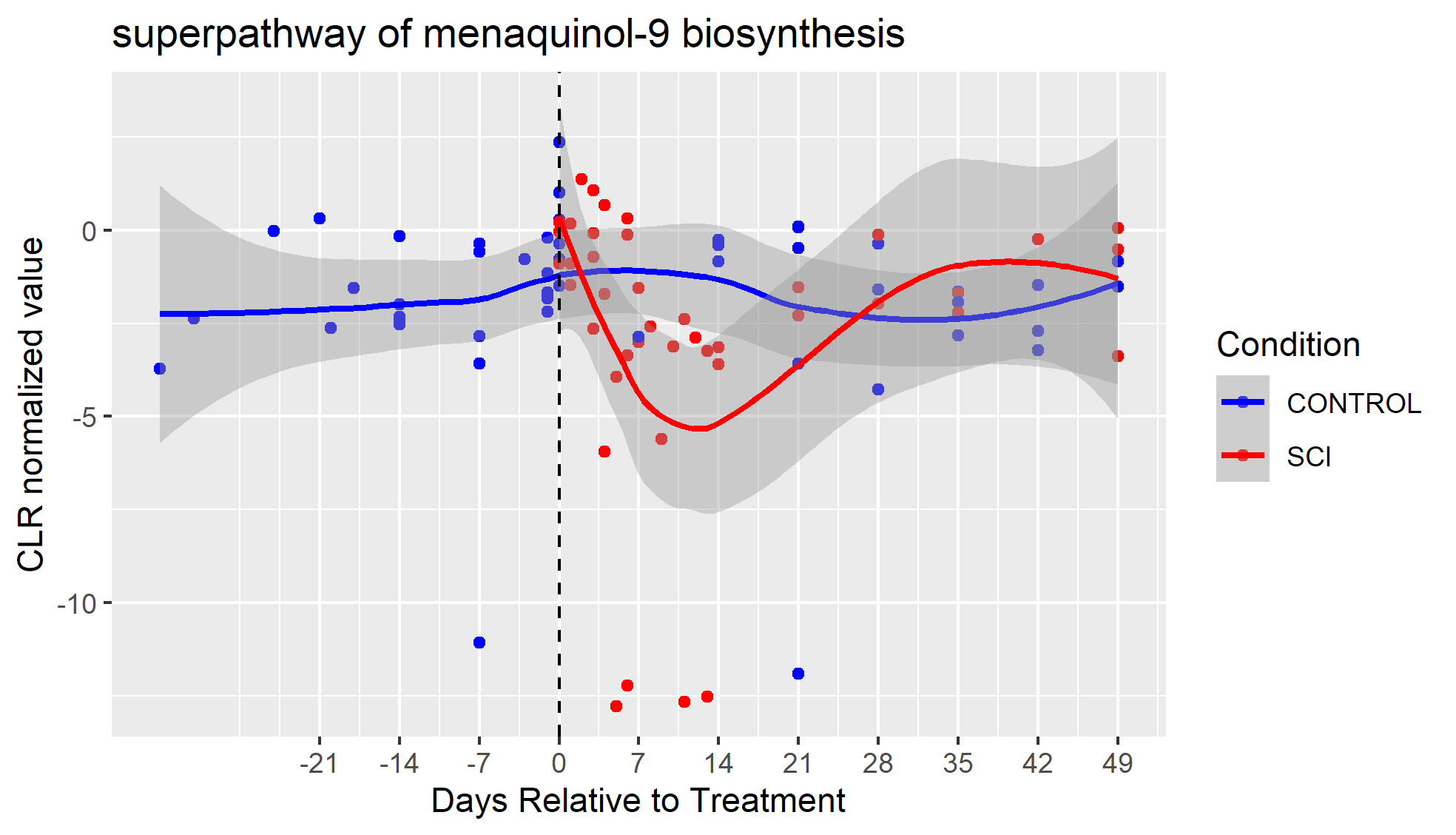

Supplement: Supplementary file 3 — Additional file 3. [file 12864_2021_7979_MOESM3_ESM.zip › pathways_SCI_vs_CONTROL_superpathway_of_menaquinol-9_biosynthesis.png]

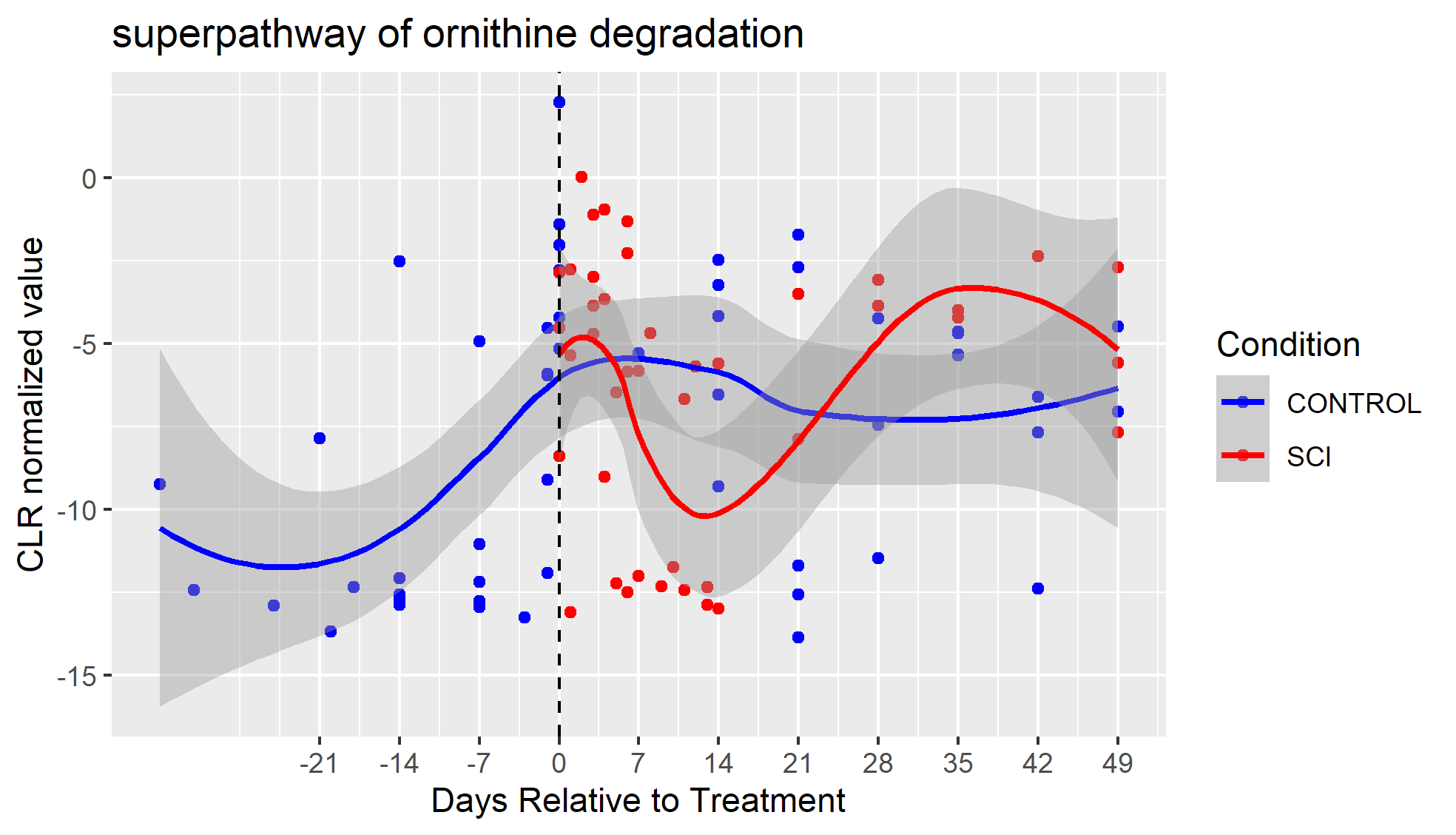

Supplement: Supplementary file 3 — Additional file 3. [file 12864_2021_7979_MOESM3_ESM.zip › pathways_SCI_vs_CONTROL_superpathway_of_ornithine_degradation.png]

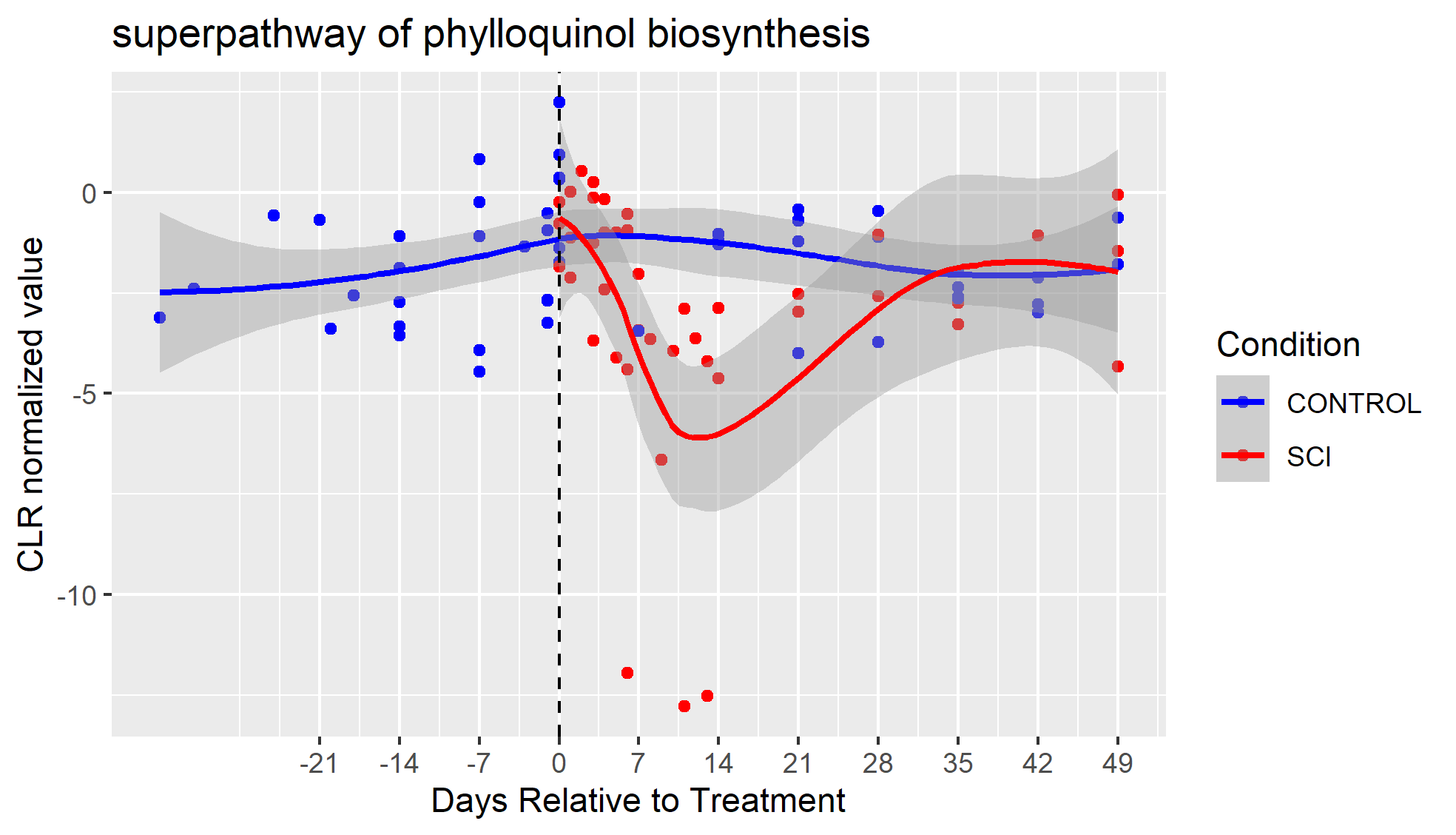

Supplement: Supplementary file 3 — Additional file 3. [file 12864_2021_7979_MOESM3_ESM.zip › pathways_SCI_vs_CONTROL_superpathway_of_phylloquinol_biosynthesis.png]

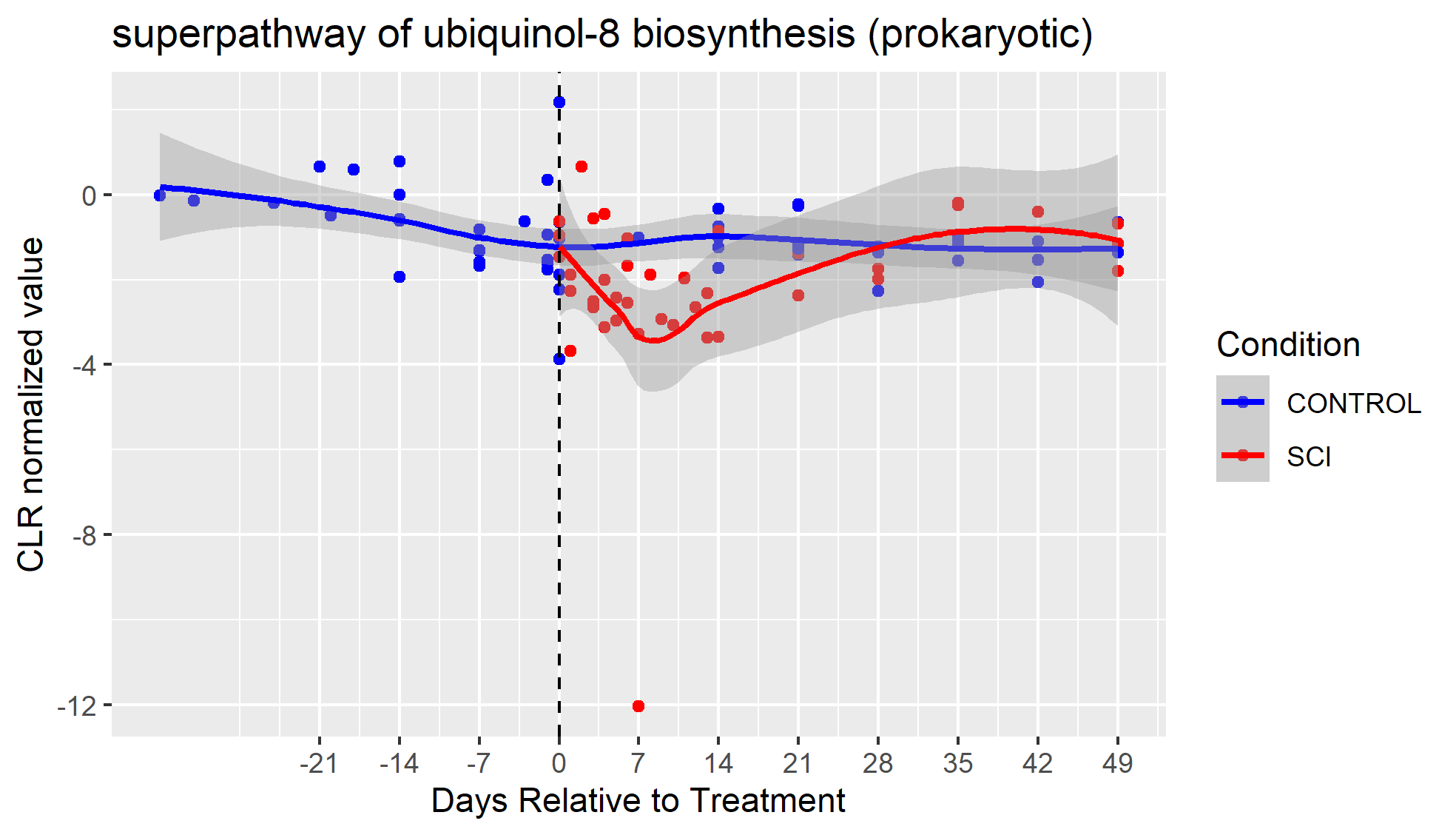

Supplement: Supplementary file 3 — Additional file 3. [file 12864_2021_7979_MOESM3_ESM.zip › pathways_SCI_vs_CONTROL_superpathway_of_ubiquinol-8_biosynthesis_(prokaryotic).png]

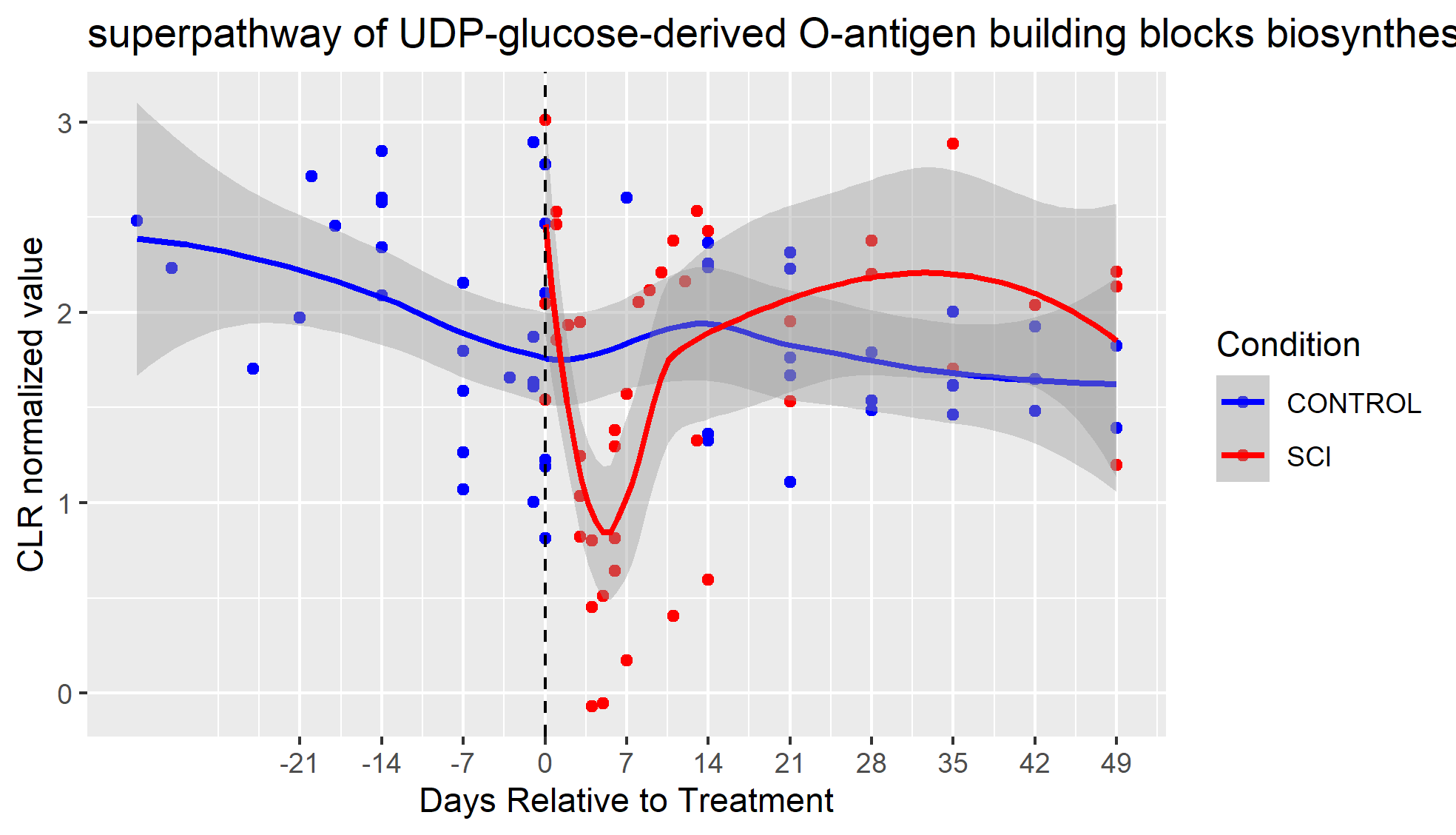

Supplement: Supplementary file 3 — Additional file 3. [file 12864_2021_7979_MOESM3_ESM.zip › pathways_SCI_vs_CONTROL_superpathway_of_UDP-glucose-derived_O-antigen_building_blocks_biosynthesis.png]

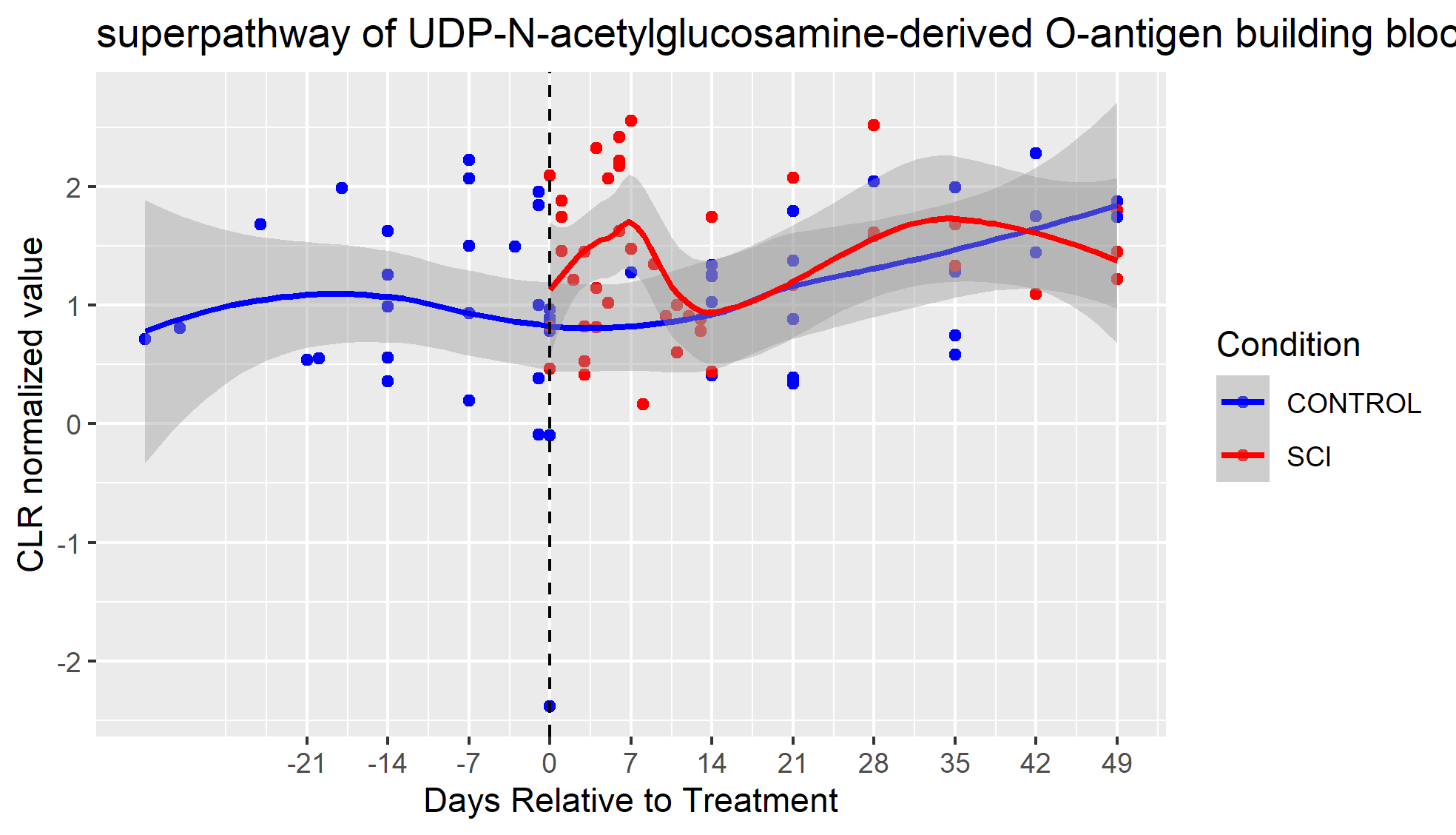

Supplement: Supplementary file 3 — Additional file 3. [file 12864_2021_7979_MOESM3_ESM.zip › pathways_SCI_vs_CONTROL_superpathway_of_UDP-N-acetylglucosamine-derived_O-antigen_building_blocks_biosynthesis.png]

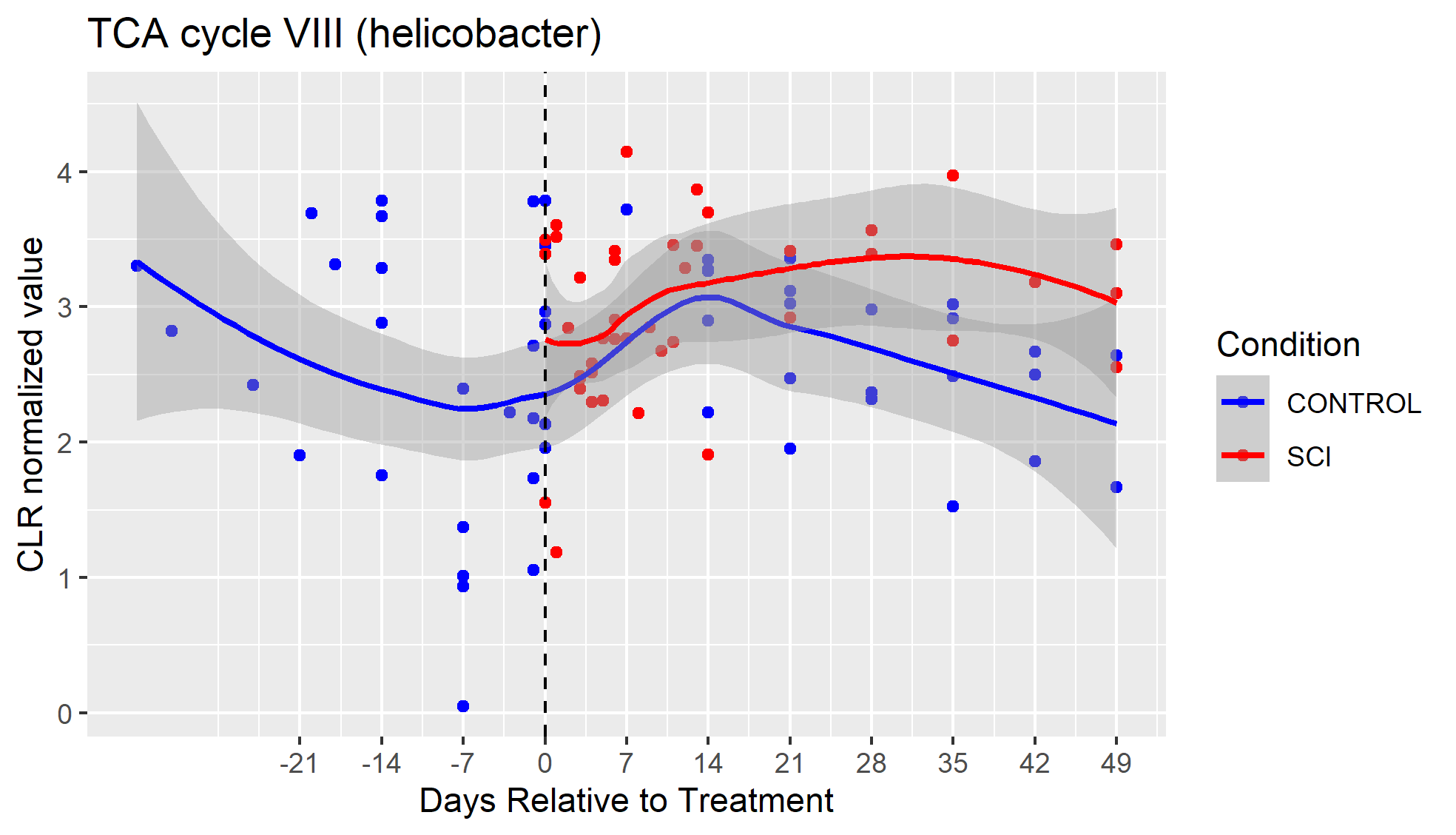

Supplement: Supplementary file 3 — Additional file 3. [file 12864_2021_7979_MOESM3_ESM.zip › pathways_SCI_vs_CONTROL_TCA_cycle_VIII_(helicobacter).png]

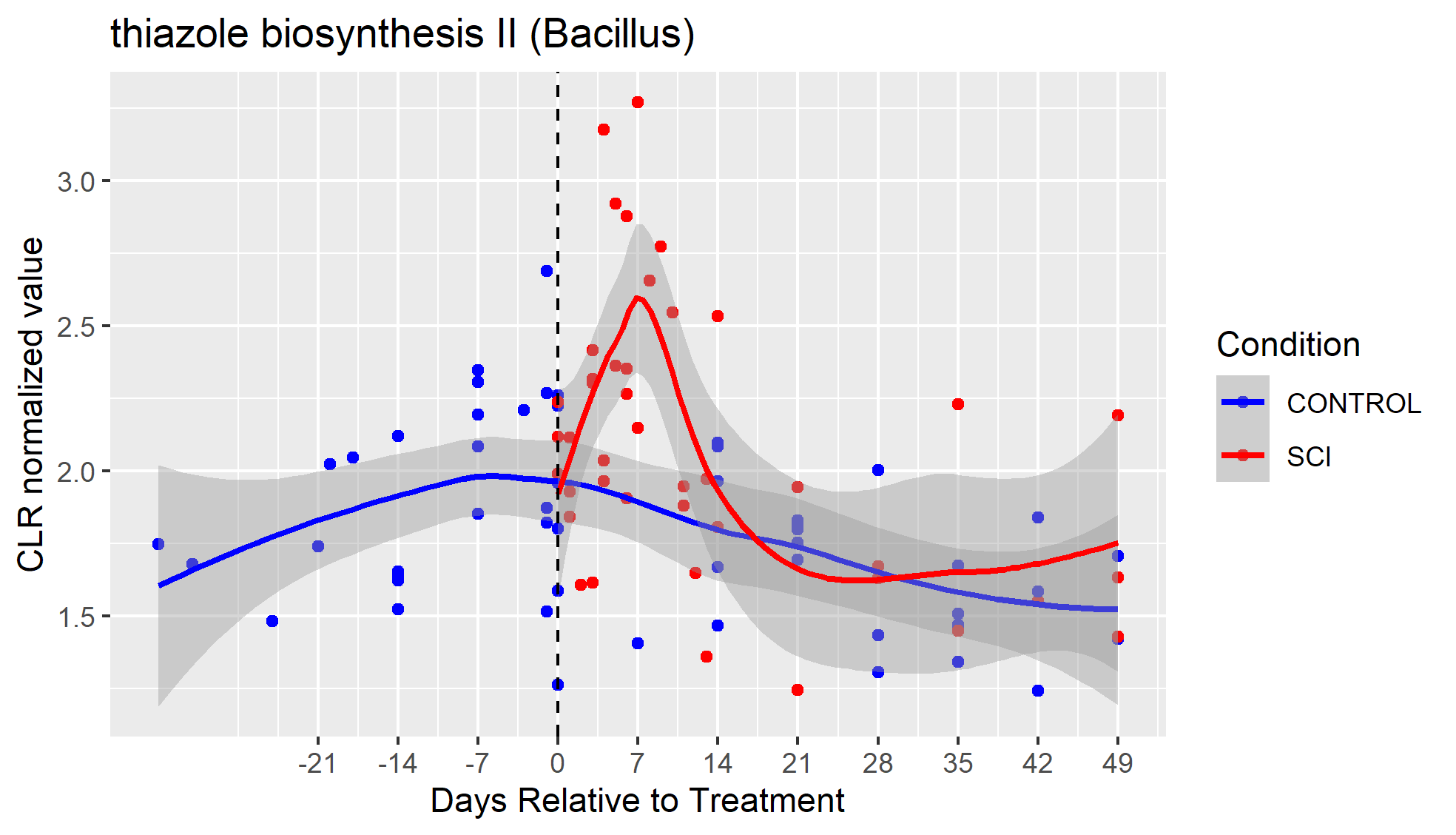

Supplement: Supplementary file 3 — Additional file 3. [file 12864_2021_7979_MOESM3_ESM.zip › pathways_SCI_vs_CONTROL_thiazole_biosynthesis_II_(Bacillus).png]

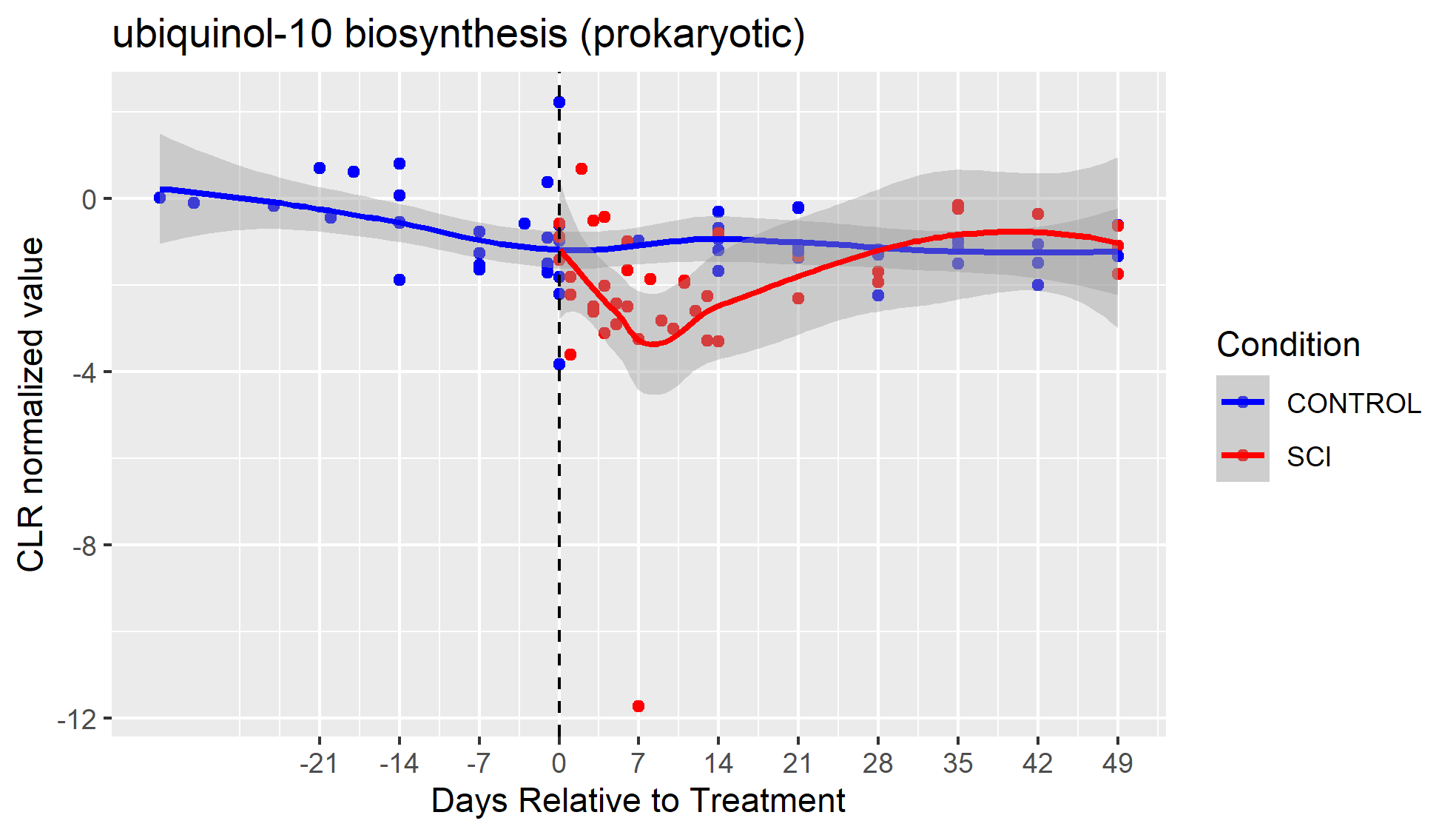

Supplement: Supplementary file 3 — Additional file 3. [file 12864_2021_7979_MOESM3_ESM.zip › pathways_SCI_vs_CONTROL_ubiquinol-10_biosynthesis_(prokaryotic).png]

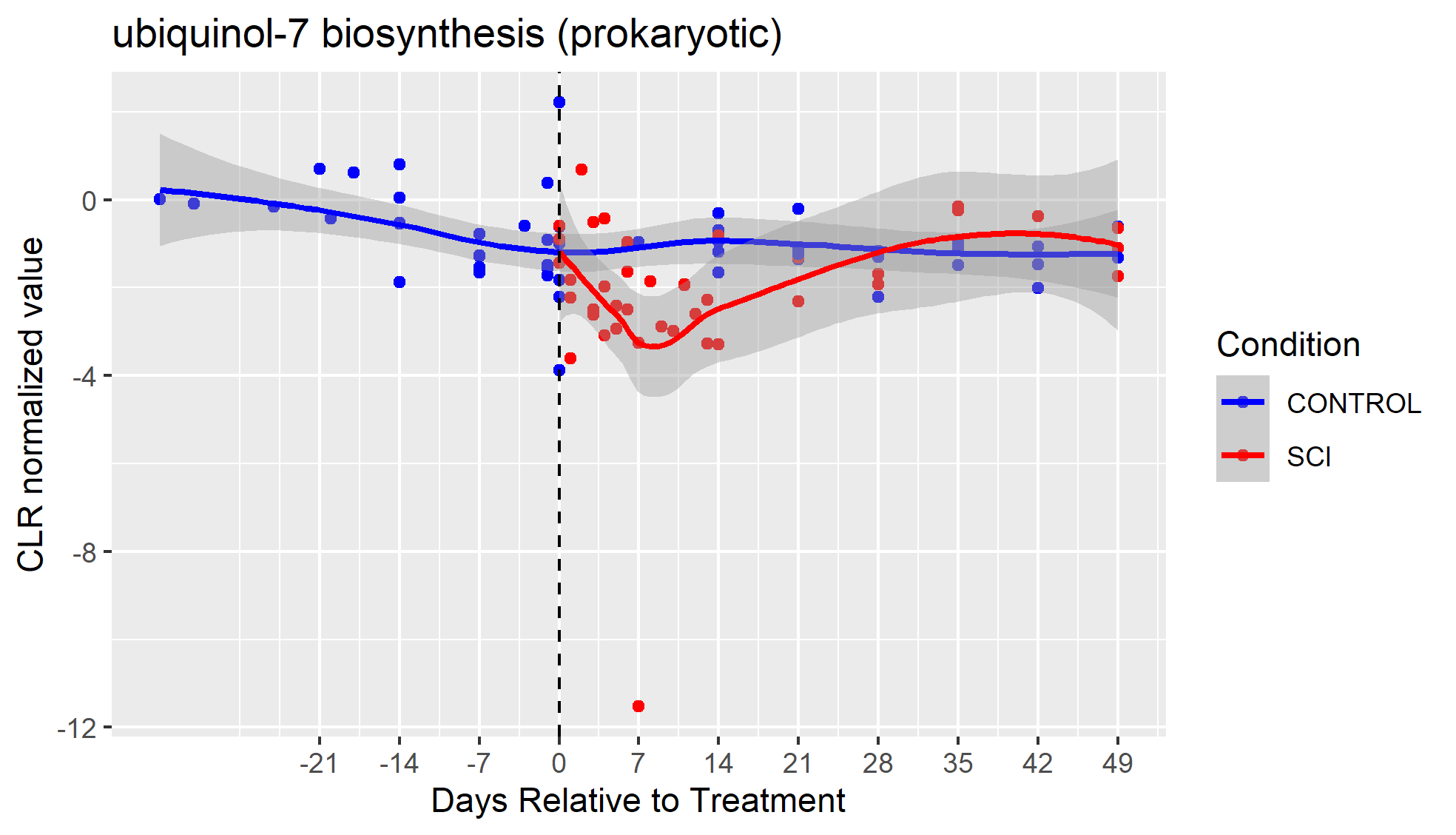

Supplement: Supplementary file 3 — Additional file 3. [file 12864_2021_7979_MOESM3_ESM.zip › pathways_SCI_vs_CONTROL_ubiquinol-7_biosynthesis_(prokaryotic).png]

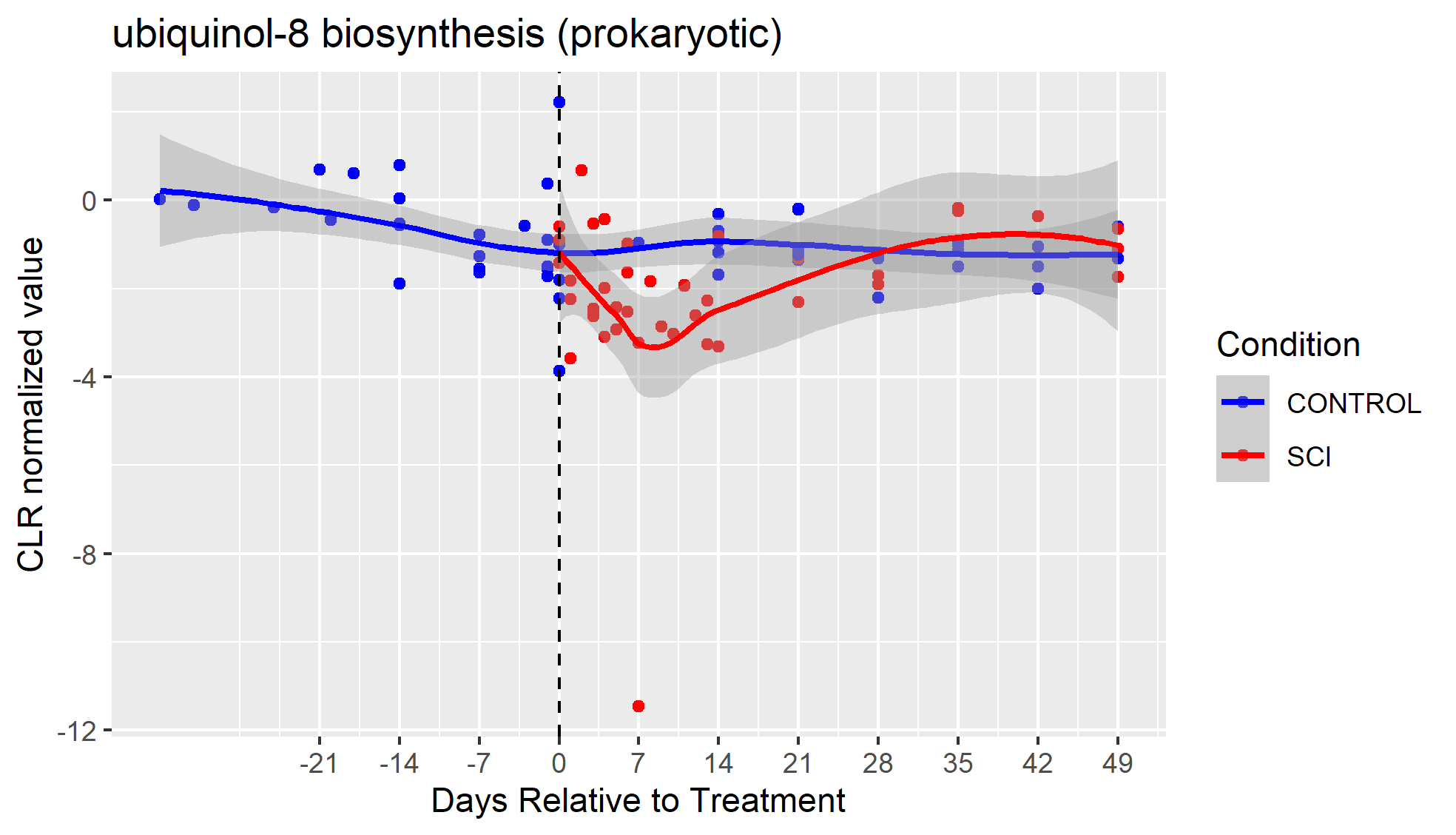

Supplement: Supplementary file 3 — Additional file 3. [file 12864_2021_7979_MOESM3_ESM.zip › pathways_SCI_vs_CONTROL_ubiquinol-8_biosynthesis_(prokaryotic).png]

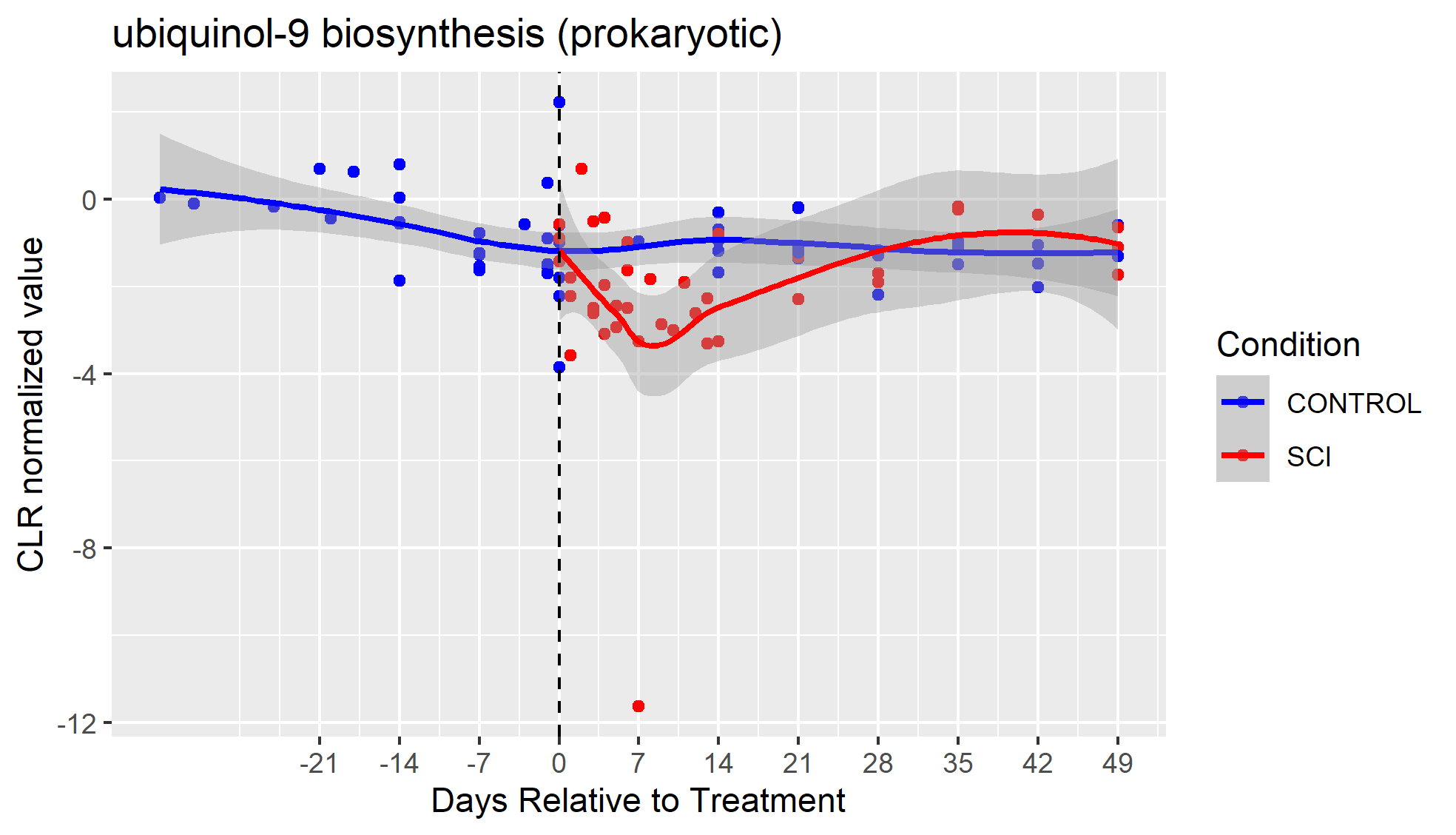

Supplement: Supplementary file 3 — Additional file 3. [file 12864_2021_7979_MOESM3_ESM.zip › pathways_SCI_vs_CONTROL_ubiquinol-9_biosynthesis_(prokaryotic).png]

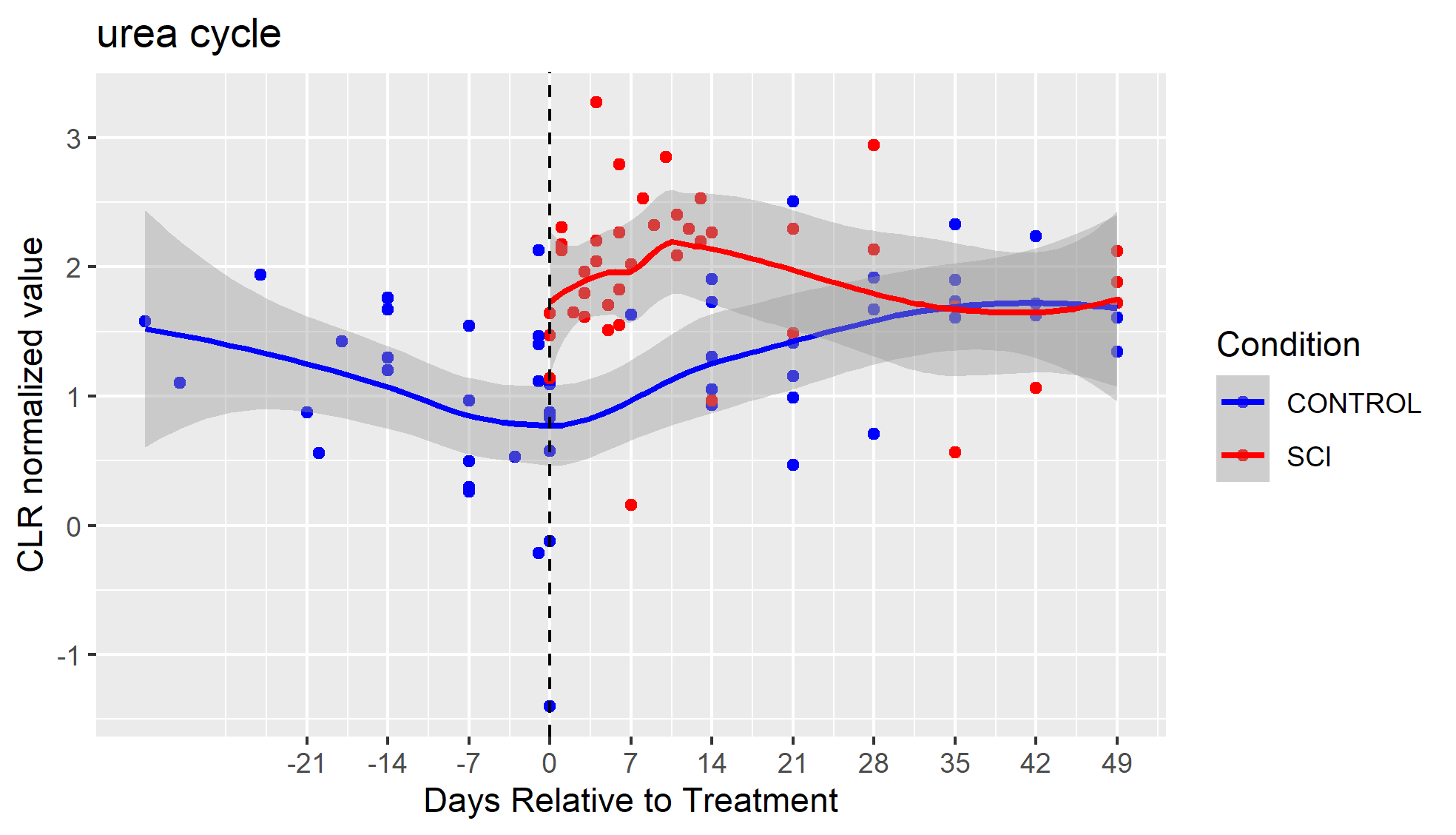

Supplement: Supplementary file 3 — Additional file 3. [file 12864_2021_7979_MOESM3_ESM.zip › pathways_SCI_vs_CONTROL_urea_cycle.png]
